# Identification of Promising RILs for High Grain Zinc Through Genotype × Environment Analysis and Stable Grain Zinc QTL Using SSRs and SNPs in Rice (*Oryza sativa* L.)

**DOI:** 10.3389/fpls.2021.587482

**Published:** 2021-02-18

**Authors:** K. Suman, C. N. Neeraja, P. Madhubabu, Santosha Rathod, Sonali Bej, K. P. Jadhav, J. Aravind Kumar, U. Chaitanya, Smita C. Pawar, Surekha H. Rani, Lella V. Subbarao, Sitapati R. Voleti

**Affiliations:** ^1^ICAR–Indian Institute of Rice Research, Hyderabad, India; ^2^Department of Genetics & Biotechnology, Osmania University, Hyderabad, India

**Keywords:** rice, grain zinc, RILs, stability, QTL, SSR, SNP

## Abstract

Polished rice is one of the commonly consumed staple foods across the world. However, it contains limited nutrients especially iron (Fe) and zinc (Zn). To identify promising recombinant inbred lines (RILs) for grain Zn and single plant yield, 190 RILs developed from PR116 and Ranbir Basmati were evaluated in two environments (E1 and E2). A subset of 44 contrasting RILs for grain Zn was screened in another two environments (E3 and E4). Phenotypic data was collected for 10 traits, viz., days to 50% flowering, plant height, panicle length, number of tillers, single plant yield (SPY), test weight, Fe and Zn in brown (IBR, ZBR), and polished rice (IPR, ZPR). Stepwise regression analysis of trait data in 190 RILs and a subset of 44 RILs revealed the interdependence of ZPR, ZBR, IPR, and IBR and the negative association of grain Zn with single plant yield. Based on the additive main effect and multiplicative interaction (AMMI) and genotype and genotype × environment interaction (GGE) analyses of the subset of 44 RILs across four environments (E1–E4), six promising RILs were identified for ZPR with >28 ppm. Mapping of 190 RILs with 102 simple sequence repeats (SSRs) resulted in 13 QTLs for best linear unbiased estimates (BLUEs) of traits including advantage over check (AOC). Using genotype-based sequencing (GBS), the subset of 44 RILs was mapped with 1035 single-nucleotide polymorphisms (SNPs) and 21 QTLs were identified. More than 100 epistatic interactions were observed. A major QTL *qZPR.1.1* (PV 37.84%) and another QTL *qZPR.11.1* (PV 15.47%) were identified for grain Zn in polished rice. A common major QTL (*qZBR.2.1* and *qZPR.2.1*) was also identified on chromosome 2 for grain Zn content across SSR and SNP maps. Two potential candidate genes related to transporters were identified based on network analyses in the genomic regions of QTL < 3 Mb. The RILs identified for grain Zn and SPY were nominated for national evaluation as under rice biofortification, and two QTLs identified based on BLUEs could be used in the rice biofortification breeding programs.

## Introduction

Rice (*Oryza sativa* L.) is an important staple food for several countries across the world. More than 50% of the calorific needs are met by rice for most of the population in many Asian countries^1^. While brown rice (unpolished) is a good source of nutrients and vitamins, polished rice which is devoid of most of the important nutrients is the most preferred form of consumption (Nachimuthu et al., 2015; Pradhan et al., 2020; Rao et al., 2020). Most of the modern high-yielding rice varieties are reported to be poor in nutrient content after polishing (Anandan et al., 2011; Swamy et al., 2016). Hence, improvement in the nutritive value of polished rice would have a direct impact the nutrition security of consumers who are excessively or solely dependent on rice.

Micronutrient malnutrition or hidden hunger is widely spread in developing countries, especially among poor populations, whose daily caloric intake is mainly confined to staple cereals (White et al., 2012; Cakmak and Kutman, 2018; Garcia-Oliveira et al., 2018). Dietary diversification, supplementation, and postharvest food fortification are some of the possible important strategies to address malnutrition (Bouis et al., 2019). Of the various strategies to address malnutrition, biofortification is one of the promising approaches because of its sustainability and affordability (Bouis and Welch, 2010). Biofortification is the process of increasing the density of vitamins and minerals in a crop through plant breeding using conventional methods or genetic engineering or through agronomic practices. Breeding rice varieties with higher mineral densities can help in tackling hidden hunger in most of the Asian countries (Bouis et al., 2013; Swamy et al., 2016).

Iron (Fe) and zinc (Zn) are critical among the essential micronutrients required for human health. More than two billion people across the world, mostly children and pregnant and lactating women, suffer from Fe and Zn deficiencies (Wessells and Brown, 2012). Fe is the main component of hemoglobin and is important for oxygen transport, DNA synthesis, and electron transport (Abbaspour et al., 2014). Zn is required for the functioning of >300 enzymes and >1000 transcription factors and is the second messenger of immune cells in the human body (Prasad, 2013). For plants, also Fe and Zn are vital elements promoting plant growth and development (Grotz and Guerinot, 2006; Teklić et al., 2013). For Fe, a wide range of genetic variation ranging from 2 to 147 ppm was reported in brown rice (Zeng et al., 2010). With the polishing losses of Fe up to 85%, the observed variability for Fe in polished rice was found to be very limited (Prom-u-thai et al., 2007; Sperotto et al., 2012).

A wide genetic variability for grain Zn content is reported in brown and polished rice in the germplasm. Zn content in brown rice ranged from 7.3 to 58.4 ppm, and that in polished rice ranged from 4.8 to 40.9 ppm with polishing losses of 11.1 to 28% (Rao et al., 2020).

Rice grain Zn content is a quantitative trait and is dependent on several processes such as uptake from soil, assimilation within the plant, and remobilization into the grain (Sperotto et al., 2014; Garcia-Oliveira et al., 2018). It is also influenced by environmental factors such as soil and water (Wissuwa et al., 2008). Thus, developing Zn-biofortified rice varieties is difficult owing to the complex genetics, environment effects, and genetic interactions such as epistasis (Zhang et al., 2014; Pradhan et al., 2020). Characterization of promising breeding lines for high grain Zn by multivariate techniques such as biplots, Additive Main effects and Multiplicative Interaction (AMMI) and Genotype main effects, and G × E interaction effects (GGE) would partition the G × E interactions for the target trait (Gauch and Zobel, 1997; Li et al., 2017). Identification of markers linked with quantitative trait loci (QTL) for grain Zn content would expedite the development of rice biofortified varieties through marker-assisted breeding (Neeraja et al., 2017). QTL mapping provides opportunities for identification of the genomic region(s) associated with the targeted traits by combining genome information with phenotyping. Subsequently identified genomic region(s)/QTLs/genes could be deployed in the breeding programs through marker-assisted selection (MAS) (Collard and Mackill, 2008). Several major and stable QTLs through MAS have been introgressed in rice toward the development of varieties with target traits (Hasan et al., 2015).

Using bi-parental mapping populations, 22 independent studies have reported 220 QTLs for grain Fe and Zn in rice using simple sequence repeat (SSR) markers or candidate gene-based markers (Raza et al., 2019). Interactions among the identified QTLs for grain Zn were also studied for characterization of QTL interaction effects on the phenotypic expression of trait (Swamy et al., 2018a; Descalsota-Empleo et al., 2019a; Calayugan et al., 2020). Single-nucleotide polymorphisms (SNPs) are now being preferred as markers not only because of their abundance and uniform distribution in the genome but also for their precision, speed, and low cost (He et al., 2014; Varshney et al., 2018). Genotyping-by-sequencing (GBS) is a modified highly efficient and cost-effective approach for simultaneous genome-wide SNP discovery and genotyping (Elshire et al., 2011). A modified GBS technique is based on two restriction enzymes comprising rare cutting and frequently cutting as double-digest restriction-site-associated DNA sequencing (ddRAD-seq) for enhancing the stability of selected genomic regions (Peterson et al., 2012). GBS is being deployed in many crop genomics studies (Gali et al., 2019; Sudan et al., 2019; Dissanayaka et al., 2020) and also in rice (De Leon et al., 2016; Furuta et al., 2017; Bhatia et al., 2018; Yadav et al., 2019; Babu et al., 2020).

From the reported mapping studies for grain Zn in rice, numerous genetic loci with minor to major effects were found to be distributed throughout the genome. Hence, we selected SNPs as the markers of choice due to their abundant distribution across the chromosomes in addition to the SSR markers for identification of QTLs for grain Zn and Fe along with yield and yield-related traits in a RIL population derived from PR116 and Ranbir Basmati. Thus, the objectives of the present study were i. to characterize the variability of the RIL population across environments for their grain Zn and Fe content, yield, and agro-morphological traits; ii. to assess the association and relationship among the traits for grain Zn and yield; iii. to identify stable lines from the RIL population for grain Zn and yield across environments; and iv. to identify QTLs for grain Zn, yield, and agro-morphological traits using SSRs and SNPs along with interaction and environment effects.

## Materials and Methods

### Field Experimental Details

The experiments were performed in the Indian Council of Agricultural Research (ICAR)-Indian Institute of Rice Research (IIRR), Hyderabad, India (17.53°N latitude and 78.27°E longitude, 545 mm rainfall), during four environments [Environment 1 wet season (WS) 2016; Environment 2 dry season (DS) 2017; Environment 3 WS 2017; and Environment 4 DS 2018]. The details of temperature, rainfall, and soil characteristics were given in Supplementary Table 1. A set of 190 RILs with two parents was grown during Environment 1 (E1) and Environment 2 (E2), and a subset of 44 contrasting RILs for grain Zn with two parents was grown during Environment 3 (E3) and Environment 4 (E4) following randomized complete block design (RCBD). Recommended packages of rice crop production and protection practices were followed for raising healthy crop.

### Plant Material

A recombinant inbred population (RIL) was developed from the cross between PR116 (pedigree: PR108/PAU 1628//PR108; released in 2000; mean yield of 5–6 tons/ha; Zn: 19.1 ppm in brown rice; 15.7 ppm in polished rice) as recipient parent for grain Zn and Ranbir Basmati (pure line selection from Basmati 370-90-95; released in 1996; mean yield of 2–2.5 tons/ha; Zn: 27.5 ppm in brown rice; 23.4 ppm in polished rice) as donor parent for high grain Zn using the single seed descent (SSD) method. Each RIL was planted in four rows, and each row consisted 15 plants with a spacing of 20 × 15 cm. Five uniform tagged plants from center rows were considered for observation across all environments.

### Measurement of Phenotypic Traits

Observations for RILs and parents were noted for days to 50% flowering (DFF), plant height (PH), number of tillers per plant (NT), panicle length (PL) and single plant yield (SPY) along with the grain Zn and Fe in brown and polished rice.

### Estimation of Grain Zn and Fe

For estimation of grain Zn in brown rice (ZBR), Fe in brown rice (IBR), Zn in polished rice (ZPR), and Fe in polished rice (IPR), seed samples were dehusked using the JLGJ4.5 rice husker (Jingjian Huayuan International Trade Co., Ltd., China) and polished with a polisher with non-ferrous components (Krishi International India Ltd., India). The seed from five plants was pooled and divided into three parts for analyses as three replicates. After thorough cleaning, each sample of brown and polished rice (5 g) was analyzed for Fe and Zn by energy dispersive X-ray fluorescent spectrophotometer (ED-XRF) as per the standardized protocols (Rao et al., 2014). In rice, promising breeding lines are selected based on their significant yield advantage over the check variety (Virk et al., 1996; Witcombe et al., 2013). Based on the same concept, in the present study, we considered advantage of over check (AOC) for, viz., grain Zn and Fe content over donor parent (with high grain Zn and Fe content) and grain yield over recipient parent (with high single plant yield). The advantage of grain Zn and Fe content and yield in RILs were calculated as advantage over check (AOC) percentage.

### Grain Quality Characters

Two hundred and fifty grams of brown rice of each RIL along with parents harvested during DS2018 was analyzed for 13 grain quality traits/parameters, viz., HULL (hulling per cent), MILL (milling per cent), HRR (head rice recovery per cent), KL (kernel length in mm), KB (kernel breadth in mm), KL/KB (kernel length/breadth ratio), VER (volume expansion ratio in mm), WU (water uptake in mL), KLAC (kernel length after cooking in mm), ER (elongation ratio in mm), ASV (alkali spreading value), AC (amylose content per cent), and GC (gel consistency in mm) by standard protocols at ICAR-IIRR. KL, KB, and KL/KB were determined by vernier calipers (Yadav and Jindal, 2007). HULL and MILL were recorded using a dehusker and miller (Satake Corporation, Japan). The HRR (Khush et al., 1979), WU, KLAC, ER, VER, ASV, AC, and GC were measured by standard methods (Juliano, 1971; Fitzgerald et al., 2009; Suman et al., 2020).

### Quantification of Grain Phytic Acid and Inorganic Phosphorous (Pi)

Grain phytic acid and inorganic phosphorous were determined as described by Lorenz et al. (2007) with minor modifications. To 100 mg grain powder of polished rice from each RIL in a 2-ml Eppendorf tube, 2 ml of 0.65 M HCl was added. The tubes were shaken overnight at room temperature at 120 rpm and centrifuged at 12,000 rpm for 5 min. For estimation of phytic acid, 500 μl of the above extract was transferred to a fresh 2-ml Eppendorf tube and the same quantity was also transferred to a 15-ml tube for estimation of inorganic phosphorus. Equal volumes of, viz., phytic acid dodecasodium salt from rice (Sigma) and KH_2_PO_4_ (HiMedia) for inorganic phosphorous, were used as quantitative standards.

For the estimation of inorganic phosphorus, 1 ml of Pi reagent (consisting of two parts of distilled H_2_O, one part each of 0.02 M ammonium molybdate, 0.57 M ascorbic acid, and 3 M sulfuric acid) and 1 ml of distilled H_2_O were added to each tube. The blue color developed after 15 to 20 min of incubation at room temperature was measured at 820 nm. For measurement of phytate, 1.25 ml of Wade reagent (0.3 g 5-sulfosalicylic acid, 0.03 g FeCl_3_⋅6H_2_O with pH adjusted to 3.05 and made up to a final volume of 100 ml with distilled H_2_O) was added and incubated for 15 min at room temperature and a pink color developed was measured at the optical density at 490 nm. Phytate was converted to phytate P by dividing phytate by a factor of 3.55 (Raboy and Dickinson, 1984).

### Statistical Analyses

Analysis of variation (ANOVA) was calculated for individual environments and also for combined data of four environments (E1–E4). Descriptive statistics viz., mean, standard error of mean (SEM), skewness, kurtosis, and coefficient variations (*CV* %), were calculated. Trait-wise frequency distribution and box plots of individual environments and combined data were illustrated using R software (R Core Team, 2018). Correlation analysis was carried out in SAS (version 9.3) available at ICAR-IIRR, Hyderabad. Best linear unbiased estimates (BLUEs) were generated by setting random environment effects and fixed genotype effects (Gregorio et al., 2015—META-R, Version 6.04). The values of BLUEs were used to perform QTL analysis. Different R packages viz., ggplot2, stability, gge, agricolae, GGEBiplot biotools, FactoMineR, and factoextra, were used to generate frequency distribution plots, box plots, G × E interaction effects (GGE) biplots, additive main effects and multiplicative interaction (AMMI) biplots, environment and ranking of RILs, mean vs. stability and Which Won Where/What plots, D square analysis, and PCA (Rosyara, 2014; Dumble et al., 2017; Wright and Laffont, 2018; Yaseen et al., 2018; Mendiburu, 2020). Under AMMI, additive (main) effects were estimated using ANOVA and G × E interaction effects (multiplicative effects) were calculated using principal component analysis. The AMMI model (Yan, 2001) is expressed as follows:

(1) $$Y_{i\,j}=\mu +\delta_{i}+\beta_{j}+\sum_{k=1}^{k}\lambda_{k}\,\beta_{i\,k}+\varepsilon_{i\,j}$$

Means of RILs from each environment were used to construct GGE biplots using the site regression linear bilinear model (Yan and Kang, 2003), as depicted below:

(2) $$Y_{i\,j}=\mu +\beta_{j}+\sum_{k=1}^{k}\lambda_{k}\,\delta_{i\,k}\,\beta_{j\,k}+\varepsilon_{i\,j}$$

*Y*_*ij*_ is the *i*^*th*^ genotype/RIL in the *j*^*th*^ environment, *μ* is the overall mean, *δ_*i*_* is the *i*^*th*^ genotypic effect, β*_*j*_* is the *j*^*th*^ environment effect, *λ_*k*_* is the singular value for the PC axis *k*, *δ_*ik*_* is the genotype/RIL eigenvector value for the PC axis *n*, β*_*jk*_* is the environment eigenvector value for the PC axis k, and *ε_*ij*_* is the residual error assumed to be normally and independently distributed.

Stepwise regression analysis was carried out in SAS (Version 9.3) available at ICAR-Indian Institute of Rice Research, Hyderabad. The regression model in terms of matrix notation is expressed as follows:

(3) Y=X⁢β+e

*Y* is the variable; *X* is the vector of exogenous variables, β is the regression coefficient vector, and *e* is the residual term assumed to be normally distributed with e∼N(0,σ)2.

The *D*^2^ diversity analysis was carried out using “biotools” R package which calculates the distance between a pair of rows using the squared generalized Mahalanobis distance equation, which is expressed as follows:

(4) $$D^{2}={\left(x_{i}-x_{j}\right)}^{\prime }\,\sum^{-1}\left(x_{i}-x_{j}\right)$$

Where *x*_*i*_ and *x*_*j*_ are the elements of the *i*^*th*^ row and *j*^*th*^ column and Σ is the non-singular covariance matrix. Finally PCA was done in “FactoMineR” and R package, which creates the uncorrelated linear combination of new variables using correlated original variables.

### Mapping and QTL Analysis

Genomic regions associated with agronomic traits and grain Zn and Fe content were identified using two sets of RILs, viz., a main set of 190 RILs with a rice microsatellite (RM) or simple sequence repeat (SSR) markers and a subset of 44 contrasting RILs (22 lines with Zn > 24.0 ppm, 22 lines with <24.0 ppm zinc) from the main set were subjected to GBS. Genomic DNA was extracted from the leaf using DNAQuik^TM^ isolation kit (BioServe, Beltsville, MD) according to the manufacturer’s instructions, and DNA was quantified with QUBIT dS DNA HS assay kit (Invitrogen, United States) and on 0.8% agarose gel.

For mapping in the main set, parental polymorphism was surveyed with 250 SSR markers^2^. Based on their clear resolution on agarose gel, 102 polymorphic SSRs were used for mapping (Supplementary Table 2). Amplification of SSRs with different annealing temperatures was performed using PCR (Applied Biosystems, 2720) and EmeraldAmp^®^ GT PCR Master Mix (Takara) as per PCR temperature profile (Balaji et al., 2012) (Supplementary Table 3). The amplified products were separated on 3% agarose gel and documented using Alpha Imager 1220 (Alpha Innotech, United States).

Genomic DNA of the subset of 44 contrasting RILs along with two parent genotypes was subjected to whole-genome sequencing (WGS) using Illumina Nextseq 500^TM^. The paired reads of size ∼150 bp were aligned to *Oryza sativa* L. cv. Nipponbare as reference genome using bowtie2 version 2.2.2.6 (Langmead and Salzberg, 2012). The aligned samples and the reference genome sequences were used for variant calling using the SAMtools program (Li et al., 2009). SNP variants from 46 samples (44 RILs and two parents) were annotated based on rice gene model version 7.0, using in-house pipelines (Bioserve Biotechnologies India Private Limited, India). Only SNPs with MAF 0.05 and >70% call rate were considered for analyses.

The linkage map of 190 RILs with 102 SSR markers and 44 RILs with 1305 high-quality SNP markers was constructed using IciMapping v4.2^3^ (Meng et al., 2015). The distribution of SSR- and GBS-based SNP markers varied across chromosomes. The number of SSR markers per chromosome ranged from 7 (chromosome 2, 10 and 11) to 12 (chromosome 6). The total length of the linkage map is 4067.4 cM which ranged from 179.2 (chromosome 2) to 1202.1 cM (chromosome 10) with a mean of 338.95 cM. The number of SNPs per chromosome ranged from 62 (chromosomes 5) to 188 SNPs (chromosome 1). The length of the linkage map ranged from 477.6 (chromosome 5) to 1227.58 (chromosome 2) cM (Supplementary Table 4). The linkage map was created by using the Kosambi function (Kosambi, 1944). QTLs were identified using BLUEs of each RIL with SSRs and SNPs QTLs. The permutation method was used to obtain an empirical threshold for claiming QTLs based on 1000 runs of randomly shuffling the trait values at the 95% confidence level using the BIP function. Epistatic interactions with the logarithm of odds (LOD) threshold value at 5.0 were analyzed to decipher QTL × Environment Interaction using the MET function in IciMapping 4.2. QTLs and were visualized using MapChart v.2.3 (Voorrips, 2002).

### Comparison of Identified QTLs of the Present Study With the Reported QTLs

The positions of the associated SSR and SNP markers identified in the present study were compared to the genomic positions of the markers from the reported QTLs for grain Fe and Zn to study the co-localization. The positions of flanking markers of genomic regions associated with QTLs were retrieved from https://blast.ncbi.nlm.nih.gov/Blast.cgi and analyzed for the putative candidate genes.

### Candidate Gene Analysis

The physical position of each identified QTL was determined by the position of the flanking SSR and SNP markers. The genes physically located within or near the marker interval of the QTL were considered as candidate genes for analyses. Annotation of the genes with functions related to agronomic traits, metal transport, and homeostasis was compiled, and the physical positions of annotated genes were determined using the RAP DB Genome Browser^4^ (Sakai et al., 2013) and Q-TARO (QTL Annotation Rice Online) database^5^ (Yonemaru et al., 2010). Genes annotated as retrotransposons were excluded from the analysis. Genes were functionally characterized into various categories using WEGO (Ye et al., 2006). Networks of the major QTL was created using the Knetminer program^6^. The molecular functional pathways and temporal and spatial expression of the identified candidate genes were studied using RiceXPro version 3.0^7^.

## Results

A wide and continuous variation was observed among 190 RILs for the 10 traits of the study, viz., Zn in polished rice (ZPR), Zn in brown rice (ZBR), Fe in polished rice (IPR), Fe in brown rice (IBR), single plant yield (SPY), 1000 grain weight (TW), panicle length (PL), number of tillers per plant (NT), plant height (PH), and days to 50% flowering (DFF) within and between environments. Only for Fe in polished (IPR) (Table 1 and Supplementary Figure 1) was there a reduction of mean values SPY, TW, PL, NT, PH, and DFF during E2) in comparison to E1. For grain Fe and Zn content in brown and polished rice, reduction of mean values was observed in E1 wet season in comparison to E2 dry season (Table 1 and Supplementary Figure 2). A similar trend of reduction of mean values was also observed in the subset of 44 RILs. Normal distribution was observed for SPY, DFF, and NT in the subset of 44 RILs selected based on the contrasting values of grain Zn, with continuous variation shown in Supplementary Figure 3. Fifty RILs with >28 ppm during dry season were observed. Significant variation was observed for most of the studied traits (Supplementary Tables 5, 6). Six transgressive variants for grain Zn (over the donor parent) with SPY of >20 g were obtained in the present study (Table 2 and Supplementary Figures 3, 4). A single environment data of 15 quality traits/parameters, phytate phosphate, inorganic phosphate, and total phosphate are given in Supplementary Table 7.

**TABLE 1:** Descriptive statistics of 190 RILs (E1 and E2) with BLUE.

Statistic	Year	PH	PL	NT	DFF	SPY	TW	IBR	ZBR	IPR	ZPR
Mean	E1	106.09	26.71	13	102	25.53	23.93	7.31	17.27	1.68	13.1
	PR116	82.8	22.27	13	101	33.2	24.71	5.9	11.8	1.3	9.1
	R. Basmati	135.8	27.33	10.8	91	19.5	21.88	9.7	20.2	1.4	16.9
	E2	102.64	23.97	10	98	20.27	22.97	11.27	24.14	3.2	20.36
	PR116	80.6	22	12.4	94	26.53	25.15	9.5	19.2	2.8	12.5
	R. Basmati	125.6	26	10	85	18.14	19.07	13	27.1	9.4	22.4
	BLUEs	104.8	25.3	11.3	100.0	22.9	23.5	9.3	20.7	2.4	16.8
	PR116_BLUE	82.67	22.14	11.97	97.5	30.04	24.96	8.2	15.8	2.37	11.08
	R. Basmati_BLUE	130.03	26.51	10.73	88	19.22	21.43	11.1	23.45	5.32	19.38
PCV	E1	16.26	10.13	17.16	7.61	20.79	14.91	21.85	17.80	61.89	24.11
	E2	17.61	9.23	12.05	8.87	15.07	13.79	24.76	25.46	55.27	29.14
GCV	E1	16.06	5.56	9.70	3.15	19.44	12.48	19.22	16.93	46.16	22.98
	E2	17.46	6.61	9.23	2.10	13.13	13.31	22.79	23.26	39.97	27.24
Heritability	E1	0.98	0.30	0.32	−0.17	0.87	0.70	0.77	0.90	0.56	0.91
	E2	0.98	0.51	0.59	−0.06	0.76	0.93	0.85	0.83	0.52	0.87
SEM	E1	2.56	0.33	0.26	0.57	0.71	0.45	0.24	0.57	0.13	0.57
	E2	2.43	0.27	0.15	0.71	0.46	0.45	0.44	1.06	0.28	1.04
	BLUEs	1.3	0.1	0.1	0.3	0.3	0.2	0.1	0.3	0.1	0.2
Skewness	E1	0.69	−0.48	1.11	−0.08	0.34	0.54	0.81	0.48	0.36	0.4
	E2	0.43	1.02	0.25	0.12	1.37	0.47	−0.59	−0.15	1.25	0.11
	BLUEs	0.2	0.3	0.4	0.4	0.4	0.7	−0.2	−0.1	0.6	0.1
Kurtosis	E1	0.53	−0.49	1.46	1.09	−0.65	0.85	0.37	−0.36	−0.05	−0.62
	E2	−0.44	4.42	0.56	0.54	1.56	0.67	−0.57	−1.1	1.38	−0.79
	BLUEs	−0.3	0.8	1.2	2.0	−0.3	1.2	0.1	0.0	0.1	0.2
Min and max	E1	62.07	19.19	8	91	12.83	17.49	4.7	11.1	0.19	6.2
		158.9	30.7	18	114	33.07	34.43	15.8	27.33	6.2	23.8
	E2	59.52	19.57	7	84	13.83	15.56	4.9	10.4	0.8	7.6
		151.13	31.33	14	116	29.64	33.43	16.5	38.2	9.3	35.1
	BLUEs	60.6	21.2	8.2	88.0	14.9	16.8	4.8	11.3	0.8	7.6
		150.8	31.0	15.3	115.0	32.4	33.9	13.9	29.7	5.3	27.2
CV (%)	E1	16.74	8.41	13.98	3.88	19.53	12.77	22.01	22.15	53.13	28.82
	E2	16.73	7.86	10.55	5.07	15.6	13.36	27.06	30.05	54.87	34.47
	BLUEs	2.5	0.2	0.2	0.6	0.5	0.4	0.2	0.5	0.1	0.5

*E1, environment 1; E2, environment 2; BLUE, best linear unbiased estimates; PCV, phenotypic coefficient of variation; GCV, genotypic coefficient of variation; SEM, standard error of the mean; min and max, minimum and maximum; CV (%), confidence level percentage; ZPR, zinc content in polished rice (ppm); ZBR, zinc content in brown rice (ppm); IPR, iron content in polished rice (ppm); IBR, iron content in brown rice (ppm); SPY, single plant yield (g); TW, test weight (g); PH, plant height (cm); PL, panicle length (cm); NT, number of tillers per plant; DFF, days to fifty percent flowering (days).*

**TABLE 2:** Descriptive statistics of 44 RILs (E1, E2, E3, and E4) with BLUE.

Statistic	Year	PH	PL	NT	DFF	SPY	TW	IBR	ZBR	IPR	ZPR
Mean	E1	105.95	26.79	12.76	101.9	25.07	23.93	7.41	17.65	1.68	13.52
	PR116	84.5	22.04	11.2	101	34.8	24.66	5.9	12.4	1.33	9.67
	R. Basmati	135	27.66	10.73	91	21.4	23.63	9.7	19.8	1.37	16.37
	E2	100.6	23.89	9.99	97.6	20.35	23.03	11.24	24.25	3.52	20.59
	PR116	80.83	22.23	12.73	94	25.28	25.25	10.5	19.2	3.4	12.5
	R. Basmati	125.07	25.37	10.73	85	17.04	19.23	12.5	27.1	9.27	22.4
	E3	110.32	26.05	10.87	102.67	26.35	23.81	9.79	16.58	2.54	12.79
	PR116	83.5	23.3	12	103.67	33.1	24.5	8	10.47	2.3	8.93
	R. Basmati	137.15	28.79	11.67	98.67	20.13	22.19	10.2	20.37	7.33	16.63
	E4	105.4	23.62	10.03	99.91	24.13	22.63	10.27	18.19	3	14.97
	PR116	81.3	21.5	10	92	23.53	24.05	11.43	16.17	1.47	16.4
	R. Basmati	121.7	25.3	8	89	15.32	20.13	13.07	26	8.13	21.63
	PR116_BLUE	82.53	22.27	11.98	98.75	29.18	24.62	8.96	14.56	2.13	11.88
	R. Basmati_BLUE	129.73	26.78	11.04	90.92	18.47	21.29	11.37	23.32	6.53	19.26
	BLUE	105.5	25.1	11.2	100.8	24.0	23.4	9.6	19.1	2.6	15.4
SEM	E1	2.56	0.33	0.26	0.57	0.71	0.45	0.24	0.57	0.13	0.57
	E2	2.43	0.27	0.15	0.71	0.46	0.45	0.44	1.06	0.28	1.04
	E3	2.53	0.44	0.19	0.9	0.76	0.38	0.27	0.49	0.21	0.49
	E4	2.05	0.48	0.21	0.96	0.58	0.33	0.32	0.5	0.25	0.53
	SEM_BLUE	2.2	0.2	0.1	0.6	0.5	0.4	0.3	0.5	0.1	0.5
Skewness	E1	0.69	−0.48	1.11	−0.08	0.34	0.54	0.81	0.48	0.36	0.4
	E2	0.43	1.02	0.25	0.12	1.37	0.47	−0.59	−0.15	1.25	0.11
	E3	0.64	−0.27	−0.03	0.4	0.52	−0.02	0.32	0.19	2.07	0.33
	E4	0.02	0.33	0.59	0.34	−0.03	0.45	−0.1	−0.17	3.17	−0.08
	Skewness_BLUE	0.5	0.5	0.4	0.0	0.7	0.3	−0.1	0.0	1.6	0.1
Kurtosis	E1	0.53	−0.49	1.46	1.09	−0.65	0.85	0.37	−0.36	−0.05	−0.62
	E2	−0.44	4.42	0.56	0.54	1.56	0.67	−0.57	−1.1	1.38	−0.79
	E3	0.57	−0.77	−0.4	−0.21	−0.7	0.16	−0.38	0.03	4.71	−0.12
	E4	−0.67	−0.43	−0.66	−0.76	−0.87	0.32	−0.15	0.55	12.83	−0.13
	Kurtosis_BLUE	0.2	0.1	1.2	0.1	0.1	1.0	−0.7	−0.9	4.9	−1.1
Min and max	E1	69.27	21.99	9.6	91	16.38	17.64	4.7	11.4	0.03	7.4
		158.9	30.7	18.3	113	33.87	32.02	11.8	27.33	3.87	23.2
	E2	65.7	19.63	7.73	85	15.91	15.78	5.2	10.4	1	7.6
		139.5	31.33	12.73	109	29.41	31.61	16.5	38.2	9.27	34.8
	E3	80.2	20.32	8.33	91.33	18.3	17.1	6.27	9.7	0.97	6.53
		163.1	32.16	14	118.33	37.19	30.32	14.3	24.37	7.5	20.47
	E4	72.9	16.3	8	89	15.32	17.12	5.3	9.33	1.4	6.37
		130.4	30.63	13	113.33	30.61	28.73	15.27	26	11.37	22.33
	Min_BLUE	73.6	22.3	9.1	90.9	18.5	17.2	6.2	12.3	1.2	8.9
	Max_BLUE	147.8	29.4	13.9	110.6	31.4	30.0	12.8	26.9	6.5	23.4
CV (%)	E1	16.74	8.41	13.98	3.88	19.53	12.77	22.01	22.15	53.13	28.82
	E2	16.73	7.86	10.55	5.07	15.6	13.36	27.06	30.05	54.87	34.47
	E3	15.87	11.6	12.13	6.09	19.77	11.06	19.08	20.35	56.01	26.19
	E4	13.46	14.05	14.38	6.66	16.53	10.13	21.63	18.9	57.36	24.07
	CV (%)_BLUE	4.5	0.5	0.3	1.2	1.0	0.7	0.5	1.1	0.3	1.1

*E1, environment 1; E2, environment 2; E3, environment 3; E4, environment 4; BLUE, best linear unbiased estimates; PCV, phenotypic coefficient of variation; GCV, genotypic coefficient of variation; SEM, standard error of the mean; min and max, minimum and maximum; CV (%), confidence level percentage; ZPR, zinc content in polished rice (ppm); ZBR, zinc content in brown rice (ppm); IPR, iron content in polished rice (ppm); IBR, iron content in brown rice (ppm); SPY, single plant yield (g); TW, test weight (g); PH, plant height (cm); PL, panicle length (cm); NT, number of tillers per plant; DFF, days to fifty percent flowering (days).*

### Correlations

Among 190 RILs with BLUE values of 10 traits, highly significant positive correlations were observed among ZPR, ZBR, IPR, and IBR. Low to moderate significant positive correlations were identified between PH and PL and also between TW and SPY. Moderate negative correlations were found between DFF with ZBR and IBR (Supplementary Tables 8, 9). In the subset of 44 RILs, also significant positive correlations were observed among ZPR, ZBR, IPR, and IBR along with moderate negative correlations for DFF with ZPR, IPR, and ZBR (Supplementary Tables 10, 11). Correlation analyses of quality parameters of a single environment data with grain Zn and Fe showed that IPR has a significant positive correlation with kernel length after cooking (KLAC) (0.58) and elongation ratio (ER) (0.58). IBR also showed a moderate positive correlation with ER (0.40) and with alkali spreading value (ASV) (0.40). Low positive correlations of inorganic phosphate with kernel breadth (KB) (0.47) and negatives correlation with head rice recover (HRR) (0.55) were also observed (Supplementary Table 12).

### D Square Analysis

The dendrogram of 190 RILs with 10 traits has shown 28 clusters, among which the first cluster is the largest group having 109 members, followed by cluster 5 and so on. Cluster 3 with seven RILs has the highest mean grain Zn (27.84 ppm) (Supplementary Table 13 and Supplementary Figure 5). For the subset of 44 RILs, there were 13 clusters among which cluster one formed the largest group with 25 members followed by the third cluster and so on. Cluster 3 with four RILs showed the highest mean grain Zn (24.22 ppm) (Supplementary Table 14 and Supplementary Figure 5).

### PCA

The PCA (principal component analysis) was performed for 190 RILs and for subset of 44 RILs. For 190 RILs, the first four PCs were found to be most important as their eigenvalues are more than or equal to one and together they explain around 68% of variability. The first four PCs contributed 28%, 15%, 14%, and 10%, respectively. The first PC showed a positive association with original variables, viz., PH, NT, TW, IBR, ZBR, IPR, and ZPR, and a negative association with PL, DFF, and SPY. Four variables, viz., IBR, ZBR, IPR, and ZPR, contributed 98% of variation in the first PC. The second PC showed a positive association with NT, DFF, IBR, IPR, and ZPR and showed a negative association with PH, PL, NT, DFF, SPY, TW, and ZBR. The traits PH, PL, NT, DFF, and SPY, together explained 99% of variability in the second PC. The third PC depicted a positive association with PH, PL, NT, DFF, IPR, and ZPR and a negative association with SPY, TW, IBR, and ZBR. The traits PH, PL, NT, DFF, SPY, and TW contributed a maximum variation around 95% in the third PC. The 4^*th*^ PC showed a smaller positive association with IBR, IPR, and ZPR and has a negative association with the rest of the traits. The traits NT and SPY contributed a maximum variation around 94% in the fourth PC (Supplementary Tables 15, 16 and Supplementary Figure 6).

According to the PCA of 44 subset RILs, the first four PCs with eigenvalues of more than or equal to one explained around 73% of variation. Individually, the first four PCs contributed 36%, 15%, 12%, and 10% of variations, respectively. The first PC showed a positive association with PH, PL, NT, IBR, ZBR, IPR, and ZPR and showed a negative association with DFF, SPY, and TW. The traits IBR, ZBR, IPR, and ZPR together contributed 87% of variability in the first PC. The second PC has shown a positive association with NT, SPY, TW, IBR, ZBR, and ZPR and showed a negative association with PH, PL, DFF, and IPR. The traits PH, PL, NT, DFF, SPY, and TW contributed 93% of variation in the second PC. The third PC showed a positive association with PL, NT, SPY, and IPR and showed a negative association with PH, DFF, TW, IBR, ZBR, and ZPR. The traits NT, DFF, SPY, and TW together contributed 93% of variation in the third PC. The fourth PC has shown a positive association with PH, PL, NT, DFF, IPR, and ZPR and a negative association with SPY, TW, IBR, and ZBR. The traits PH, PL, NT, DFF, and SPY together contributed around 90% of variation in the fourth PC (Supplementary Tables 17, 18 and Supplementary Figure 6).

### Stepwise Regression Analysis of 190 RILs for Grain Zn and Fe and Yield

The stepwise regression analysis was carried out to identify the factors influencing ZPR, ZBR, IPR, and IBR content in this study, and all the 10 variables were used in regression analysis. Stepwise regression analysis for ZPR was carried out over the remaining nine independent variables, and the model retained only two significant variables, namely, ZBR (77%) and IPR (2%), which explained 79% variation in the model, and the rest of the variations may be explained by the variables which were not considered in this study. The stepwise regression Eq. (5) for the ZPR model is expressed as follows;

(5) $$Z\,\hat{P\,R_{190}}=-0.75+0.77\,Z\,B\,R+0.65\,I\,P\,R$$

The regression coefficients depicts that, for every 1 ppm increase in ZBR, there was a 0.77-ppm increase in ZPR and for every ppm unit increase IPR, there will be a 0.65-ppm increase in ZPR.

The ZBR 190 stepwise regression model retains three variables, viz., ZPR (77%), IPR (0.5%), and IBR (5%), and together they contributed 82% of R^2^. The regression equation for ZBR 190 is expressed in Eq. (6):

(6) $$Z\,\hat{B\,R_{190}}=3.09+0.63\,I\,B\,R-0.29\,I\,P\,R+0.74\,Z\,P\,R$$

As per the regression coefficients, for every 1 ppm of ZPR increase, there was an increase of 0.74 ppm of ZBR and for every 1 ppm of IBR increase, there was an increase of 0.63 ppm of ZBR, but for every 1 ppm increase of IPR, ZBR was decreased by 0.29 ppm.

The IPR 190 regression model was influenced by TW (3%), IBR (4%), ZBR (1%), and ZPR (29%); altogether, they explained 37% of variation in the model and the remaining variations may be explained by other factors which were not included in the present study. Regression Eq. (7) for IPR is expressed as:

(7) $$I\,\hat{P\,R_{190}}=0.86-0.05\,T\,W+0.18\,I\,B\,R-0.06\,Z\,B\,R+0.13\,Z\,P\,R$$

IBR and ZPR have a positive effect on the status of IPR; for every 1 ppm of IBR and ZPR increase, there was an increase in IPR of 0.18 ppm with IBR and 0.13 ppm with ZPR, and ZBR has a negative influence on IPR with every 1 g of TW and 1 ppm of ZBR increase; IPR was decreased by 0.05 ppm with TW and 0.06 ppm with ZBR.

The stepwise regression model for IBR (Eq. 8) depicts that IBR was influenced by TW (1%), ZBR (54%), and IPR (3%).

(8) $$I\,\hat{B\,R_{190}}=0.64+0.06\,T\,W+0.30\,Z\,B\,R+0.44\,I\,P\,R$$

Regression coefficients of the IBR 190 model predict that for a 1-g increase of TW, a 0.06-ppm increase of IBR was noted. Similarly, with a 1-ppm increase of ZBR and IPR, 0.30 ppm and 0.44 ppm of IBR increase were observed.

In the SPY 190 model (Eq. 9), mainly two variables, viz., PH (1%) and TW (4%), were entered in the model, and it clearly showed that other large amounts of variation may be explained by other factors which were not included in this study.

(9) $$S\,\hat{P\,Y_{190}}=14.50+0.02\,P\,H+0.25\,T\,W$$

For every 1 cm of PH and 1 g of TW increase, SPY increased by 0.02 g with PH and 0.25 g with TW.

### Stepwise Regression Analysis for 44 RILs for Grain Zn and Fe and Yield

The four environments’ phenotype data of 10 traits of the subset of 44 contrasting RILs (22 lines with Zn > 24.0 ppm, 22 lines with <24.0 ppm zinc) was also analyzed for regression (Supplementary Table 19). For the subset, ZPR was found to be influenced by SPY (1%) and ZBR (93%). Regression Eq. (10) for ZPR is expressed as follows:

(10) $$Z\,\hat{P\,R_{44}}=-5.39+0.08\,S\,P\,Y+0.99\,Z\,B\,R$$

Regression coefficients explained that for every 1-ppm increase in ZBR, there was an increase by 0.99 ppm of ZPR. SPY showed a positive effect on ZPR with an increase of 1 g of SPY, and there was an increase by 0.08 ppm of ZPR.

The ZBR was influenced by PL (0.5%), SPY (1%), TW (0.5%), IBR (1%), and ZPR (93%). Regression Eq. (11) for ZBR is expressed as:

(11) $$Z\,\hat{B\,R_{44}}=12.63-0.17\,P\,L-0.11\,S\,P\,Y-0.09\,T\,W+0.271\,B\,R+0.83\,Z\,P\,R$$

Regression coefficients in the ZBR 44 model depicted that for every 1-ppm increase of IBR and ZPR, there was an increase by 0.27 ppm of ZBR with IBR and 0.83 ppm of ZBR with ZPR. PL, SPY, and TW showed a negative impact on ZBR, with for every 1-cm increase of PL; 1 g of SPY and TW increased, and ZBR decreased by 0.17 ppm with PL, 0.11 ppm with SPY, and 0.09 ppm with TW.

The IPR was influenced by PL (4%), DFF (6%), TW (7%), and ZPR (37%). Regression Eq. (12) for IPR was:

(12) $$I\,\hat{P\,R_{44}}=6.74+0.17\,P\,L-0.09\,D\,F\,F-0.08\,T\,W+0.15\,Z\,P\,R$$

PL and ZPR have a positive effect on IPR; for every 1-cm increase of PL and 1-ppm increase of ZPR, IPR increased by 0.17 ppm with PL and 0.15 ppm with ZPR. DFF and TW showed a negative effect on IPR, with every 1-day increase of DFF and 1-g increase of TW; there was a decrease of IPR by 0.09 ppm with DFF and 0.08 ppm with TW.

The IBR was influenced by ZPR (72%) as the only variable that entered in the IBR stepwise regression model (Eq. 13):

(13) $$I\,\hat{B\,R_{44}}=3.59+0.39\,Z\,P\,R$$

ZPR has a positive effect on IBR, and for every 1-ppm increase of ZPR, IBR increased by 0.39 ppm.

In the subset of 44 RILs, SPY was influenced only by two factors, viz., ZBR (5%) and ZPR (7%). Regression Eq. (14) for SPY is expressed as:

(14) $$S\,\hat{P\,Y_{44}}=30.85-1.08\,Z\,B\,R+0.90\,Z\,P\,R$$

ZBR has a negative effect and ZPR a positive effect on SPY; every 1-ppm increase of ZBR, SPY decreased by 1.08 ppm, and every 1-ppm increase of SPY, ZPR increased by 0.90 ppm.

### Stability of 44 RILs Across the Environments

Combined ANOVA of 44 RILs across four environments indicated significant variance for RILs as well as for genotype × environment effect for the traits of study.

### Zinc Content in Polished Rice (ZPR)

**FIGURE 1:**
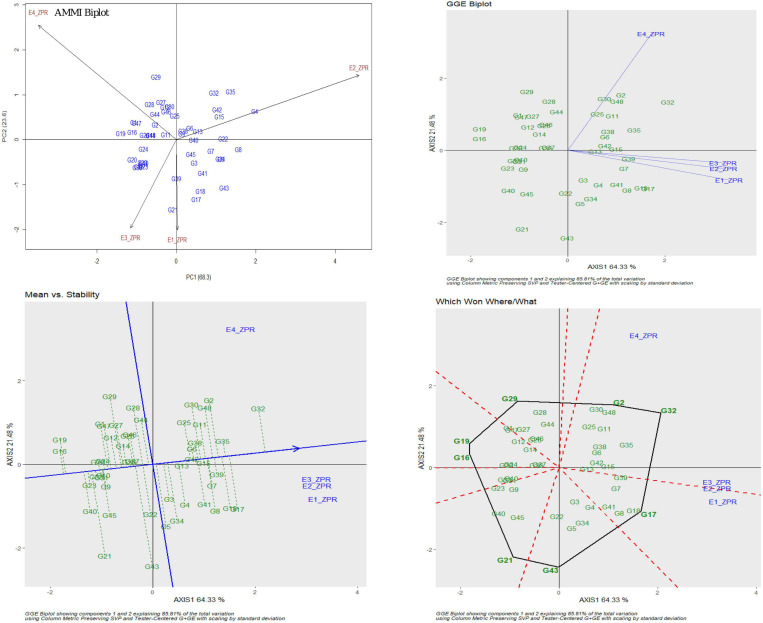
AMMI and GGE biplot for ZPR across four environments: AMMI biplot, GGE biplot, mean vs. stability and Which Won Where/What.

For ZPR, 8.06% genotypic effect, 89.37% environment effect, and 1.90% genotype × environment effect was observed. According to AMMI analysis, PC1 contributed 68.3% variability, PC2 contributed 23.6% variability, and PC3 contributed 8.1% variability (Table 3). The AMMI biplot showed 91.9% of goodness of fit with 68.3% of PC1 and 23.6% of PC2 contribution from IPCA (interaction principal components axes) 1 and 2, respectively, and with the highest mean values, E2 was found to be a favorable season. G15 was closer to the origin and relatively stable RILs, and G42 farther from the IPCA line was found to be the specific adapter across the seasons. G32 was found to be the best in E3 and E4, whereas G7 in E1 and G39 were found the best in E1 and E2 and G13 was found the best in E1, E2, and E3. Based on the mean vs. stability, G15 and G13 were more stable. As per the Which Won Where/What graph, G17 won in E1, G32 and G2 in E4, and G32 in E3 and E4 (Figure 1).

**TABLE 3:** AMMI analysis of variance for the yield and mineral micronutrients traits in the subset of 44 RILs.

Traits	SV	ENV	REP (ENV)	GEN	ENV:GEN	Residuals	Total	PC1	PC2	PC3

	df	3	8	47	141	376	575	49	47	45
ZPR	SS	1750.15***	9.41*	157.77***	37.26***	3.8	1958.39	3590.59***	1238.08***	424.99***
	VE (%)	89.37	0.48	8.06	1.9	0.19	100	68.3	23.6	8.1
IPR	SS	87.14***	1.44	16.56***	3.84***	1.16	110.16	265.71***	203.36***	72.82
	VE (%)	79.11	1.31	15.04	3.49	1.06	100	49	37.5	13.4
IBR	SS	388.53***	0.81	33.91***	8.76***	2.09	434.1	549.7***	474.86***	210.35***
	VE (%)	89.5	0.19	7.81	2.02	0.48	100	44.5	38.5	17
ZBR	SS	1717.85***	2.75	155.49***	39.77***	2.96	1918.82	3896.3***	1142.78***	567.9***
	VE (%)	89.53	0.14	8.1	2.07	0.15	100	69.5	20.4	10.1
SPY	SS	977.24***	14.68*	129.87***	35.6***	6.1	1163.49	3155.91***	1014.82***	849.3***
	VE (%)	83.99	1.26	11.16	3.06	0.52	100	62.9	20.2	16.9
TW	SS	54.06	15.25**	71.77***	6.57*	4.83	152.5	462.19***	265.31	199.37
	VE (%)	35.45	10	47.06	4.31	3.17	100	49.9	28.6	21.5
PH	SS	2275.48***	30.56**	2958.53***	119.49***	11.57	5395.63	11926.16***	3654.97***	1266.47***
	VE (%)	42.17	0.57	54.83	2.21	0.21	100	70.8	21.7	7.5
PL	SS	355.66**	32.83***	29.27***	19***	7.52	444.28	1735.62***	563.27*	380.38
	VE (%)	80.05	7.39	6.59	4.28	1.69	100	64.8	21	14.2
NT	SS	241.55**	23.34***	8.58**	5.25	5.13	283.86	314.68	293.08	132.34
	VE (%)	85.1	8.22	3.03	1.85	1.81	100	42.5	39.6	17.9
DFF	SS	738.95**	76.16	204.38**	55.43**	39.74	1114.66	4736.03***	2243.17	836.27
	VE (%)	66.29	6.83	18.34	4.97	3.57	100	60.6	28.7	10.7

***Significant at *P* < 0.0001. **Significant at *P* < 0.01. *Significant at *P* < 0.05; SV, source of variation; PC, principal component; ENV, environment; GEN, genotype; ZPR, zinc content in polished rice (ppm); ZBR, zinc content in brown rice (ppm); IPR, iron content in polished rice (ppm); IBR, iron content in brown rice (ppm); SPY, single plant yield (g); TW, test weight (g); PH, plant height (cm); PL, panicle length (cm); NT, number of tillers per plant; DFF, days to fifty percent flowering (days).

### Zinc Content in Brown Rice (ZBR)

A total 8.10% of genotypic effect, 89.53% of environment effect, and 2.07% of genotype × environment effect were observed for ZBR. PC1 contributed 69.5% variability, PC2 contributed 20.4% variability, and PC3 contributed 10.1% variability to the AMMI biplot with 89.9% of goodness of fit and 69.5% of PC1 and 20.4% PC2 contribution from IPCA (Table 3). E2 was found to be a favorable season with the highest mean values of RILs. G11 was closer to the origin, and thus relatively stable RIL and G6 were farther from the IPCA line and were found to be specific adapters. G2 was in E4; G7, G18, and G42 were in E1 and E2; G38 was in E2 and E3; and G32 was found in E3 and E4, whereas G17 was found to be the best in E1. Based on the mean vs. stability, G11 was found as stable. According to the Which Won Where/What graph, G17 won in E1, and RILs G2 and G32 won in E3 and E4. E3 was found as the representative environment as it falls on the Mean–Environment Axis (Supplementary Figure 7).

### Iron Content in Polished Rice (IPR)

For IPR, the genotypic effect was 15.04%, the environment effect was 79.11%, and the genotype × environment effect was 3.49%. AMMI analysis has shown PC1 with 49.0% variability, PC2 with 37.5% variability, and PC3 with 13.4% variability (Table 3). The AMMI biplot showed 86.5% of goodness of fit with 49.0% PC1 and 37.5% PC2 contribution from IPCA. E4 has the highest mean value. G15 was found near the origin, and G3 was found to be the best in E1 and E3, whereas G2 and G48 were found to be the best in E4. E4 was found as the representative environment as it falls on the Mean–Environment Axis. Based on the mean vs. stability, G15 was observed to be a stable RIL. With reference to the Which Won Where/What graph, G39 won in E1, G3 won in E1 and E3, and G2 and G48 won in E4 (Supplementary Figure 8).

### Iron Content in Brown Rice (IBR)

A total of genotypic effect of 7.81%, environment effect of 89.50%, and genotype × environment effect of 2.02% were observed. For AMMI analysis, PC1 contributed 44.5% variability, PC2 contributed 38.5% variability, and PC3 contributed 17% variability (Table 3). The AMMI biplot showed 83.0% of goodness of fit with 44.5% of PC1 and 38.5% of PC2 contribution from IPCA 1 and 2, respectively. G42 RIL was closer to the origin and was considered as a relatively stable RIL, and G7 and G11 farther from the IPCA line were found to be the specific adapters. All four seasons showed almost equal discrimination power, whereas E2 was identified as the representative environment, as it falls on the Mean–Environment Axis. G42 and G46 were found near the origin and were considered as stable RILs. G32 was identified to be the best in E2 and E4; G18 RIL was found to be the best in E1 and G7 performed best in E2. Based on the mean vs. stability, G42 was found to be more stable. With reference to the Which Won Where/What graph, G18 and G3 won in E1, and G32 won in E2 and E4 (Supplementary Figure 9).

### Single Plant Yield (SPY)

**FIGURE 2:**
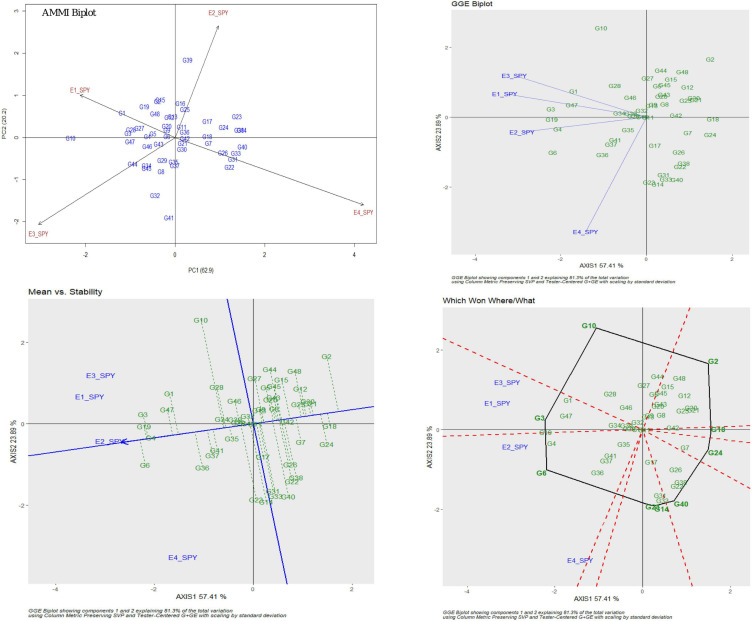
AMMI and GGE biplot for SPY across four environments: AMMI biplot, GGE biplot, Which Won Where/What and mean vs. stability.

For SPY, the genotypic effect was 11.16%, the environment effect was 83.99%, and the genotype × environment effect was 3.06%. AMMI analysis has shown 62.9% of PC1, 20.2% of PC2, and 16.9% of PC3 contribution to variability. The AMMI biplot showed 83.1% of goodness of fit with 62.9% of PC1 and 20.2% of PC2 contribution from IPCA 1 and 2, respectively (Table 3). The highest mean values were observed for most of the RILs in E2. The four seasons showed almost equal discrimination power. G16, G29, and G11 were found near the origin and hence were considered as less interactive RIL and thus considered to be less interactive and relatively stable. G6 performed best in E2 and E4; G4 performed best in E2; G3 and G19 performed best in E1 and E2; G1 performed best in E3; and G47 performed best in E1. Based on the mean vs. stability, G29 was considered to be more stable RIL. According to the Which Won Where/What graph), G6 won in E2 and E4, and G3 won in E1 and E2 (Figure 2).

### Test Weight (TW)

A total of 47.16% genotypic effect, 35.45% environment effect, and 4.31% genotype × environment effect were observed. The AMMI biplot has shown 78.5% of goodness of fit with 49.9% of PC1 and 28.6% of PC2 contribution from IPCA (Table 3). AMMI analysis presented PC1 with 49.9% contribution, PC2 with 28.6% contribution, and PC3 with 21.5% contribution toward variability. All seasons showed almost equal discrimination power, and the highest mean values were observed for most of the RILs during E1. G28 RIL was closer to the origin and hence considered as a relatively stable RIL, and G14 farther from the IPCA line was found to be the specific adapter. G26 was found near the origin and found to be the lesser interactive RIL. G46 and G22 were found to be the best in E1 and E2; G6 and G41 were found to be the best in E3 and E4; and G19 RIL was found to be the best in E1 and E3. Based on the mean vs. stability G28 was found as stable RIL), according to the Which Won Where/What graph, G22 won in E1 and E2, G19 won in E1, E3, and E4; and G6 and G41 won in E3 and E4 (Supplementary Figure 10).

### Plant Height (PH)

For plant height, across all the environments, the genotypic effect was 54.83%, the environment effect was 42.17%, and the genotype × environment (G × E) effect was 2.21%. AMMI analysis has shown PC1 contributing 70.8%, PC2 contributing 21.7%, and PC3 contributing 7.5% toward variability (Table 3). The AMMI biplot showed 92.5% of goodness of fit with 70.8% of PC1 and 21.7% of PC2 contribution from IPCA. The highest mean values were observed in E2. All the seasons showed almost equal discrimination power. G25 was found near the origin and was considered to be the less interactive RIL. G14 and G29 farther from the IPCA line were found to be the specific adapters. G9 was found to be the best in E2, G16 was found to be the best in E3, and G2 was found to be the best in E1 and E3. Based on the mean vs. stability, G9 and G14 were found more stable among the seasons. From the Which Won Where/What graph, G16 won in E3, and G10 and G18 won in E4 (Supplementary Figure 11).

### Panicle Length (PL)

For PL, the genotypic effect was 6.59%, the environment effect 80.05%, and the genotype × environment effect 4.28%. AMMI analysis has shown PC1 contribution of 64.8%, PC2 contribution of 21.0%, and PC3 contribution of 14.2% to variability. The AMMI biplot has also shown 85.8% of PC1 and 21.0% of PC2 goodness of fit with 64.8% contribution from IPCA (Table 3). E3 has the highest mean values, and thus found to be favorable for expression in most of the RILs. All seasons showed almost equal discrimination power. G3 was found near the origin and hence considered as less interactive RIL. G18 was found to be the best in E4; G34 was found to be the best in E1; G30 was found to be the best in E3; and G16 was found to be the best in E1 and E2. Based on the mean vs. stability, G15 RIL was found to be more stable among the seasons. According to the Which Won Where/What graph, G30 won in E3, G34 won in E1, G18 won in E4, and G16 won in E1 and E2 (Supplementary Figure 12).

### Number of Tillers per Plant (NT)

A total of 3.03% genotypic effect, 85.10% of environment effect, and 1.85% genotype × environment effect were observed. The AMMI biplot showed 82.1% of goodness of fit with almost 42.5% of PC1 and 39.6% of PC2 contribution from the IPCA line. The AMMI analysis has shown 42.5% contribution from PC1, 39.6% contribution from PC2, and 17.9% contribution from PC3 toward variability (Table 3). E1 found to be a more favorable season for the expression of trait with the highest mean values. Four seasons showed almost equal discrimination power, whereas E2 was found as the representative environment, as it falls on the Mean–Environment Axis. G17 was closer to the IPCA origin and hence considered to be a relatively stable RIL across seasons. G15 was found near the origin and noted as the less interactive RIL. G11 was found to be the best in E1 and E2, whereas G8 was found to be the best in E1 and E3. Based on the mean vs. stability, G17 was found to be more stable. As per Which Won Where/What, G8 won in E1 and E3, G11 won in E1 and E2, and G14 won in E4 (Supplementary Figure 13).

### Days to Fifty Percent Flowering (DFF)

For DFF, 18.34% of genotypic effect, 66.29% of environment effect, and 4.97% of genotype × environment effect were observed. PC1 contributed 60.6% variability, PC2 contributed 28.7% variability, and PC3 contributed 10.7% variability as noted from the AMMI analysis (Table 3). The AMMI biplot showed 89.3% of PC1 and 28.7% of PC2 goodness of fit with 60.6% contribution from the IPCA line. With the highest mean values, E2 was found to be a favorable season. All four environments showed almost equal discrimination power. G46 was found near and closer to the origin and found to be stable. G18 farther from the IPCA line was found to be a specific adapter. G15 was found to be the best in E1 and E2, G21 was found to be the best in E1 and E3, and G26 was found to be the best in E2 and E4. According to the mean vs. stability graphs, G46 was found to be more stable RIL across the seasons. According to the Which Won Where/What graph, G21 won in E1 and E3, G15 won in E1 and E2, and G35 won in E4 (Supplementary Figure 14).

### Identification of QTL

#### SSR Based

**FIGURE 3:**
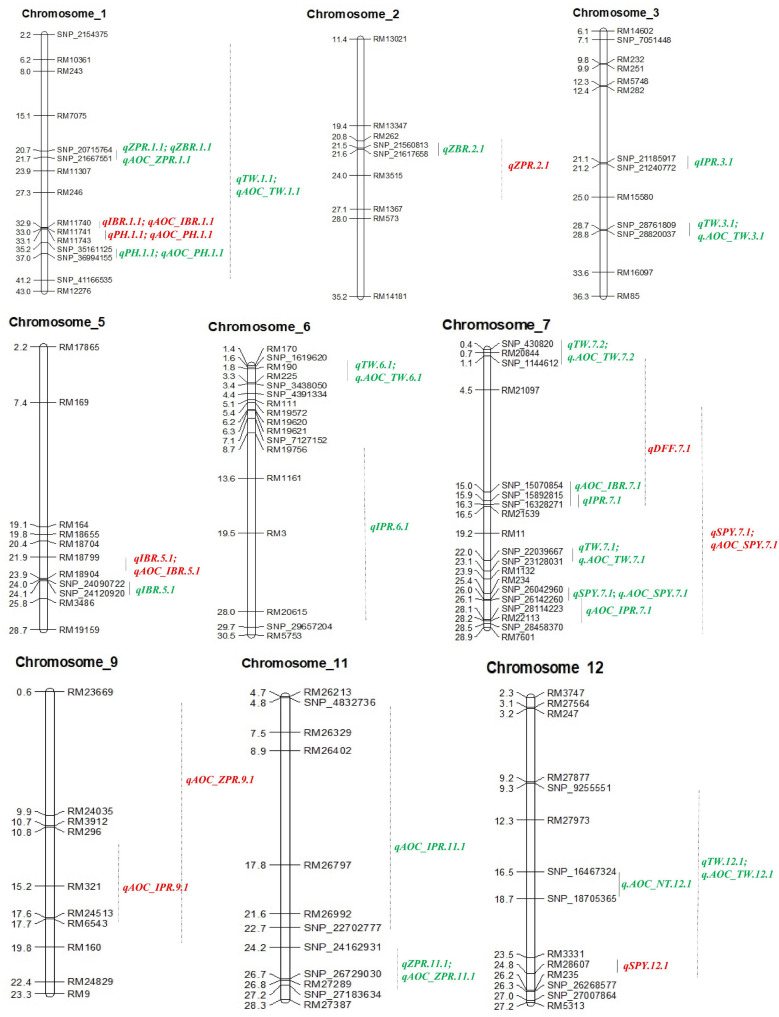
QTL identified with SSRs and SSR_AOC (red), SNPs and SNP_AOC (green) in RIL of PR116/Ranbir Basmati using IciMapping v4.2. AOC, advanced over check; ZPR, zinc content in polished rice (ppm); ZBR, zinc content in brown rice (ppm); IPR, iron content in polished rice (ppm); IBR, iron content in brown rice (ppm); SPY, single plant yield (g); TW, test weight (g); PH, plant height (cm); NT, number of tillers per plant.

Out of 102 polymorphic SSRs, per chromosome the number ranged from 7 (chromosome 10 and 11) to 12 (chromosome 6). A linkage map of 4067.4 cM was constructed with the size of each chromosome ranging from 179.2 to 1202.1 cM with a mean of 338.95 cM (Table 4 and Figure 3). A total of 13 QTLs detected in 190 RILs with two seasons of BLUEs including advantage over check values (AOC). Out of 13 QTLs, nine QTLs were from the donor parent (Ranbir Basmati) and only two QTLs for SPY and DFF were from the recipient parent (PR116). Four QTLs for ZPR, IBR, SPY, and PH overlapped with QTLs for AOC of the same traits. Only one QTL with a moderate effect was observed for ZPR (*qZPR.2.1*: PV 11.3%) and IBR (*qIBR.5.1*: PV 10.1%) on chromosomes 2 and 5. The remaining QTLs were identified to be with low PV % (Table 4).

**TABLE 4:** Identification of SSR based QTL in 190 RILs with BLUE and AOC_BLUE.

S. no.	Trait	QTL	Chr	Position (cM)	Marker interval	LOD	PV (%)	Add	Allele	Region reported
1	ZPR	*qZPR.2.1*	2	123	RM1367–RM262	5.25	11.3	−0.56	Parent 2	Raza et al. (2019)
2	AOC_ZPR	*qAOC_ZPR.9.1*	9	92	RM160–RM23669	2.65	6.23	−5.27	Parent 2	Raza et al. (2019); Jeong et al. (2019); Calayugan et al. (2020)
3	AOC_IPR	*qAOC_IPR.9.1*	9	140	RM6543–RM296	3.26	7.52	−11.46	Parent 2	Kumar et al. (2019); Raza et al. (2019)
4	IBR	*qIBR.1.1*	1	151	RM11741–RM11740	2.8	6.48	−0.44	Parent 2	Swamy et al. (2018b); Dixit et al. (2019); Jeong et al. (2019)
5	AOC_IBR	*qAOC_IBR.1.1*	1	152	RM11741–RM11740	2.67	6.26	−3.87	Parent 2	Swamy et al. (2018b); Dixit et al. (2019); Jeong et al. (2019)
6	IBR	*qIBR.5.1*	5	175	RM18904–RM18799	3.66	10.11	−0.74	Parent 2	Kumar et al. (2019)
7	AOC_IBR	*qAOC_IBR.5.1*	5	175	RM18904–RM18799	4.41	11.74	−7.1	Parent 2	Kumar et al. (2019)
8	SPY	*qSPY.7.1*	7	22	RM7601–RM21097	3.24	1.39	0.99	Parent 1	
9	AOC_SPY	*qAOC_SPY.7.1*	7	22	RM7601–RM21097	3.17	7.4	3.19	Parent 1	
10	SPY	*qSPY.12.1*	12	236	RM28607–RM235	2.58	9.02	2.5	Parent 1	Kumar et al. (2019)
11	PH	*qPH.1.1*	1	146	RM11743–RM11741	3.33	7.66	−5.31	Parent 2	Dixit et al. (2019)
12	AOC_PH	*qAOC_PH.1.1*	1	146	RM11743–RM11741	3.33	7.67	−6.44	Parent 2	Dixit et al. (2019)
13	DFF	*qDFF.7.1*	7	187	RM21539–RM20844	2.78	7.7	1	Parent 1	Calayugan et al. (2020)

*Chr, chromosome; QTL, quantitative trait loci; cM, centimorgans; PV, phenotypic variance explained; LOD, logarithm of the odds; Add, additive effect; AOC, advanced over check; ZPR, zinc content in polished rice (ppm); IPR, iron content in polished rice (ppm); IBR, iron content in brown rice (ppm); SPY, single plant yield (g); PH, plant height (cm); DFF, days to fifty percent flowering (days); Parent 1–Recipient parent (PR116); Parent 2–Donor parent (Ranbir Basmati)*

### Epistatic Interaction Analysis

**FIGURE 4:**
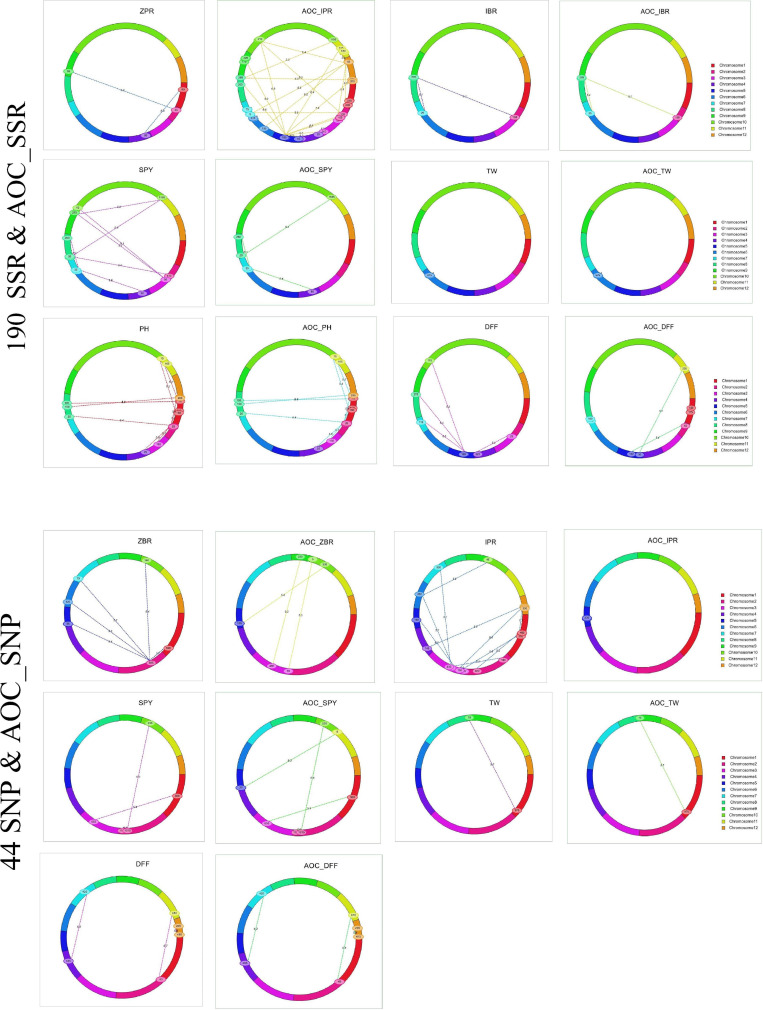
Epistatic interaction of SSR & AOC_SSR and SNP & AOC_SNP in 190 and 44 RILs using IciMapping v4.2.

Out of 75 epistatic interactions identified, only one interaction for ZPR (PV 11.3%) between chromosomes 1 and 4 was observed (Figure 4). For IBR, two digenic interactions between chromosomes 2 (PV > 12.7%) and 8 and chromosomes 7 and 8 (PV > 18.9%) were found. All the remaining interactions found to be with low PV% (<10%) (Supplementary Table 20).

#### GBS Based

A total of 19,626 SNPs were obtained with a maximum number of SNPs (11.82%) in chromosome 1 (2319) and a minimum number of SNPs (5.75%) in chromosome 10 (1129). Out of 19626, 5206 polymorphic SNPs between the parents were considered after removal of monomorphic SNPs. The linkage map was constructed with 1035 SNPs. A maximum number of polymorphic SNPs (13.22%) were found in chromosome 1 (688), and a minimum number of polymorphic SNPs (5.42%) were observed in chromosome 12 (542) (Supplementary Table 4). Genetic maps were constructed from linkage data of RIL population, and QTLs (≥2.5 LOD) were identified using composite interval mapping (CIM) with graphical output using IciM4.2 software (Jansen, 1994; Zeng, 1994).

Thirty SNP QTLs were identified for eight traits with PV ranging from 5 to 37.84% identified based on BLUEs derived from four seasons (Table 5 and Figure 3). A major SNP-QTL for ZPR as *qZPR.1.1* (PV 37.84%) on chromosome 1 and a moderate QTL as *qZPR.11.1* (PV 15.47%) on chromosome 11 were identified. Another major SNP-QTL was also detected for ZBR as *qZBR.1.1* (PV 30.61%) on chromosome 1 along with a moderate QTL as *qZBR.2.1* (PV 19.84%) on chromosome 2. Three SNP-QTLs, viz., *qIPR.3.1* (PV 34.75%), *qIPR.6.1* (PV 15.29%), and *qIPR.7.1* (PV 12.66%), were found on chromosomes 3, 6, and 7 with two additional QTLs for *AOC as qIPR.7.1* (PV 15.62%) on chromosome 7 and *qIPR.11.1* (PV 31.65%) on chromosome 7. For IBR, a major QTL *qIBR.5.1* (PV 33.02%) on chromosome 5 and a moderate QTL *qAOC_IBR.7.1* with PV 22.13% on chromosome 7 were identified. The QTL for grain Zn and Fe traits (ZPR, ZBR, IPR, and IBR) were contributed from the donor parent (Ranbir Basmati). A major QTL *qSPY.7.1* (PV 25.74%) was identified for SPY on chromosome 7 contributed by the recipient parent (PR116). Six QTLs for TW and AOC_TW such as *qTW.1.1*, *qTW.3.1*, *qTW.6.1*, *qTW.7.1*, *qTW.7.2*, and *qTW.12.1* were identified spread over chromosomes 1, 3, 6, 7, and 12 with PV ranging from 4.99 to 36.93%. *qTW.7.1* with the highest PV (36.93%) among identified QTLs for TW was contributed by the recipient parent (PR116). Another QTL, *qPH.1.1* (PV 23.06%), was identified on chromosome 1 with for PH contributed by the donor parent. A major QTL *qAOC_NT.12.1* for AOC_NT (PV 25.16%) on chromosome 12 was identified (Table 5).

**TABLE 5:** Identification of SNP based QTL in 44 RILs with SNP BLUE and AOC_BLUE.

S. no.	Trait	QTL	Chr	Position (cM)	Marker interval	LOD	PV (%)	Add	Allele	Region reported
1	ZPR	*qZPR.1.1*	1	185	SNP_21667551–SNP_20715764	4.85	37.84	−2.27	Parent 2	Anuradha et al. (2012); Dixit et al. (2019)
2	AOC_ZPR	*qAOC_ZPR.1.1*	1	185	SNP_21667551–SNP_20715764	4.85	37.84	−10.35	Parent 2	Anuradha et al. (2012); Dixit et al. (2019)
3	ZPR	*qZPR.11.1*	11	658	SNP_27183634–SNP_24162931	2.53	15.47	−1.8	Parent 2	
4	AOC_ZPR	*qAOC_ZPR.11.1*	11	658	SNP_27183634–SNP_24162931	2.53	15.47	−8.19	Parent 2	
5	ZBR	*qZBR.1.1*	1	185	SNP_21667551–SNP_20715764	3.98	30.61	−1.78	Parent 2	Anuradha et al. (2012)
6	ZBR	*qZBR.2.1*	2	861	SNP_21560813–SNP_21617658	2.69	19.84	−1.46	Parent 2	
7	IPR	*qIPR.3.1*	3	878	SNP_21240772–SNP_21185917	8.15	34.76	−0.68	Parent 2	Swamy et al. (2018b)
8	IPR	*qIPR.6.1*	6	567	SNP_29657204–SNP_7127152	4.31	15.3	−0.5	Parent 2	Swamy et al. (2018b); Zhang et al. (2018); Dixit et al. (2019)
9	IPR	*qIPR.7.1*	7	700	SNP_16328271–SNP_15892815	3.74	12.67	−0.41	Parent 2	
10	AOC_IPR	*qAOC_IPR.7.2*	7	79	SNP_28458370–SNP_28114223	3.56	15.62	11.05	Parent 1	
11	AOC_IPR	*qAOC_IPR.11.1*	11	541	SNP_4832736-SNP_22702777	5.76	31.65	−17.17	Parent 2	Swamy et al. (2018b); Descalsota-Empleo et al. (2019a)
12	IBR	*qIBR.5.1*	5	158	SNP_24090722–SNP_24120920	4.16	33.03	−1.04	Parent 2	Kumar et al. (2019)
13	AOC_IBR	*qAOC_IBR.7.1*	7	669	SNP_15070854–SNP_16328271	2.7	22.13	−9.58	Parent 2	
14	SPY	*qSPY.7.1*	7	546	SNP_26142260–SNP_26042960	2.82	25.74	1.73	Parent 1	
15	AOC_SPY	*qAOC_SPY.7.1*	7	546	SNP_26142260–SNP_26042960	2.75	25.41	5.71	Parent 1	
16	TW	*qTW.1.1*	1	41	SNP_2154375–SNP_41166535	11.13	19.14	−1.42	Parent 2	Hua et al. (2002); Yadav et al. (2019)
17	AOC_TW	*qAOC_TW.1.1*	1	41	SNP_2154375–SNP_41166535	11.12	19.16	−5.75	Parent 2	Hua et al. (2002); Yadav et al. (2019)
18	TW	*qTW.3.1*	3	22	SNP_28761809–SNP_28820037	4.16	4.99	0.7	Parent 1	
19	AOC_TW	*qAOC_TW.3.1*	3	22	SNP_28761809–SNP_28820037	4.16	5.01	2.82	Parent 1	
20	TW	*qTW.6.1*	6	346	SNP_3438050–SNP_1619620	8.24	12.17	1.07	Parent 1	
21	AOC_TW	*qAOC_TW.6.1*	6	346	SNP_3438050–SNP_1619620	8.22	12.14	4.33	Parent 1	
22	TW	*qTW.7.1*	7	293	SNP_22039667–SNP_23128031	15.9	36.93	1.88	Parent 1	
23	AOC_TW	*qAOC_TW.7.1*	7	293	SNP_22039667–SNP_23128031	15.88	36.92	7.61	Parent 1	
24	TW	*qTW.7.2*	7	645	SNP_430820–SNP_1144612	4.54	5.68	0.74	Parent 1	
25	AOC_TW	*qAOC_TW.7.2*	7	645	SNP_430820–SNP_1144612	4.53	5.68	2.99	Parent 1	
26	TW	*qTW.12.1*	12	373	SNP_9255551–SNP_26268577	6.41	9.03	−0.97	Parent 2	
27	AOC_TW	*qAOC_TW.12.1*	12	373	SNP_9255551–SNP_26268577	6.4	9.02	−3.94	Parent 2	
28	PH	*qPH.1.1*	1	794	SNP_35161125–SNP_36994155	2.55	23.06	−7.2	Parent 2	Yan et al. (1999); Hemamalini et al. (2000); Yadav et al. (2019); Dixit et al. (2019)
29	AOC_PH	*qAOC_PH.1.1*	1	794	SNP_35161125–SNP_36994155	2.56	23.16	−8.73	Parent 2	Yan et al. (1999); Hemamalini et al. (2000); Yadav et al. (2019); Dixit et al. (2019)
30	AOC_NT	*qAOC_NT.12.1*	12	108	SNP_16467324–SNP_18705365	2.64	25.16	−3.68	Parent 2	

*Chr, chromosome; QTL, quantitative trait loci; cM, centimorgan; PV, phenotypic variance explained; LOD, logarithm of the odds; Add, additive effect; AOC, advanced over check; ZPR, zinc content in polished rice (ppm); ZBR, zinc content in brown rice (ppm); IPR, iron content in polished rice (ppm); IBR, iron content in brown rice (ppm); SPY, single plant yield (g); TW, test weight (g); PH, plant height (cm); NT, number of tillers per plant; Parent 1, recipient parent (PR116); Parent 2, donor parent (Ranbir Basmati).*

### Epistatic Interaction Analysis

#### 44 RILs With SNP and BLUE

A total of 28 epistatic interactions (PV 3.73 to 18.16%) were identified for five traits (ZBR, IPR, SPY, TW, and DFF) in the subset of 44 RILs with SNP_BLUE. Interestingly, out of five epistatic interactions for ZBR (>10 PV%), the locus on chromosome 1 has interacted with the locus on chromosome 2, which in turn has also shown interactions with four loci on chromosomes 5, 6, 7, and 10 (Figure 4). Interactions were also observed for AOC_ZBR on chromosome 3 with two loci of chromosomes 9 and 10, and another AOC_QTL for ZBR on chromosome 5 interacted with chromosome 10. A di-genic epistatic interaction within two loci of chromosome 5 was observed for AOC_QTL for IPR (PV 11.43%). Four epistatic interactions for SPY were found between two loci of chromosome 5; chromosome 1 with chromosome 3; and two loci of chromosome 2 and chromosome 2 with chromosome 10. For TW, a di-genic epistatic interaction with PV 15.57% was observed between chromosomes 1 and 9. Three interactions were noted for DFF between chromosomes 2 and 11 (PV 17.61%), chromosomes 4 and 7 (PV 18.16%), and within chromosome 12 (PV 17.1%) (Supplementary Table 21).

#### QTLs for Quality and Phytate (Single Environment)

With SSRs, major QTLs were identified for water uptake, kernel length after cooking, and elongation ration as *qWU.9.1* (PV 55.7%), *qKLAC.1.1* (PV 41%), and *qER.9.1* (PV 49.3%) from the donor parent and a moderate QTL as *qWU.8.1* (PV 21.74%) from the recipient parent. A major QTL for inorganic phosphate was also identified IP (*qIP.9.1*: PV 44.1%) from the recipient parent. Several minor QTLs < 10% for KL, ASV, and AC were also observed. Using SNPs, several major and moderate QTLs for hulling, milling, kernel breadth, kernel length/breadth ratio, and alkali spreading value were found as *qHULL.4.1* (PV 46.7%), *qMILL.1.1* (PV 58.2%), KB and KL/KB ratio (*qKB.3.1*: PV 24.3% and *qKB.10.1*: PV 22.1%), and *qASV.6.1* (PV 28.65%) from the recipient parent along with minor QTLs for water uptake. Interesting major QTLs for phytic acid (*qPA.2.1*: PV 58.32%) and total phytate (*qTP.2.1*: PV 50.6%) were identified from the donor parent (Supplementary Tables 22, 23).

#### Common QTL

One common grain Zn QTL was identified between SSR and SNP QTL in the present study. The SSR QTL for grain Zn, *qZPR.2.1* (PV 11.3%) was located in the 20.7–25.9-Mb region. The SNP QTL for grain Zn, *qZBR.2.1* (PV 19.84%), was located within the QTL region (21.5–21.6 Mb) identified by SSR on chromosome 2.

#### Co-localization of QTLs

Among the main SNP QTLs (PV > 10%), the region on chromosome 1 (SNP_21667551–SNP_20715764) was identified with ZPR and ZBR. The genomic region on chromosome 7 (SNP_22039667–SNP_26142260) has shown co-localization of QTL for IPR, TW, and SPY (Table 5). Co-localization of SSR-QTL was not observed in the present study.

#### Candidate Gene Analysis in the Identified QTLs of the Present Study

**FIGURE 5:**
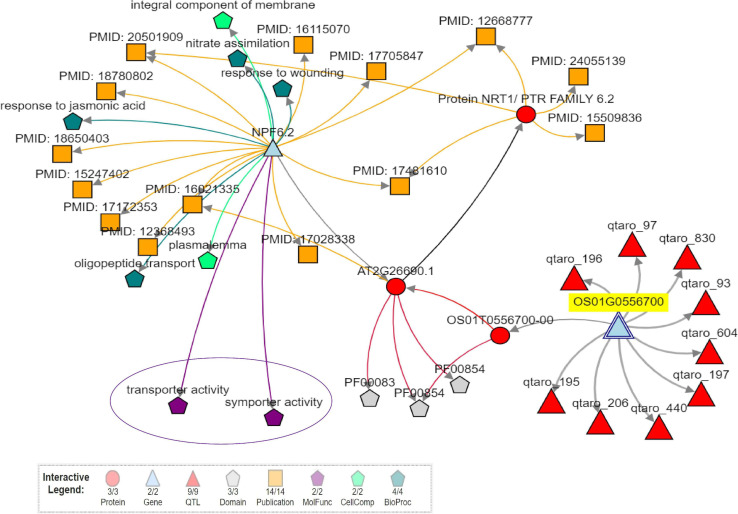
Network analysis of candidate gene (*Os01g0556700*-peptide transporter PTR2, putative, expressed) in a majorQTL*qZPR.1.1* using Knetminer.

Considering only the QTL for grain Zn and Fe content, we found 0 to 901 candidate genes for SSR-QTL and 7 to 337 candidate genes for SNP-QTL. Several transporter genes were observed in the identified QTL regions (Supplementary Tables 24, 25). WEGO analysis showed that the cation transport integral to the membrane under the biological process to be predominant with the candidate genes was identified in the QTL regions (Supplementary Figure 15). The identified putative candidate genes associated with mineral metabolism were selected from the annotated candidate genes in the two QTL regions. The role of two candidate genes in the Zn metabolism was evaluated using Knetminer (see text footnote 6) and also for the identification of relevant molecular functional pathways and temporal and spatial expression using RiceXPro version 3.0 (see text footnote 7) (Supplementary Figure 16). A network analysis of two genes using KnetMiner software in major QTL *qZPR.1.1*, viz., *Os01g0556700*, encoding peptide transporter PTR2, putative, expressed to be positioned within 21.03–21.04 Mb; and *Os01g0560200* encoding vesicle transport v-SNARE protein, putative, expressed to be positioned within 21.275–21.278 Mb^8^ from the QTL region of *qZPR.1.1* and *qZBR.1.1* (20.71–21.66 Mb) on chromosome 1 showed the linkage of the *Os01g0556700* gene linked with two genes, nine QTLs, and two molecular functions, viz., transport and symport activities (Figure 5). The second gene *Os01g0560200* was found to be linked with 25 genes, nine QTLs, nine phenotype traits, and 20 molecular functions (Supplementary Figure 17). These candidate genes which were found to be tightly linked with identified QTL are being further investigated.

#### Comparison of the Identified QTLs With Reported QTLs

All the QTLs identified for grain Zn and Fe content using SSRs coincided with the reported QTLs for earlier grain Zn and Fe content of rice (Supplementary Table 26). Only two novel QTLs (*qZPR.11.1* and *qIPR.7.1*) were identified in the present study with the remaining QTLs concurred with the reported QTLs for grain Zn and Fe (Supplementary Table 27). Out of several single season QTLs identified for quality, SSR QTLs for ASV and AC (*qASV.1.1* and *qASV.6.1*; *qAC.8.1* and *qAC.9.1*) and SNP QTLs for AC (*qAC.6.1* and *qAC.6.2*) coincided with the reported QTLs. For SPY, only QTLs observed on chromosome 12 shared the location with reported QTLs, while the remaining QTLs appear to be novel. Interestingly, many QTLs identified for grain mineral content coincided with reported QTLs for agro-morphological traits, yield, and yield-related components. Another important observation was the concurrence of identified QTLs for inorganic phosphorus (IP), total phosphate (TP), and phytic acid (PA) with the reported QTLs for yield and yield-related components and total number of tillers (Supplementary Tables 28, 29).

## Discussion

As per the recent global nutrient database, availability of micronutrients per day per person in South Asia is relatively poor compared to the world’s availability^9^. More than 32% of women across the world and 36.6% in Asia are estimated to be anemic (FAO et al., 2019)^10^. One third of the world’s population was reported to lack sufficient Zn nutrition (White and Broadley, 2011). The climate change through elevated CO_2_ has been also reported to be leading toward deficiencies of Zn, protein, and Fe for women of reproductive age and children (Smith and Myers, 2019). Out of suitable agricultural interventions to address micronutrient malnutrition, enriching grain micronutrient density through biofortification and encouraging dietary diversity were suggested to be ideal and long-term sustainable strategies (Bouis et al., 2019). For a staple food crop like rice, any incremental increase of micronutrients would have an impact on the malnutrition affecting most of the developing countries with rice as the principal calorie food. Enhancing micronutrient density in the staple crops, especially in cereals, has been demonstrated through release of several biofortified varieties across the world. In rice, a few biofortified varieties with high grain Zn have been developed and released in Asian countries (HarvestPlus and FAO, 2019). The released Zn-biofortified rice varieties were developed using the conventional breeding approach based on phenotyping for grain Zn and yield (Nakandalage et al., 2016; Khan et al., 2019). The use of MAS for major QTL-associated high grain Zn would be a focused approach for accelerating the development of Zn-biofortified rice varieties (Mahender et al., 2016). Based on the requirements and bioavailability of Zn, the recommended target content in polished biofortified rice grains has been enhanced to 28 ppm (Bouis and Saltzman, 2017). To meet the enhanced levels of high grain Zn in rice, identification and deployment of major QTLs would be useful to increase the efficiency of the breeding program and expedite the development of biofortified rice varieties with high grain Zn. In our study using the RIL population, we could identify two major QTLs for grain Zn using markers (SSRs and SNPs) based on BLUE values, a common QTL for grain Zn content on chromosome 2 across SSR and SNP maps, and also six promising lines with high grain Zn (mean > 28.0 ppm in polished rice) and yield (>20 g SPY).

A wide genetic variability within the RIL population for the 10 agro-morphological traits along with grain Zn and Fe was observed across two/four environments, which is in congruence of the published studies for grain Zn, Fe, yield, and other agronomic traits in mapping populations (Dixit et al., 2019; Calayugan et al., 2020). Continuous distribution of the studied traits including grain Zn suggests involvement of several genes leading to complex genetic action. Nine traits of the study showed high heritability values suggesting the early generation selection strategy for their improvement which also supported the observations of earlier reports (Calayugan et al., 2020). High heritability and variability for grain Zn values within the RIL population of the present study indicate its suitability for mapping. The grain Zn content in polished rice was found to be relatively higher in the dry season with the maximum values up to a recommended level of 28 ppm in the present study. Seasonal variations for grain Zn content during wet and dry seasons were observed as reported earlier (Swamy et al., 2018a; Descalsota-Empleo et al., 2019a; Dixit et al., 2019). Six transgressive variants obtained for grain Zn have also shown promising yield with SPY of >20 g. Transgressive variation for grain Zn content in RIL populations is possibly due to the pyramiding of the effects of moderate and minor QTL from both parents as happens in most of the quantitative traits (Lu et al., 2008; Gande et al., 2013; Zhang et al., 2014; Yu et al., 2015).

Rice consists of hull and inner edible portion including 89–94% starchy endosperm, 1–2% pericarp, 4–6% seed coat and aleurone, and 2–3% embryo (Juliano, 1972). Differential accumulation of Zn and Fe in the different parts of rice grain has been studied in detail (Liang et al., 2008; Lombi et al., 2009; Hansen et al., 2012). A significant amount of nutritionally important mineral elements accumulates in rice bran (embryo and aleurone layers), whereas a lower amount (6–9%) is found in the endosperm (Lamberts et al., 2007). Zinc is distributed throughout the endosperm (Takahashi et al., 2009; Johnson, 2013), which because of its relatively large mass accounts for 75% of grain Zn (Wang et al., 2011). Zn is distributed from the aleurone layer to the inner endosperm with more than one half of the total Zn present in the endosperm; Fe is localized in the aleurone layer (Iwai et al., 2012). The Fe concentration in the bran is seven times higher than that of the hull and endosperm, but Zn in the bran is only three times higher (Lu et al., 2013). Thus, the low values observed in polished rice substantiated the distribution of Fe in rice grain. Though a wide variation was observed for grain Fe content in brown rice with a maximum of 18 ppm, the range was limited up to 9.5 ppm in polished rice (Majumder et al., 2019; Calayugan et al., 2020). Highly significant positive correlations were obtained among ZPR, ZBR, IPR, and IBR, which is expected owing to the common metabolic pathways for uptake, assimilation, and translocation to the grains (Stangoulis et al., 2007; Kumar et al., 2014; Xu et al., 2015; Bashir et al., 2016; Swamy et al., 2018a; Descalsota-Empleo et al., 2019a; Wattoo et al., 2019). Among the agro-morphological traits, only days to 50% flowering has shown a moderate negative association with grain Zn. Varied or contradicting correlations of grain Zn with other agro-morphological traits across the studies involving different mapping populations and environments underscore the difficulty of breeding for high grain Zn (Calayugan et al., 2020; Jeong et al., 2019). Among the correlations obtained between the quality and grain Fe and Zn, the associations of IPR and IBR with kernel length after cooking (KLAC) and elongation ratio (ER) need further studies. The correlations of inorganic phosphate (IP) with kernel breadth (KB) and head rice recovery (HRR) also need to be validated.

The PCA clearly showed the role of four PCs (Eigen values are ≥1) contributing around 68% of variability. Nutritional traits comprised the first and fourth PCs with yield-attributing traits forming the second and third PCs underscoring the suitability of the material generated in the study for the improvement of grain nutrient content and yield. For a subset of 44 RILs, the first PC was also mainly attributed by nutrient traits; the second and third PCs were influenced by yield-attributing traits, and the fourth PC was grouped by both yield and nutrient traits.

Stepwise regression analysis of 190 RILs revealed the interdependence of ZPR, ZBR, IPR, and IBR for higher grain Zn/Fe content in brown and polished rice and also PH and GW for SPY. Differential control for grain Fe content with TW was obtained as a negative factor in polished rice and as a positive factor in brown rice, which could be because of the volume-to-surface ratio of the rice grain and the area covered by the bran layer. The interesting observation from the stepwise regression analysis in subset of 44 RILs with contrasting grain Zn was the negative association of ZPR with SPY and ZBR with SPY, PL, and TW. Grain Fe content of contrasting 44 RILs has shown DFF and TW as negative factors and PL and ZPR as positive factors in polished rice, and only ZPR as positive factor in brown rice. It is also interesting to note that correlation analyses indicated that DFF was negatively correlated with grain Zn. Stepwise regression analyses also showed DFF as a negative factor for Fe content in polished rice. The negative association of SPY and ZBR and ZPR in the subset of 44 RILs through stepwise regression analyses reiterated the negative association of grain mineral content and yield. In general, there is a negative association between grain Zn and yield; however, it is possible to obtain desirable recombinants for grain Zn and yield as observed in the present study.

Six promising RILs (G32, G17, G8, G18, G15, and G7) were identified for ZPR with >28 ppm based on the stability and G × E interaction analyses for the subset of 44 RILs across four environments (E1–E4). For SPY, promising RILs environment-wise (G1 and G17: wet season and G6: dry season) as well as across environments were noted (G3 and G19). G17 found to be promising RIL for ZPR and SPY also (mean of 28.3 ppm for ZPR and 23.5 g for SPY). Though there is negative association between high yield and grain Zn, the promising lines were identified as in the present study with high grain Zn and yield, though less in number, confirming the possibility of obtaining their combination (Swamy et al., 2016; Pradhan et al., 2020; Rao et al., 2020). G32 and G17 were also found to be promising for ZBR as expected. Though for IBR, G32, G3, and G18 were noted to be promising across environments with more than 16 ppm, but for IPR, the donor parent was found to be promising. Similarly, the best and stable performers were identified for TW, PH, PL, NT, and DFF. Through AMMI and GGE biplot models, the stable performers across the environments were identified and the total phenotypic variance was partitioned into individual factors (Gauch, 2006). In rice, stable performers for yield were identified across environments using AMMI and GGE (Balakrishnan et al., 2016, 2020). Through the Which Won Where/What plot, common winners could not be found across four environments for the 10 traits of study which could be due to the variability of performance of the RILs across wet and dry seasons. For rice grain Zn and Fe, stability and G × E analyses are generally used for identification of stable donors (Suwarto and Nasrullah, 2011; Ajmera et al., 2017; Babu et al., 2020; Naik et al., 2020). Considering the wide variability observed for the breeding lines with high grain Zn and Fe, stability and G × E analyses are being recently applied for selecting promising RILs in cereals. The contribution of environmental variation for grain Fe and Zn along with other agronomic traits in a RIL population of sorghum was demonstrated through a genotype × environment interaction, correlation, and GGE biplot analyses (Phuke et al., 2017). Stable RILs with higher grain Fe and Zn content were also identified in RILs of pearl millet using AMMI and GGE biplot analyses (Singhal et al., 2018). Different stable breeding lines were identified for different environments among eight Zn biofortified lines through stability and G × E analyses (Inabangan-Asilo et al., 2019).

Out of eight QTLs identified with 102 SSRs for five traits (ZPR, IBR, SPY, PH, and DFF), only two QTLs were identified with PV > 10%, viz., *qZPR.2.1* (spanning 6.3 Mb region) and *qIBR.5.1* (spanning 1.9 Mb). Several major and moderate QTLs for grain Zn and Fe were identified using SSRs in rice (Anuradha et al., 2012; Hu et al., 2016; Swamy et al., 2018b; Dixit et al., 2019). Most of the reported QTLs based on SSRs need to be validated in alternative mapping populations for their deployment in rice biofortification.

Based on GBS analyses, 1035 polymorphic SNPs between parents and subset of 44 RILs were used to construct a linkage map in the present study. Though the subset of 44 RILs is a small number, the subset has shown normal distribution for the Zn content. The range of Zn content of the subset of 44 RILs was 11.5 to 31 ppm, and the range of 190 RILs was 11 to 31 ppm. Also, it was also observed that the percentage of 190 RILs with Zn content was >20 ppm was 49.5% and <20 ppm was 51.5% (1:1 ratio). A similar distribution of Zn content was also observed in the subset of 44 RILs as >20 (50%) and <20 ppm (50%) (1:1 ratio). Thus, the assumption was that the subset of 44 RILs was the representation of 190 RILs for GBS analyses. In the present study, the less number of RILs subjected to GBS and subsequent QTL identification was compensated by the phenotype data of 44 RILs from four environments and extensive coverage of 12 chromosomes with 1305 SNP data points. Earlier linkage maps for identifying QTL associated with grain Zn reported SNPs ranging only from 296 to 541 (Swamy et al., 2018a; Descalsota-Empleo et al., 2019a; Calayugan et al., 2020). Out of 16 major QTLs (PV > 10%) from 19 QTLs identified with SNPs, only 11 QTLs were further analyzed for candidate genes as the interval was too large for two QTLs. The physical position of the identified QTLs in the rice genome spanned only a region of 0.1 to 3 Mb, which makes the QTL amenable to marker-assisted introgression to the genotypes with desirable background. The major QTLs on *qZPR.1.1* (PV 37.84%) and *qZPR.11.1* (PV 15.47%) identified in the present study can be deployed in the breeding program for high grain Zn as it is consistent across the environments (seasons). QTLs for grain Zn/Fe were mostly reported in brown rice (Stangoulis et al., 2007; Garcia-Oliveira et al., 2009; Du et al., 2013; Kumar et al., 2014; Zhang et al., 2014) and a few in polished rice (Lu et al., 2008; Yu et al., 2015). Only one study identified QTLs for brown and polished rice in the Backcross Inbred Line mapping population of *Oryza sativa* × *O. rufipogon* (Yu et al., 2018), however with no common QTLs between brown and polished rice have been found. Since in our study we mapped QTLs for brown and polished rice, the identified consistent major QTL *qZPR.1.1* (PV 37.84%) overlapping with *qZBR.1.1* (PV 30.61%) QTL reinforced the location of QTL for grain Zn.

Though QTLs were identified for 190 RILs with 102 SSR markers and 44 RILs with 1035 SNPs in the present study, only one common QTL on chromosome 2 for grain Zn was found across the SNP map (*qZBR.2.1*: PV 19.84%) (21.5–21.6-Mb region) and the SSR map (*qZPR.2.1*: PV 11.3%) (20.7–25.9-Mb region). Between the two environments for SSRs (E1, E2 for SSR) and among the four environments for SNP (E1, E2, E3, and E4 for SNP), we found some common QTLs. However, when the BLUEs were considered for the identification of QTLs, the number of common QTLs was very less. The probable reasons for not finding common QTLs between SSR and SNP could be attributed to the number of environments included in the QTLs of SNP analyses. Adding of two more environments E3 and E4 has reduced the common QTLs. Since BLUEs were advised for the identification of major and stable QTLs for grain Zn content (Calayugan et al., 2020), two more environments (E3 and E4) were added for the identification of stable SNP QTLs.

Zn and Fe are needed as essential mineral elements to the plant for its growth and development (Palmer and Guerinot, 2009); thus, an optimum concentration of grain Zn and Fe is always present in rice. Hence, we have included additional parameter as advantage over check (AOC) to the 10 traits of study for identification of QTLs. The rationale behind AOC, especially for grain Zn and Fe traits, is an optimum level of Zn and Fe which are present in the endosperm by default controlled by a set of genes/QTLs. Any additional amount of Zn/Fe in polished could be due to either different alleles of the same set of genes or different genes. Thus, two new AOC QTLs were identified with SSRs and three novel QTLs were observed with SNPs, suggesting AOC as a promising approach for identifying QTLs for grain mineral content.

Quantitative trait loci covering most of the chromosomes were reported for grain Fe and Zn in various biparental mapping populations as in F_2_, RILs, doubled haploid (DH), back cross inbred lines, and introgression lines (Stangoulis et al., 2007; Garcia-Oliveira et al., 2009; Anuradha et al., 2012; Kumar et al., 2014; Zhang et al., 2014; Xu et al., 2017; Swamy et al., 2018b; Descalsota-Empleo et al., 2019a). Most of the reported QTLs could not be deployed in breeding for biofortified rice varieties as they are genotype and environment specific. Analyses for QTLs using BLUEs enhanced the rigor of the identified QTL for their utility in breeding program of biofortification.

Nineteen significant digenic epistasis interactions (ZPR, ZBR, AOC_IPR, IBR, SPY, TW, and DFF) were detected with PV > 10% with SSR and SNP, suggesting the complex genetic regulation for the traits of the study. However, none of the identified digenic interactions were found to be involved with main QTLs. Similar observations were earlier reported for epistatic interactions for grain Zn in rice (Lu et al., 2008; Norton et al., 2010; Zhang et al., 2014; Descalsota-Empleo et al., 2019b). Involvement of main effect QTLs in epistatic interactions suggests that the effect of single-locus QTL is mostly dependent on the alleles of other loci (Lu et al., 2008). The identification of main-effect QTL for grain Zn without association of epistatic interactions is counter-intuitive because the grain Zn content involves a complicated metabolic process of uptake, transport, assimilation, and remobilization controlled by temporal and spatial regulation of various genes (Bashir et al., 2016). Hence, the identified main-effect QTL is being further characterized for its genetic action.

Since the quality of rice grains is associated with nutritional quality as the ratio of bran to endosperm (surface to volume) which is greatly affected by grain shape (length, breadth, thickness), data on grain quality and cooking quality were included for the subset of 44 RILs in the present study. Grain traits like weight, length, thickness, and breadth found to be negatively correlated with grain Zn and Fe in rice (Jeong et al., 2019). Co-localization of QTLs of grain mineral elements with quality QTL was also reported (Zhang et al., 2014). Only grain Fe has shown correlation with kernel length after cooking, elongation ratio, and alkali spreading value based on single environment data in our study. The role of grain Fe in cooking quality needs confirmatory studies.

The total phosphate in the seed was studied as phytate phosphate and inorganic phosphate in the subset of 44 RILs of the present study. *Myo*-inositol 1,2,3,4,5,6-hexakisphosphate (InsP6), commonly known as phytic acid (PA), is the principle storage form of phosphorus (P) in cereal grains and may account for 65–85% of the total seed P (Raboy et al., 2000). In rice grains, approximately 70% of the total seed phosphorus is found in the form of phytic acid with ∼80% more present in the aleurone and pericarp and less than 10% in the embryo (O’Dell et al., 1972; Iwai et al., 2012). The remaining P is in the form of soluble inorganic phosphate (Pi: approximately 5%) and cellular P (approximately 10 to 20% of the total seed P), which is found in nucleic acids, proteins, lipids, and sugars (Larson et al., 2000*).* Expected correlations were observed between phytic acid and total phosphate among the subset of 44 RILs in the present study. PA is negatively charged and, thus, strongly chelates cations such as Fe and Zn and usually exists as mixed salts referred to as phytate or phytin in cereals (Raboy, 2009). Most notably, Zn and Fe deficiencies are reported to be linked to high PA intake (Al Hasan et al., 2016). Two QTLs for PA content were earlier identified to chromosomes 5 and 12 explaining 24% and 15% of the total phenotypic variation (Stangoulis et al., 2007*).* Unlike the study of Stangoulis et al. (2007), neither correlation with grain Zn/Fe nor co-localization with grain Fe/Zn QTLs was found in our study. The grain Zn and its association with phytate in the mapping populations need to be elucidated in future.

Several-candidate-gene-associated transporter activity was observed in the identified QTL; based on the network analyses in the present study, we narrowed down to two genes in the identified QTL and gene-associated nutrient homeostasis. The genes are being functionally characterized. Though information on grain Zn and Fe metabolism genes is available to some extent, genes associated with uptake, transport, assimilation, and remobilization of Zn and Fe still need to be characterized in rice. The concurrence of the identified QTLs with the reported QTLs reiterates rigor of the identified QTLs, at the same time novel QTLs explaining high phenotypic variance are useful for deployment in the breeding programs and identification of new genes associated with high grain Zn (Kawakami and Bhullar, 2018). The two RILs from the present study with the promising QTLs for grain Zn in polished rice and yield, viz., RP6211-PR/RIL-Q8 and RP6211-PR/RIL-Q181, have been selected and nominated to evaluation during wet season2020 under Biofortification trial of All India Coordinated Rice Improvement Programme, national varietal release program in India.

## Conclusion

In conclusion, the RIL population of the study showed wide variation for agro-morphological traits, yield, grain Fe, and Zn across environments. Through stepwise regression analyses, factors among the agro-morphological and yield traits affecting the grain Zn and Fe were identified. Through AMMI, performance of RILs was analyzed for their stability across environments. The promising RILs, thus identified with grain Zn in polished rice >28 ppm and 20 g single plant yield, were nominated in the national evaluation programs for biofortified rice varieties. Several QTLs have been identified for agro-morphological traits, yield, and grain Fe and Zn using SSRs and SNPs. Inclusion of both brown and polished rice along with advantage over check strengthened the analyses of QTL in the present study. QTLs were also identified for single-season data of grain quality along with total seed phosphorus in the subset of RILs. Two major QTLs for grain Zn in polished rice spanning only <3 Mb genomic fragment offers scope for their deployment in rice biofortification. The potential of the two candidate genes in the QTLs were confirmed by network analyses.

## Data Availability Statement

The data has been submitted to NCBI and the accession number has been provided in the manuscript as submitted to the sequence read archive at NCBI under BioProject No: PRJNA698265, https://www.ncbi.nlm.nih.gov/sra/.

## Author Contributions

CN conceptualized the idea. KS, PM, and UC developed RILs and conducted and supervised the field experiments. CN, KS, SR, SB, and KJ carried out the data analysis. KS and CN prepared the manuscript. CN, JK, KS, SP, SHR, LS, and SV edited the manuscript. All authors contributed to the article and approved the submitted version.

## Conflict of Interest

The authors declare that the research was conducted in the absence of any commercial or financial relationships that could be construed as a potential conflict of interest.

## Footnotes

1 www.ricepedia.org

2 http://www.gramene.org

3 http://www.isbreeding.net/software/default.aspx

4 http://rapdb.dna.affrc.go.jp/viewer/gbrowse/irgsp1

5 http://qtaro.abr.affrc.go.jp/

6 http://knetminer.rothamsted.ac.uk/Oryza_sativa/

7 https://ricexpro.dna.affrc.go.jp/

8 https://rapdb.dna.affrc.go.jp/

9 https://nutrition.healthdata.org/global-nutrient-database

10 https://globalnutritionreport.org/reports/global-nutrition-report-2018/
