# Supplementary material for: Identification of Promising RILs for High Grain Zinc Through Genotype × Environment Analysis and Stable Grain Zinc QTL Using SSRs and SNPs in Rice (Oryza sativa L.)
Source: Front Plant Sci. 2021 Feb 18;12:587482. doi: 10.3389/fpls.2021.587482 (PMC7930840; doi:10.3389/fpls.2021.587482)
Supplement: Supplementary file 1 [file Data_Sheet_1.docx]

Supplementary Figure 1: Frequency distribution of 190 RILs for ZPR- Zinc content in Polished Rice (ppm); ZBR- Zinc content in Brown Rice (ppm); IPR- Iron content in Polished Rice (ppm); IBR- Iron content in Brown Rice (ppm); SPY- Single Plant Yield(g); TW: Test weight (g); PH- Plant Height (cm); PL- Panicle Length (cm); NT-Number of Tillers per plant; DFF-Days to fifty percent flowering (days).


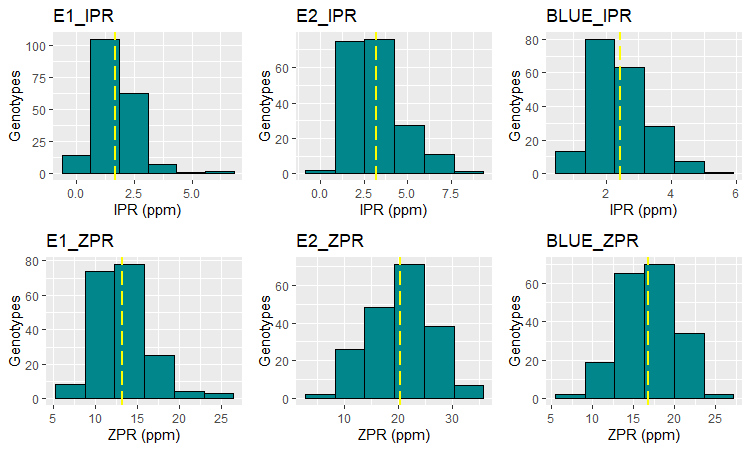

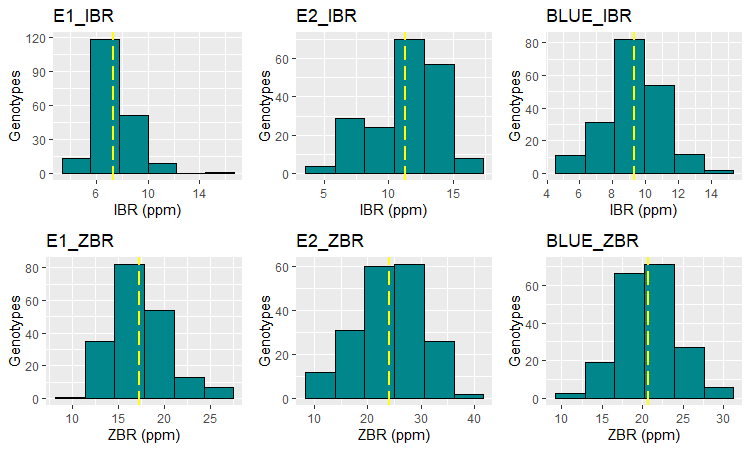

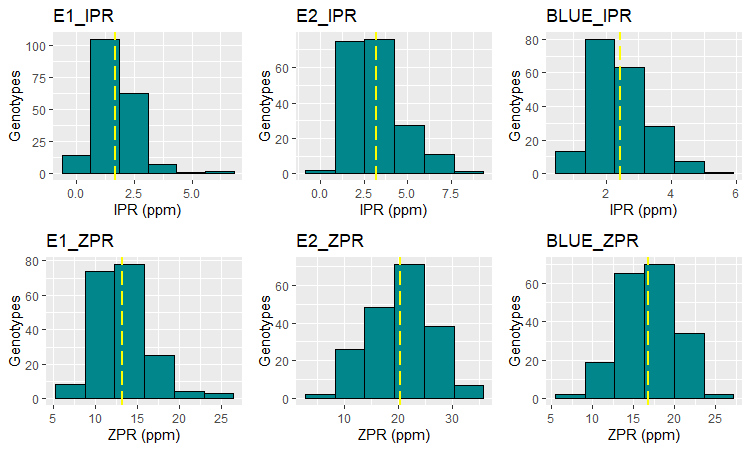

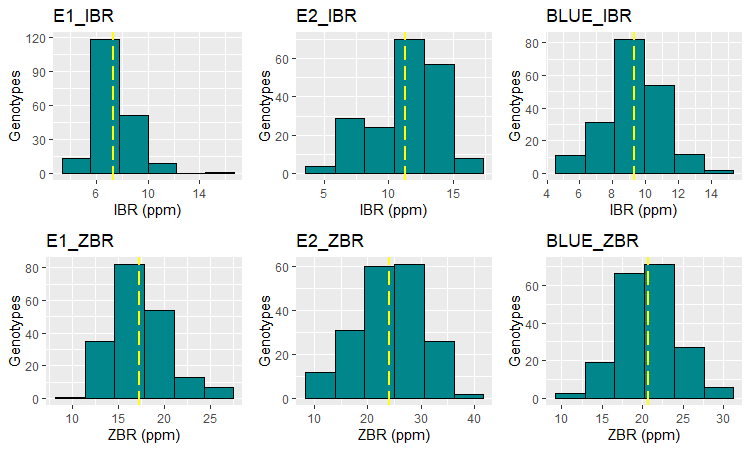

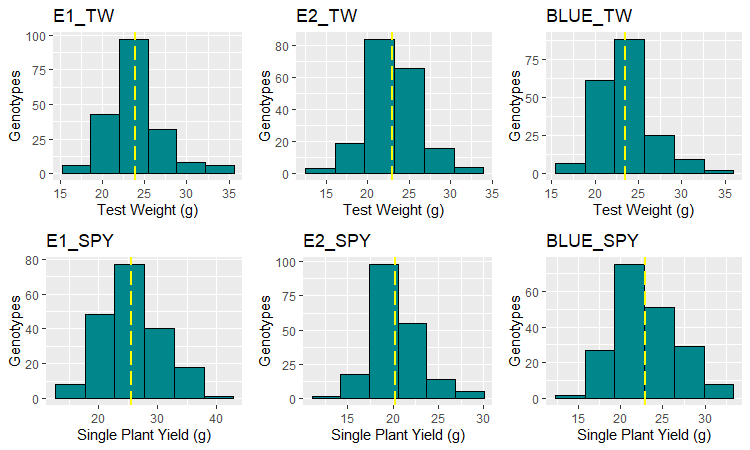

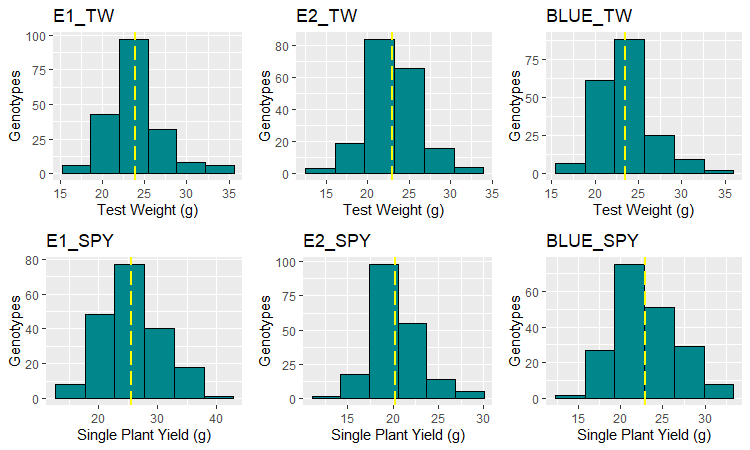

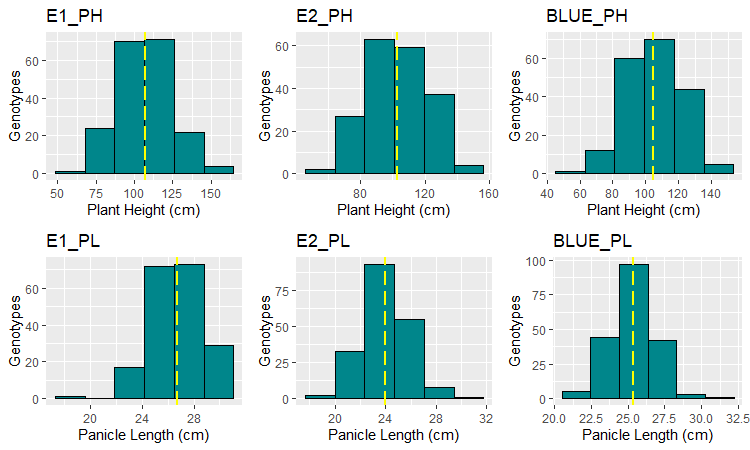

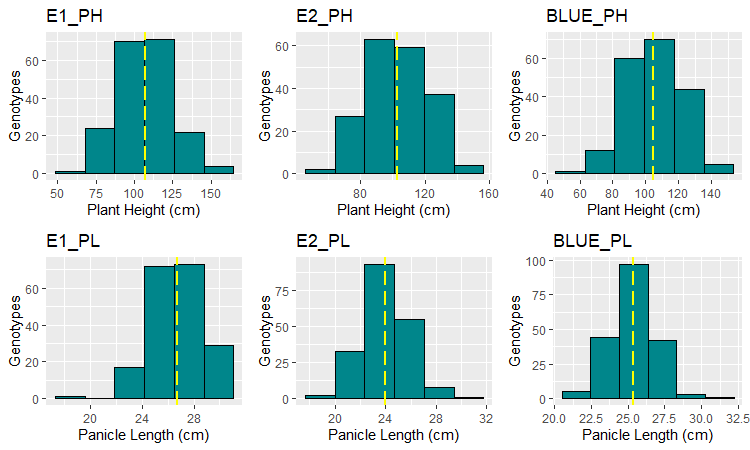

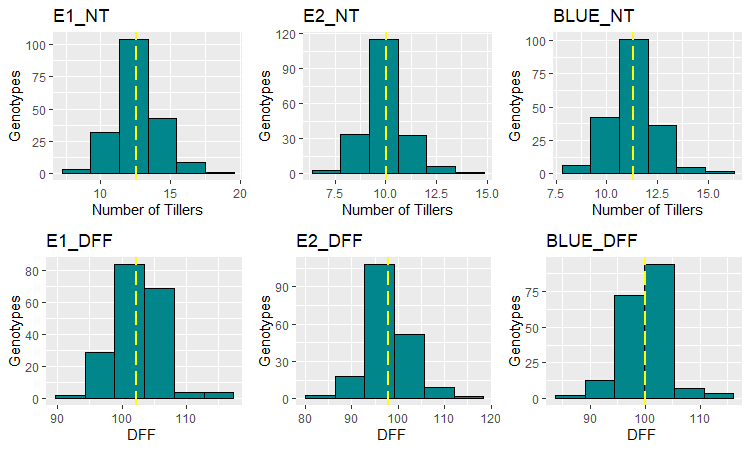

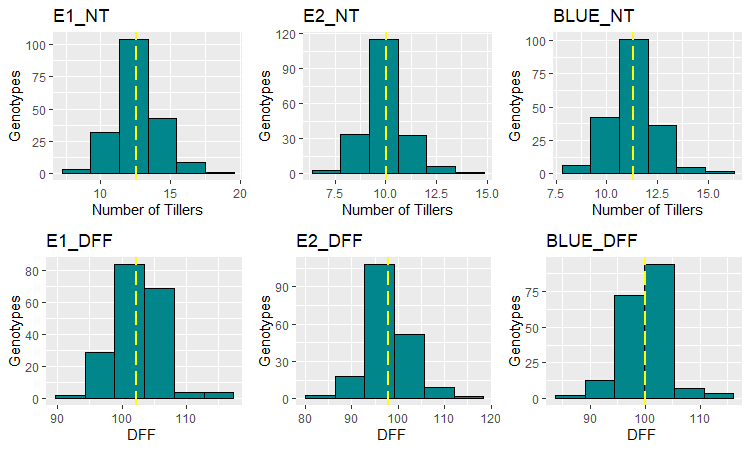


Supplementary Figure 2: Box plots for 190 RILs for ZPR- Zinc content in Polished Rice (ppm); ZBR- Zinc content in Brown Rice (ppm); IPR- Iron content in Polished Rice (ppm); IBR-Iron content in Brown Rice (ppm); SPY- Single Plant Yield(g); TW: Test weight (g); PH- Plant Height (cm); PL- Panicle Length (cm); NT-Number of Tillers per plant; DFF-Days to fifty percent flowering (days).


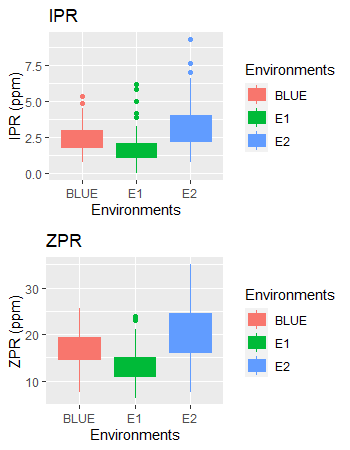

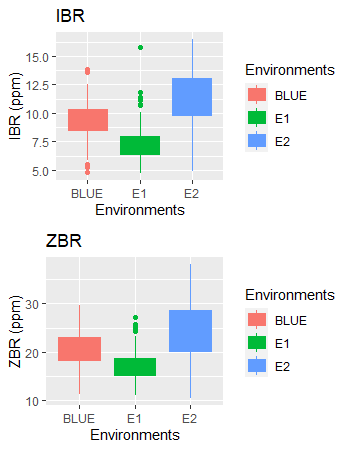

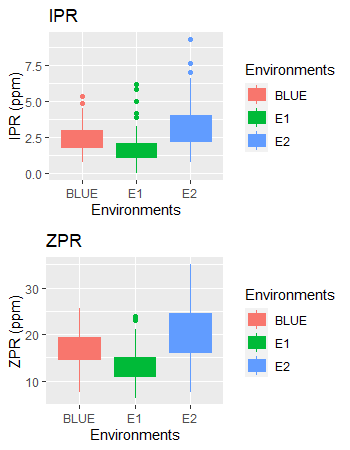

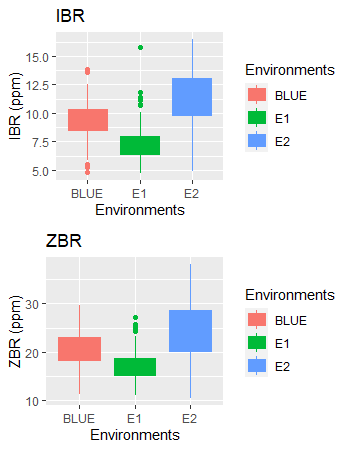

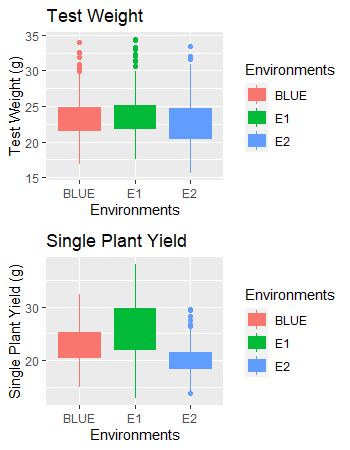

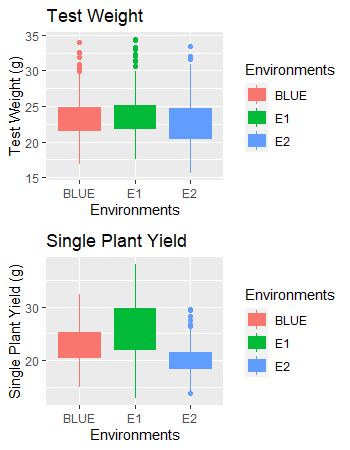

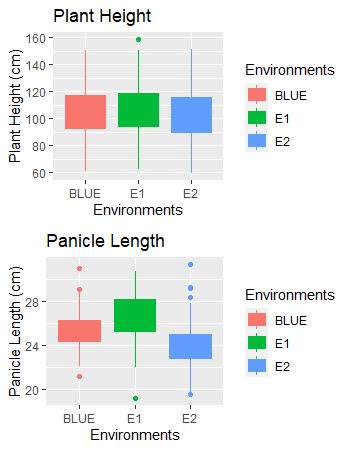

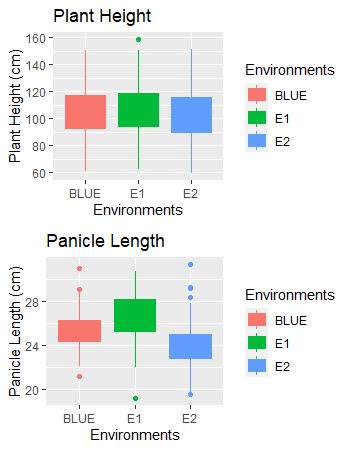

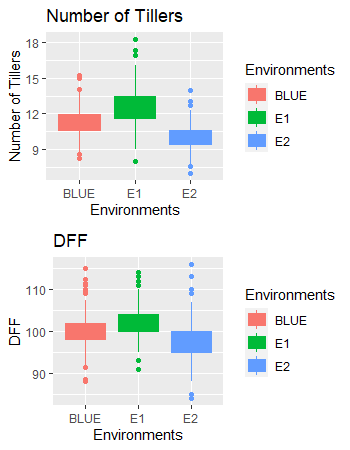

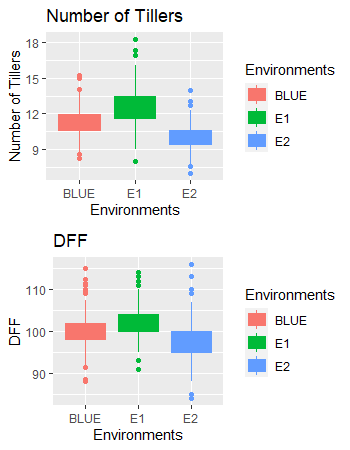


Supplementary Figure 3: Frequency distribution of 44 RILs for ZPR- Zinc content in Polished Rice (ppm); ZBR- Zinc content in Brown Rice (ppm); IPR- Iron content in Polished Rice (ppm); IBR- Iron content in Brown Rice (ppm); SPY- Single Plant Yield(g); TW: Test weight (g); PH- Plant Height (cm); PL- Panicle Length (cm); NT-Number of Tillers per plant; DFF-Days to fifty percent flowering (days).


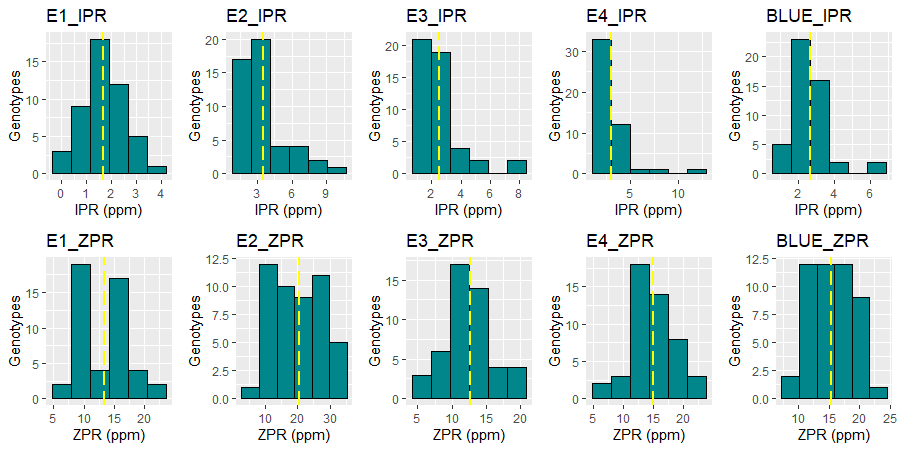

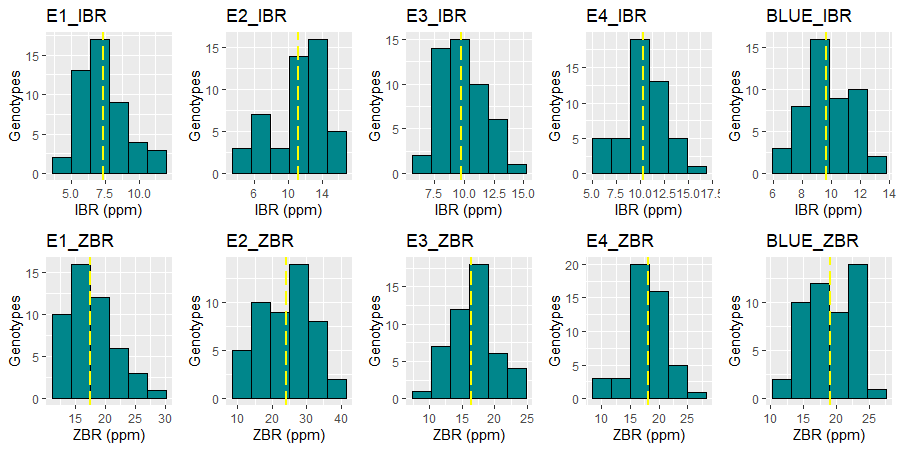

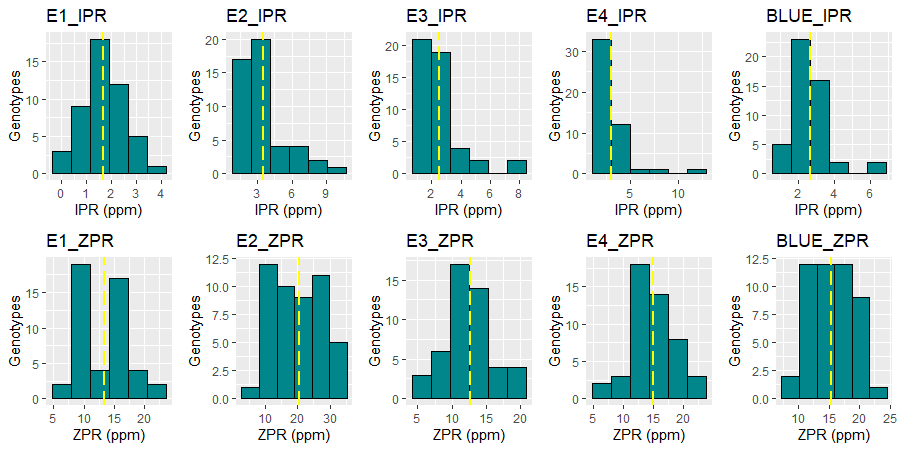

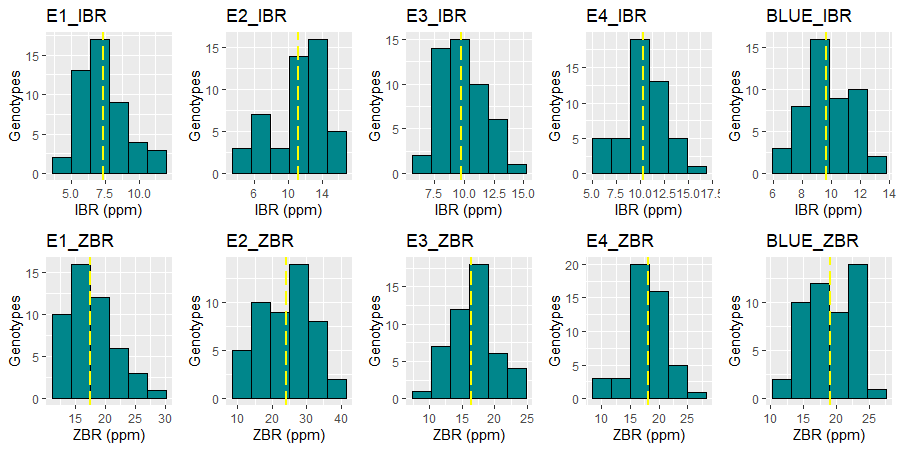

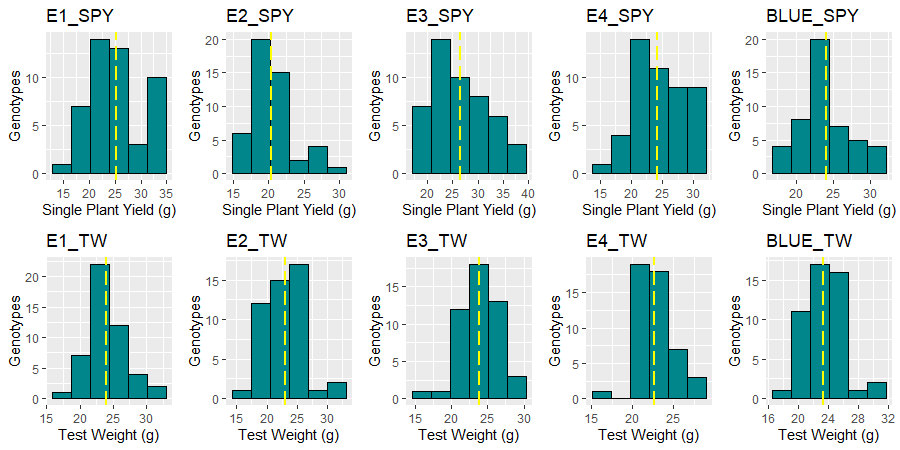

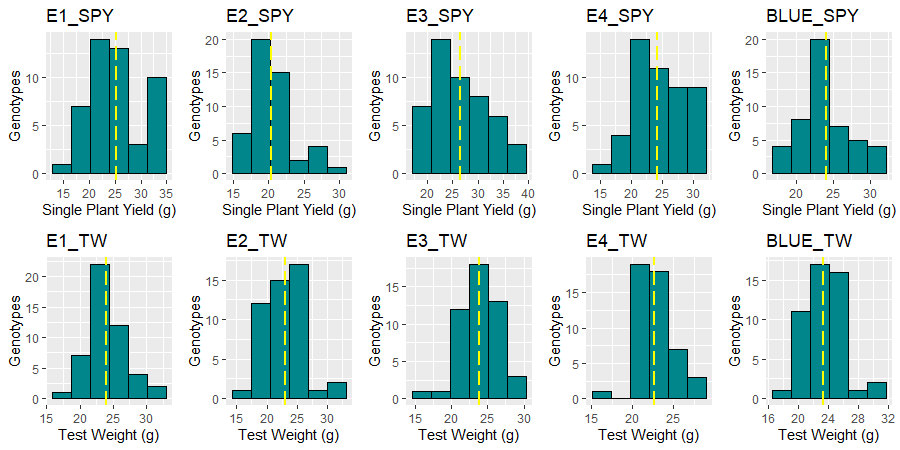

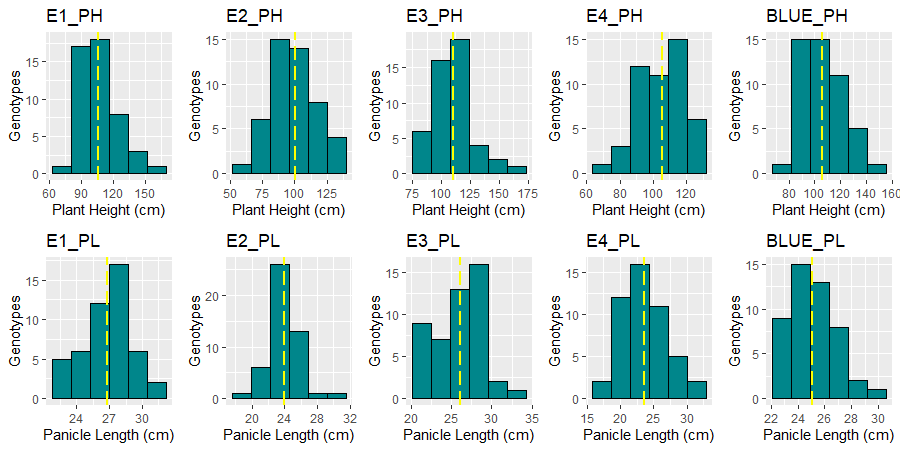

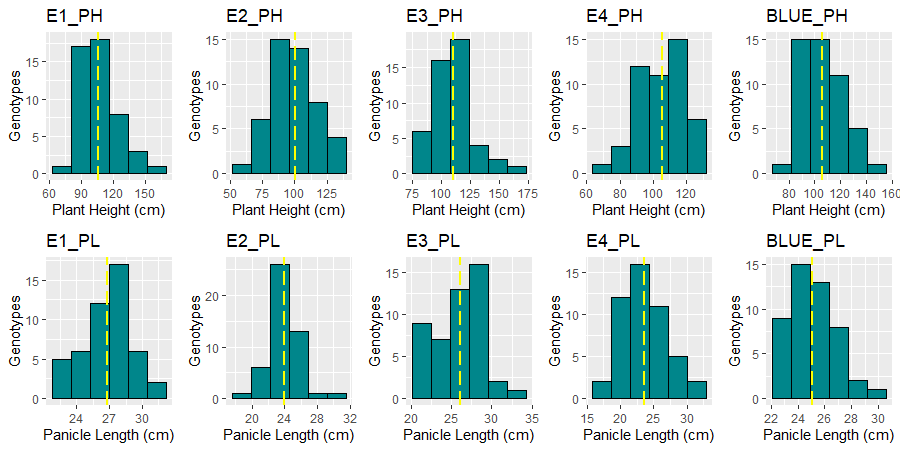

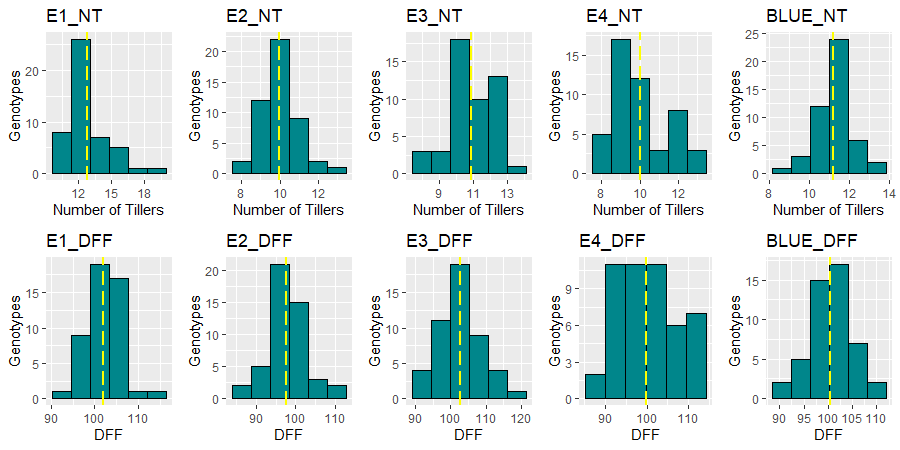

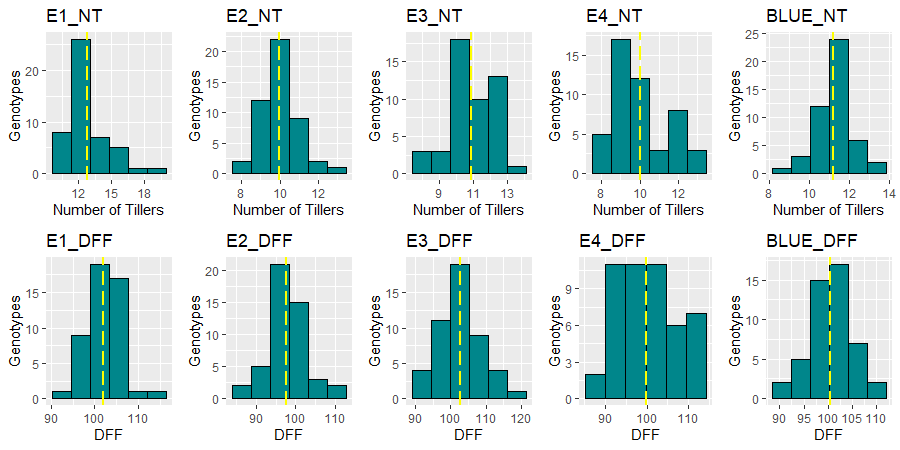


Supplementary Figure 4: Box plots for 44 RILs for ZPR- Zinc content in Polished Rice (ppm); ZBR- Zinc content in Brown Rice (ppm); IPR- Iron content in Polished Rice (ppm); IBR-Iron content in Brown Rice (ppm); SPY- Single Plant Yield(g); TW: Test weight (g); PH- Plant Height (cm); PL- Panicle Length (cm); NT-Number of Tillers per plant; DFF-Days to fifty percent flowering (days).


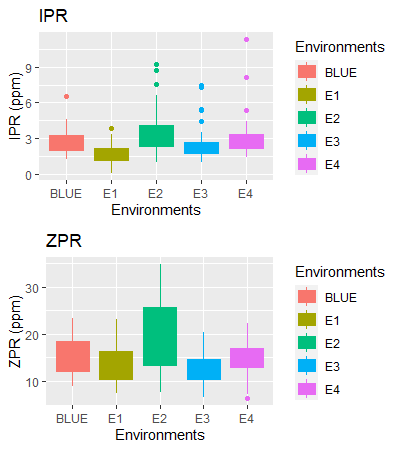

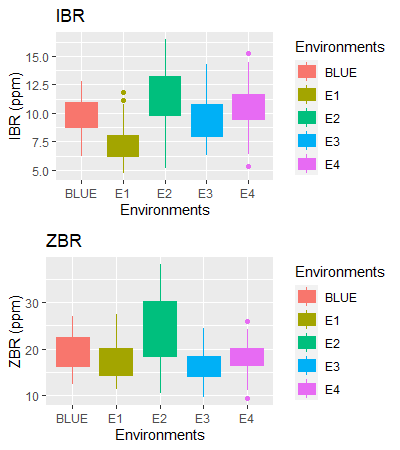

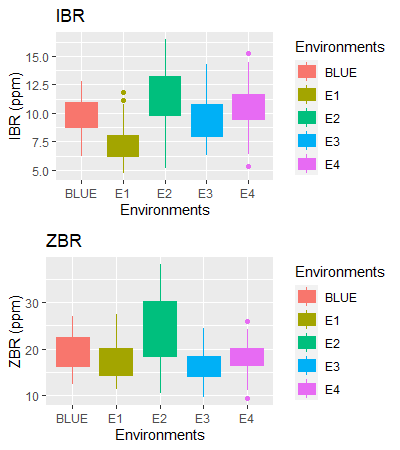

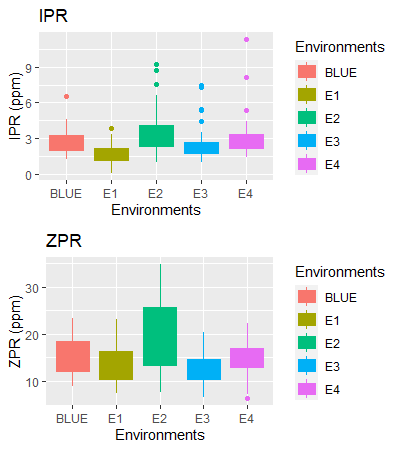

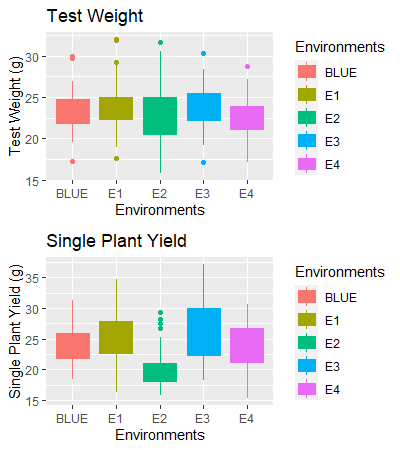

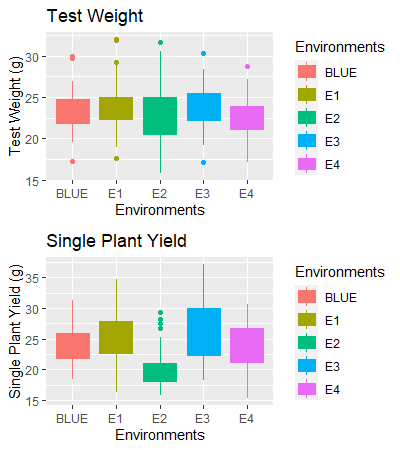

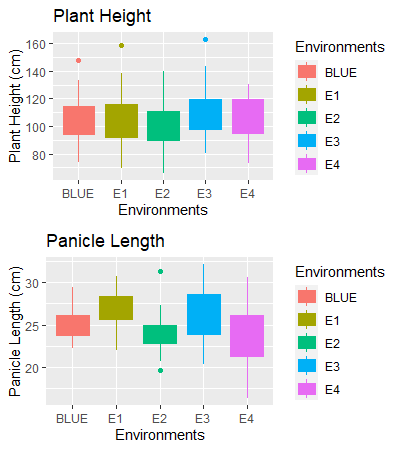

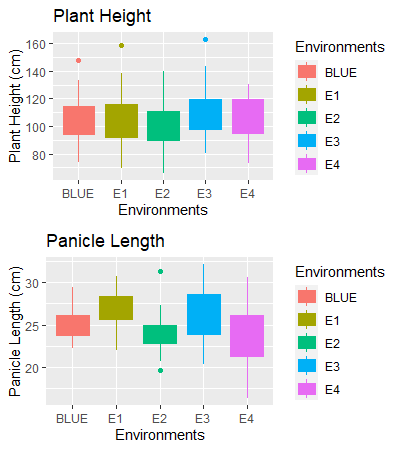

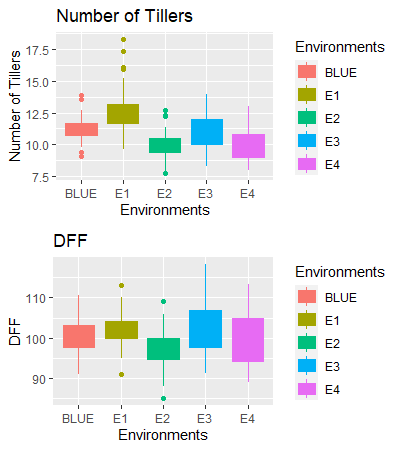

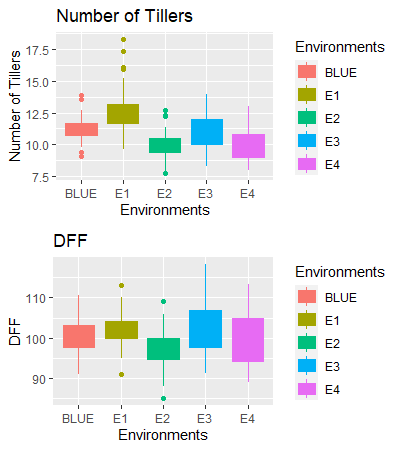


Supplementary Figure 5: D-Square analysis of 190 and 44 RILs for ten traits based on morphological characters.

^
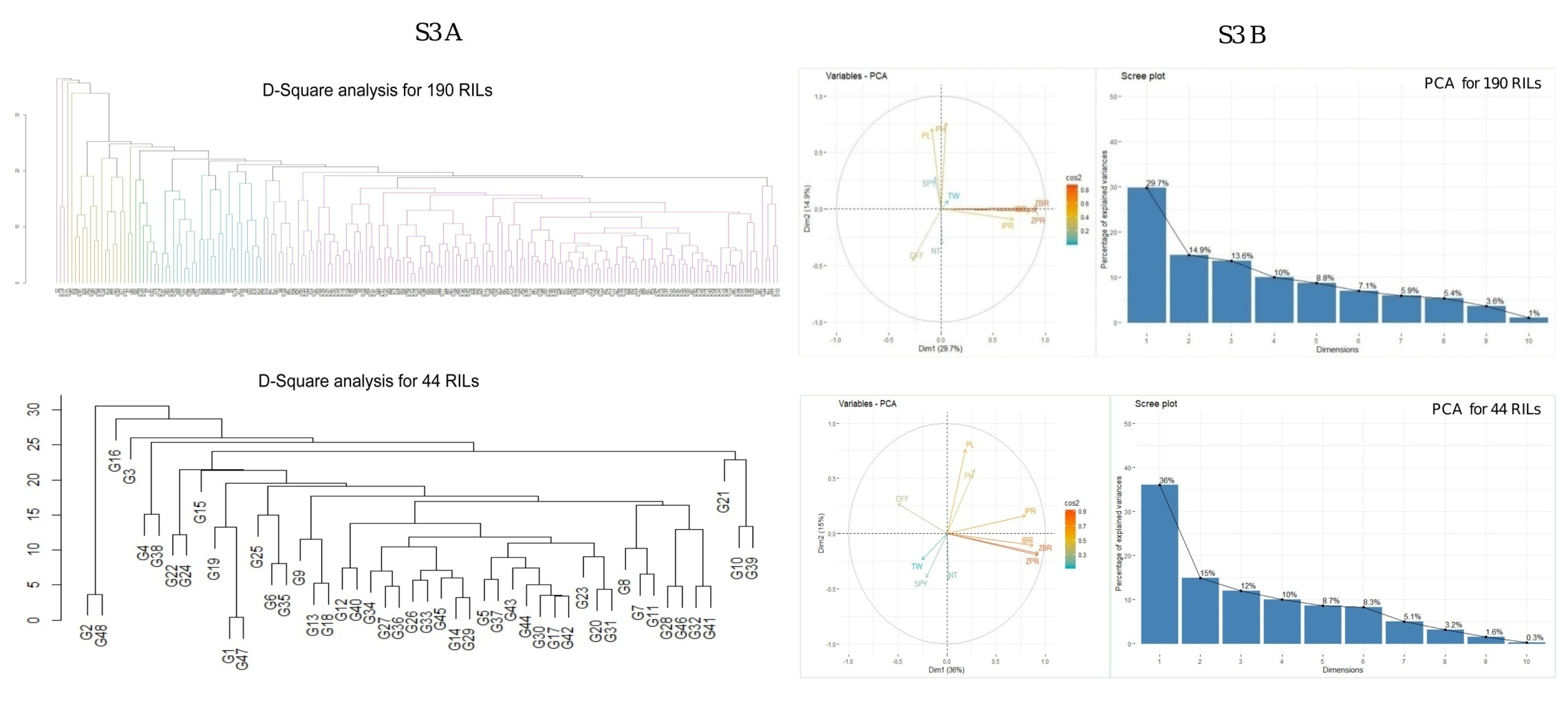
^

Supplementary Figure 6: PCA of 190 and 44 RILs for ten traits based on morphological characters.


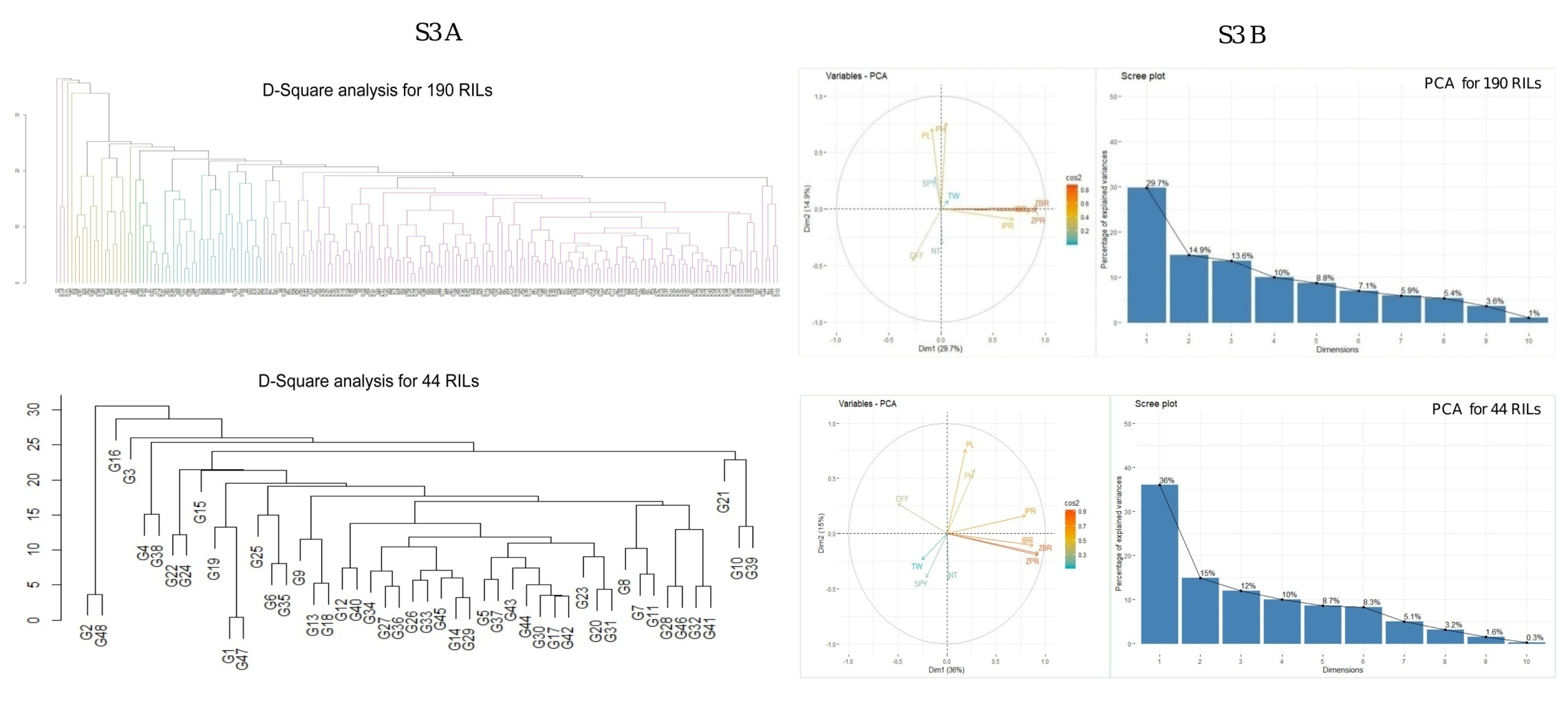


Supplementary Figure 7: AMMI and GGE biplot for ZBR in different Environments: ZBR-AMMI biplot, ZBR-GGE biplot, ZBR-Mean *vs* Stability and ZBR-Which Won Where/What.

| ^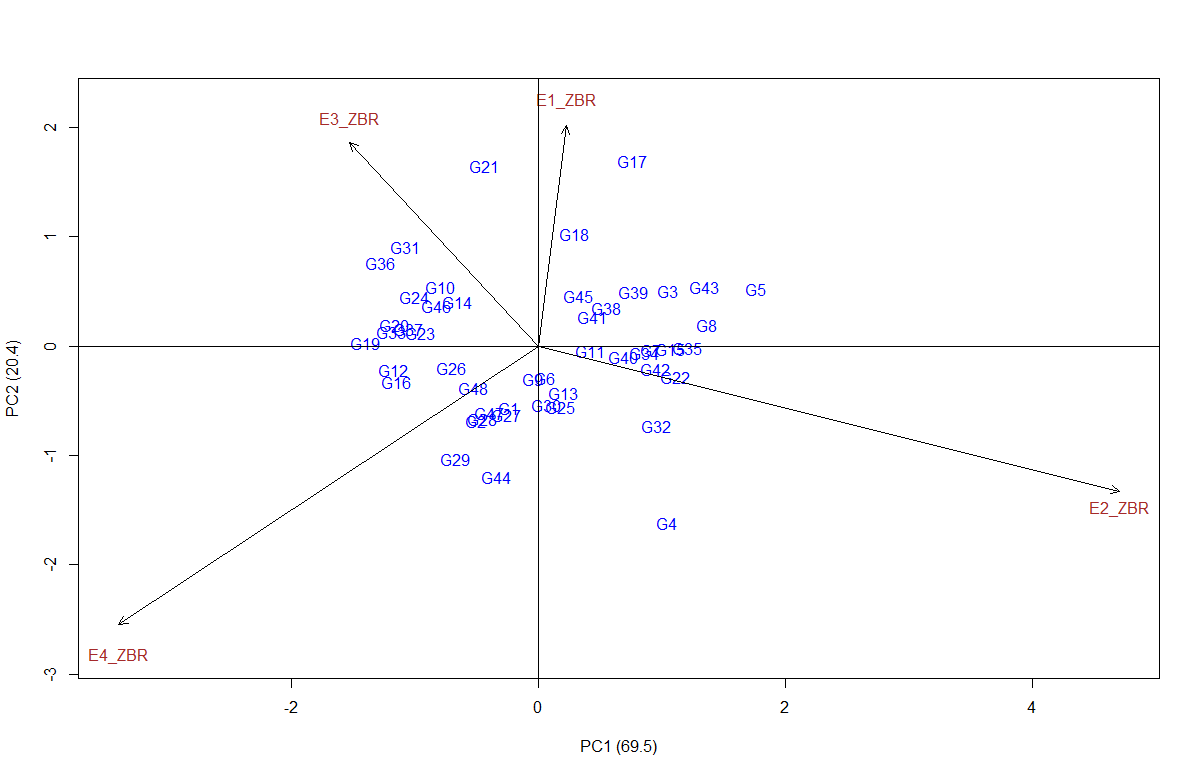^ | ^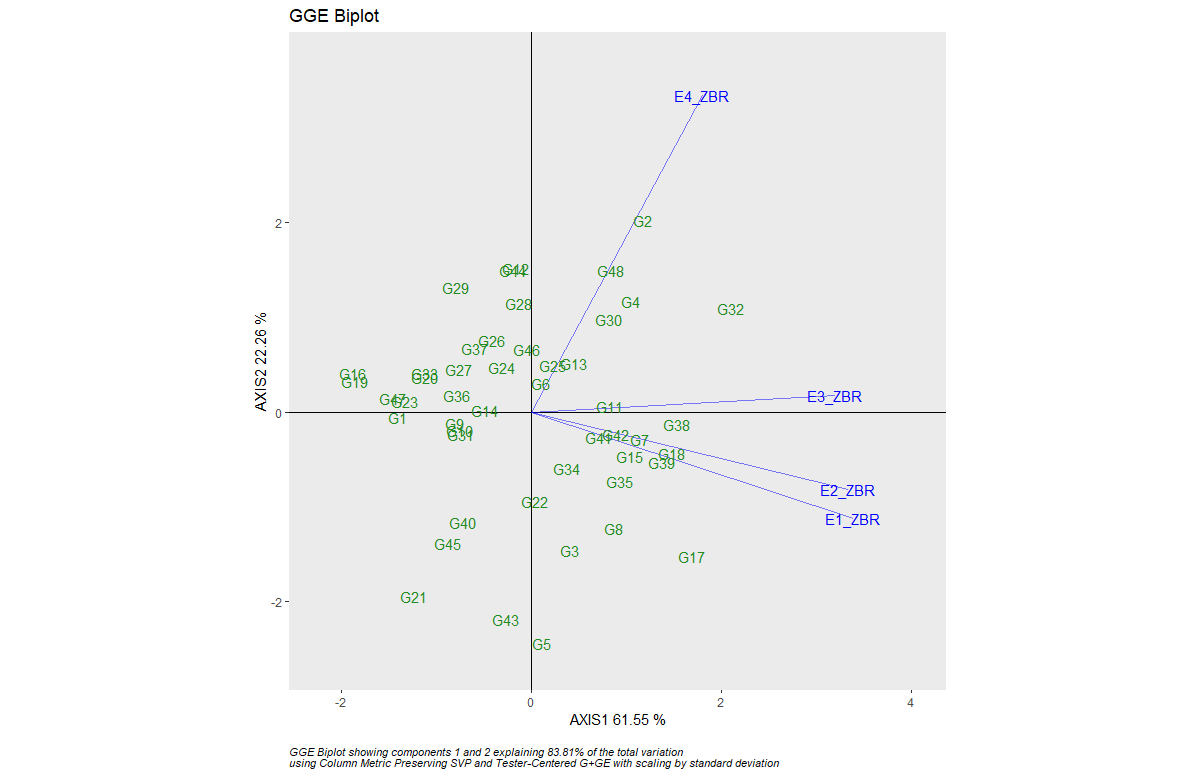^ |
| --- | --- |
| ^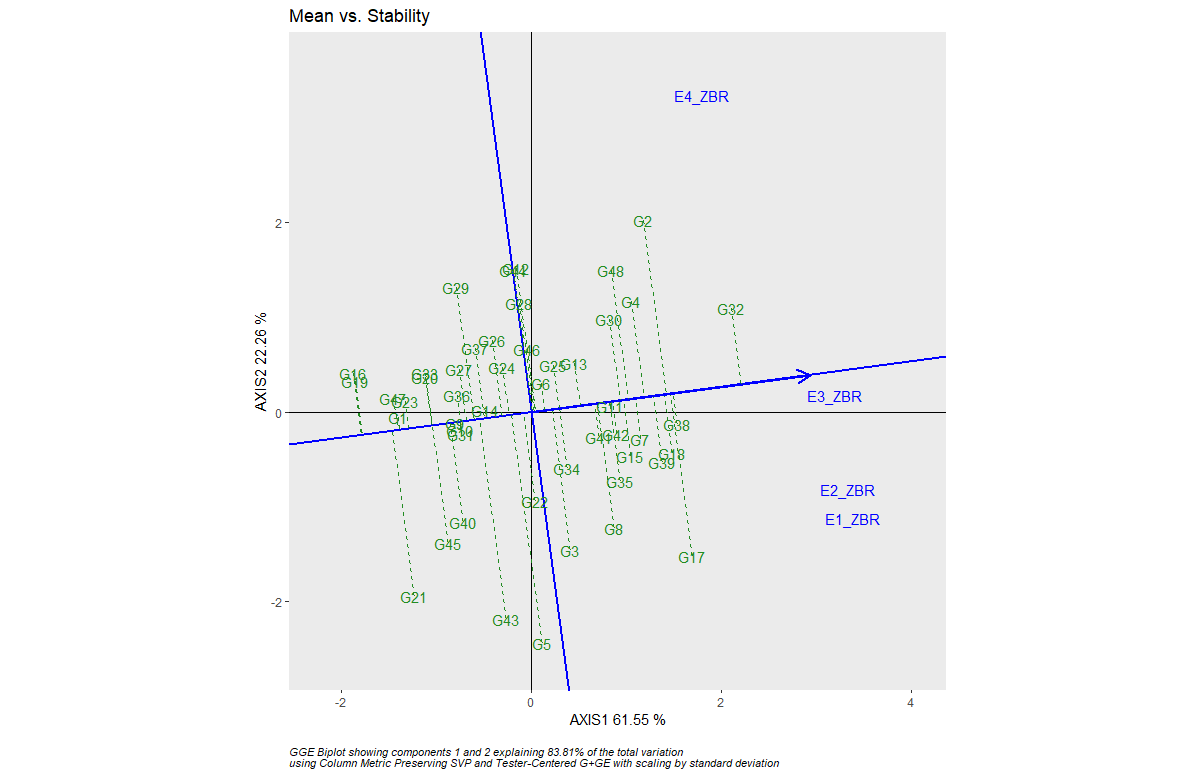^ | ^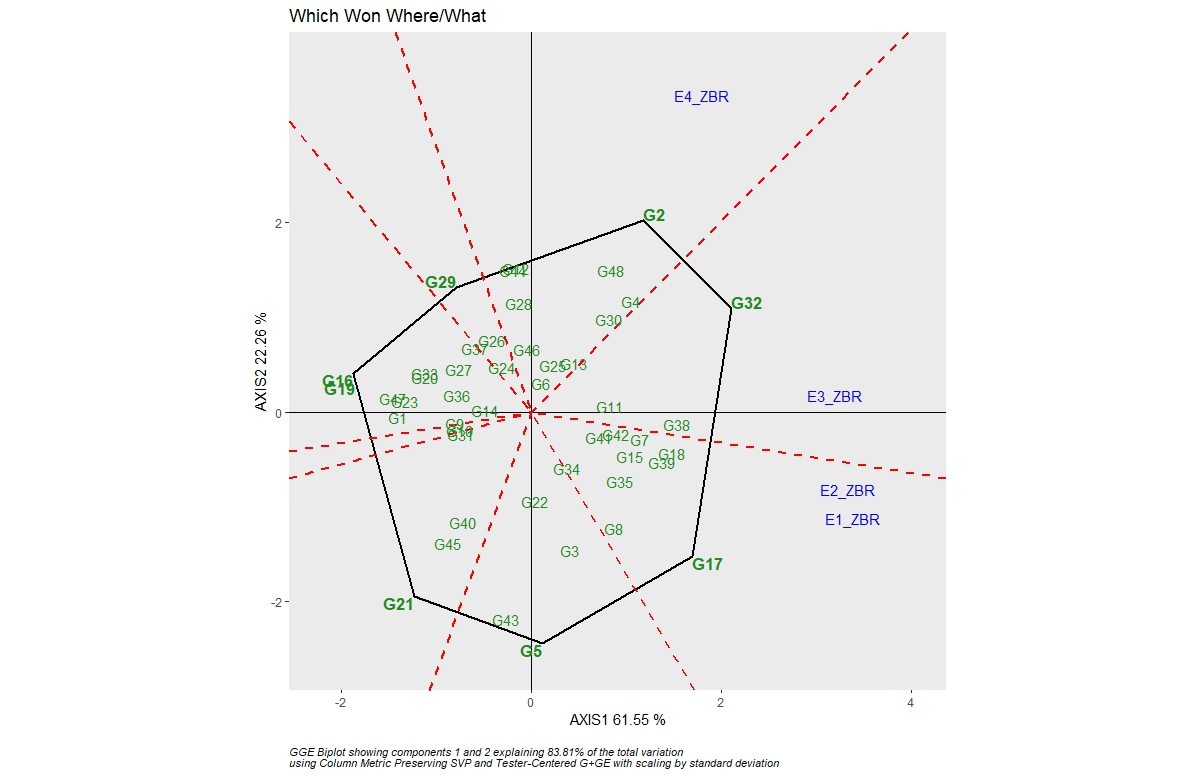^ |

Supplementary Figure 8: AMMI and GGE biplot for IPR in different Environments: IPR-AMMI biplot, IPR-GGE biplot, IPR-Mean *vs* Stability and IPR-Which Won Where/What.

| ^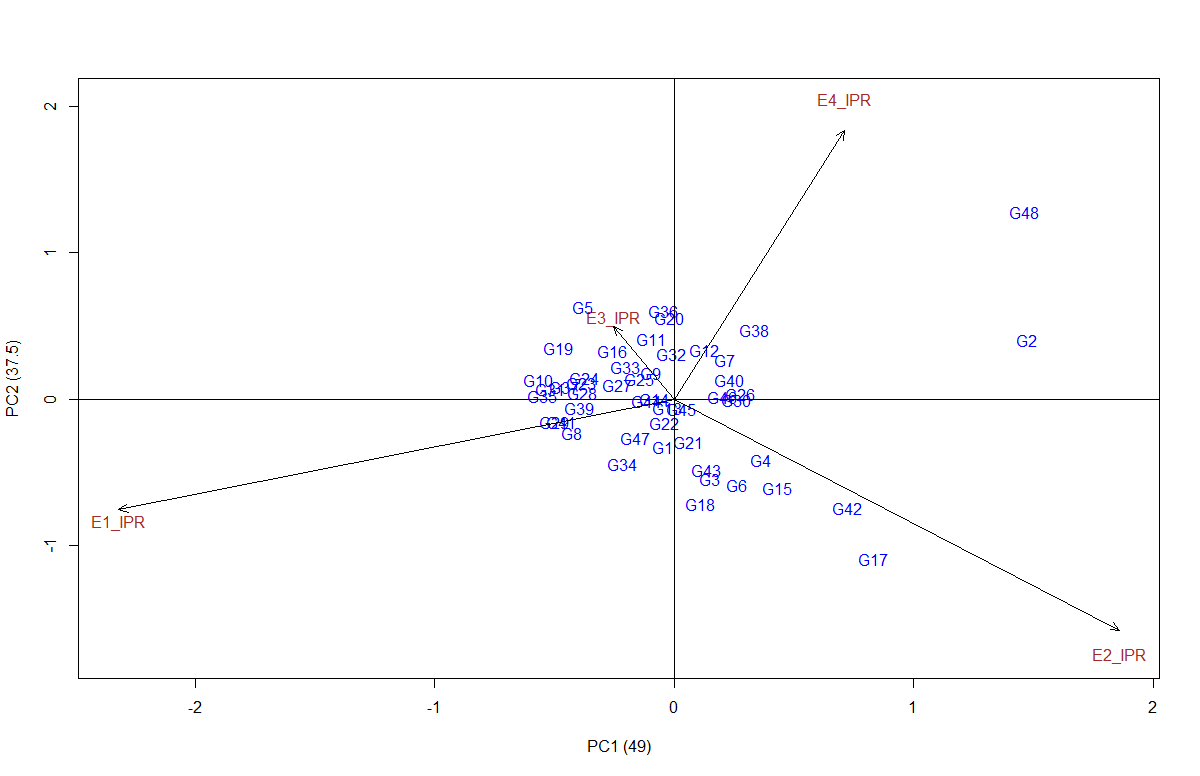^ | ^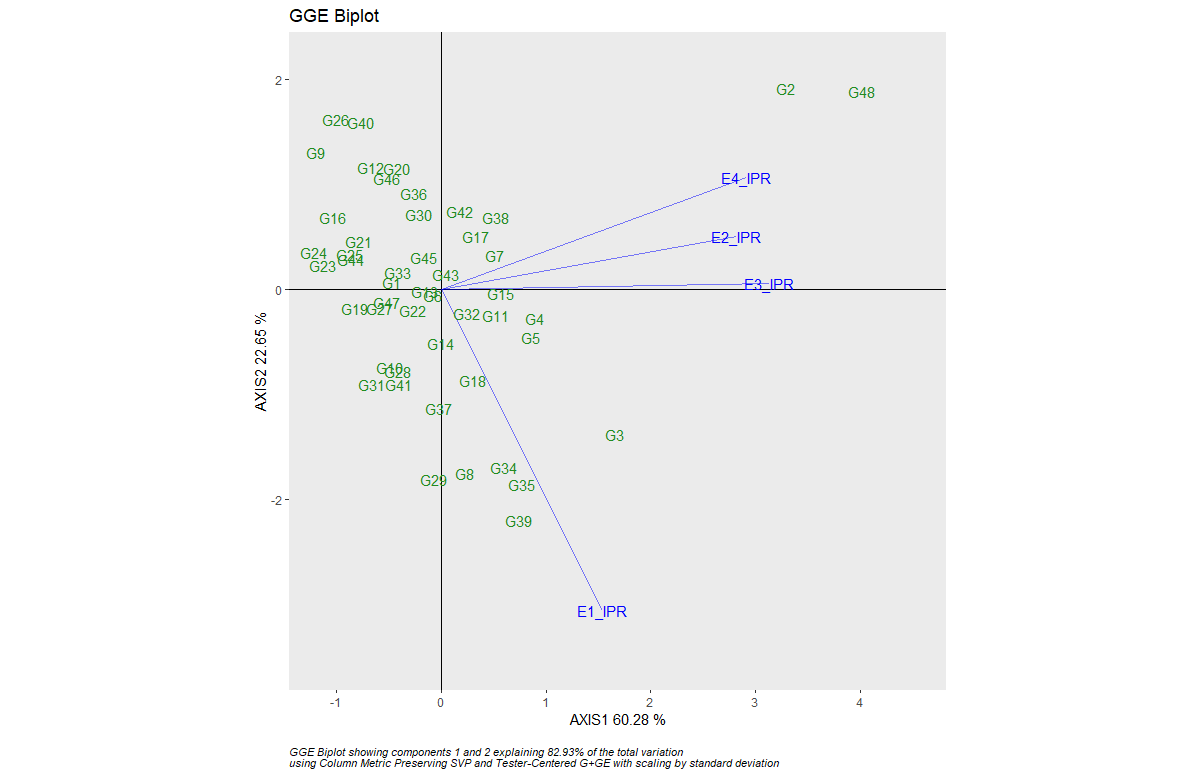^ |
| --- | --- |
| ^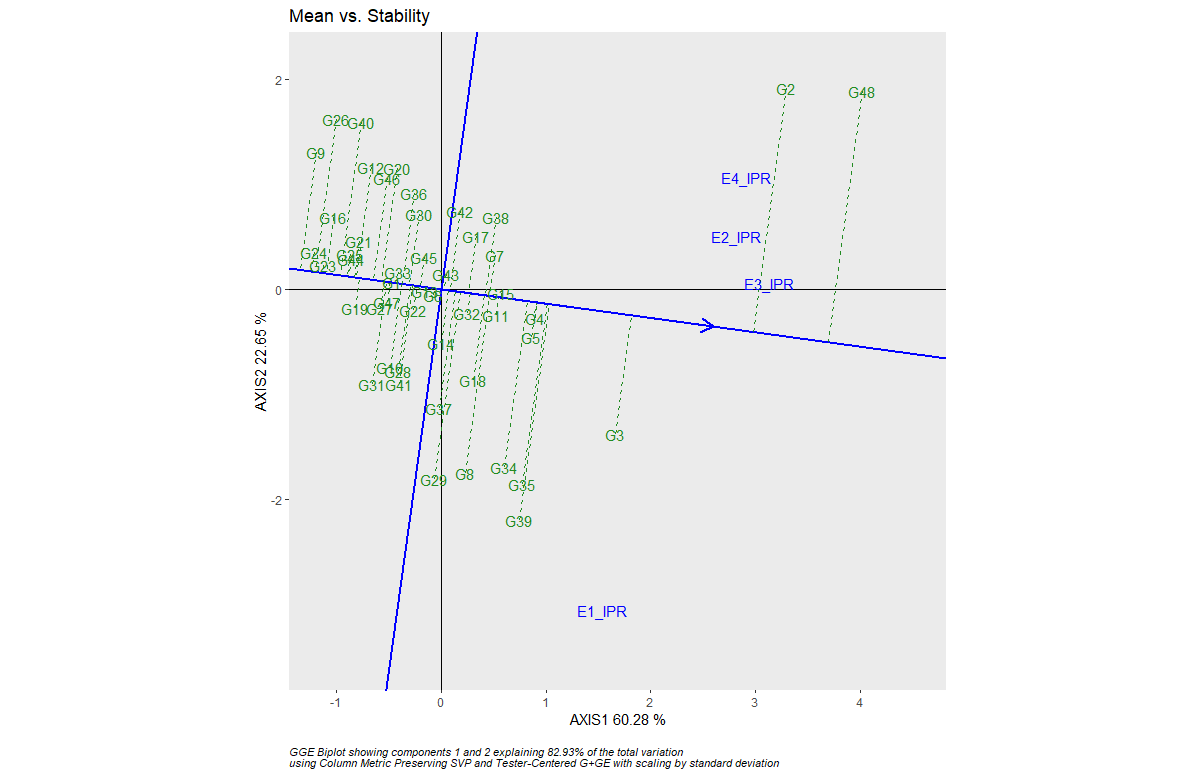^ | ^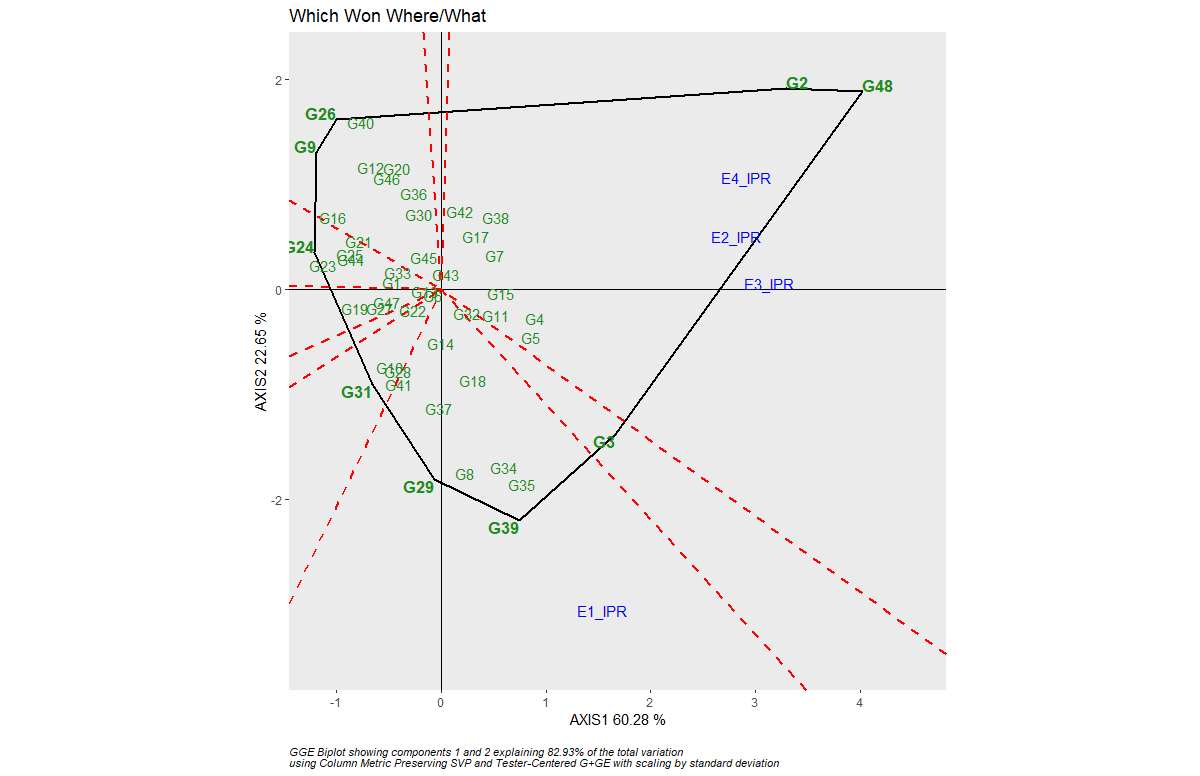^ |

Supplementary Figure 9: AMMI and GGE biplot for IBR in different Environments: IBR-AMMI biplot, IBR-GGE biplot, IBR-Mean *vs* Stability and IBR-Which Won Where/What.

| ^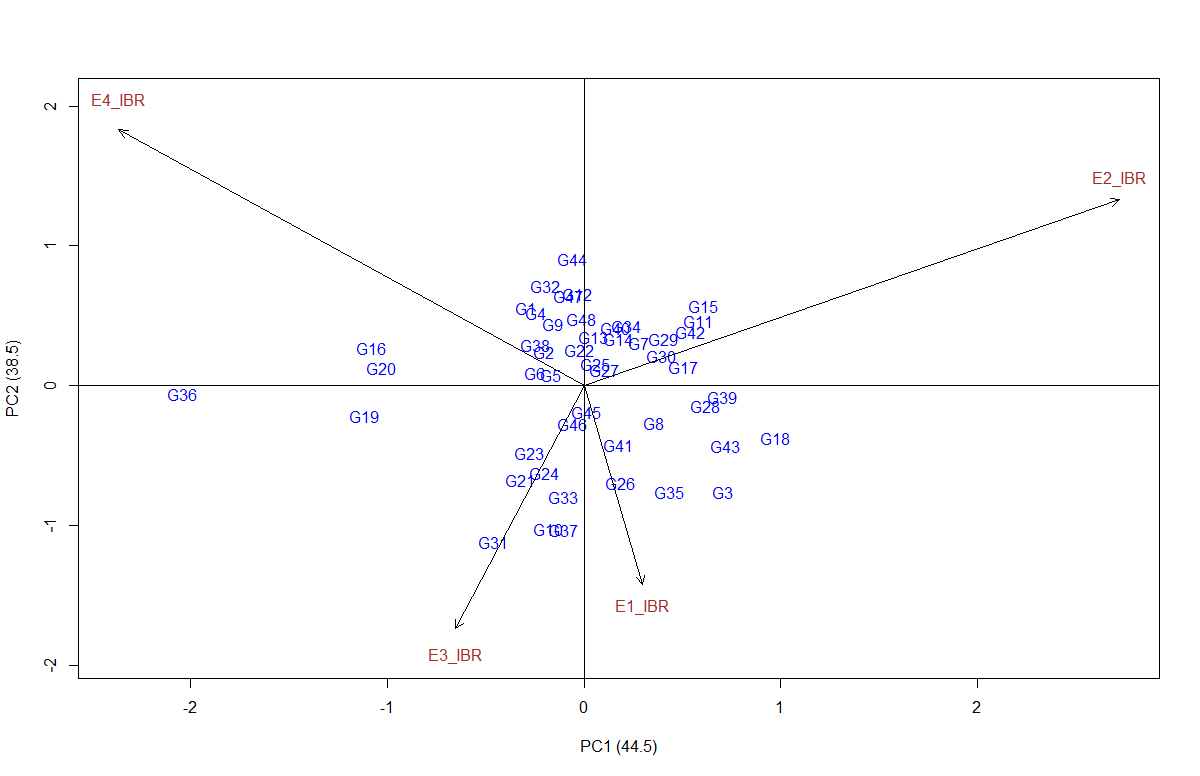^ | ^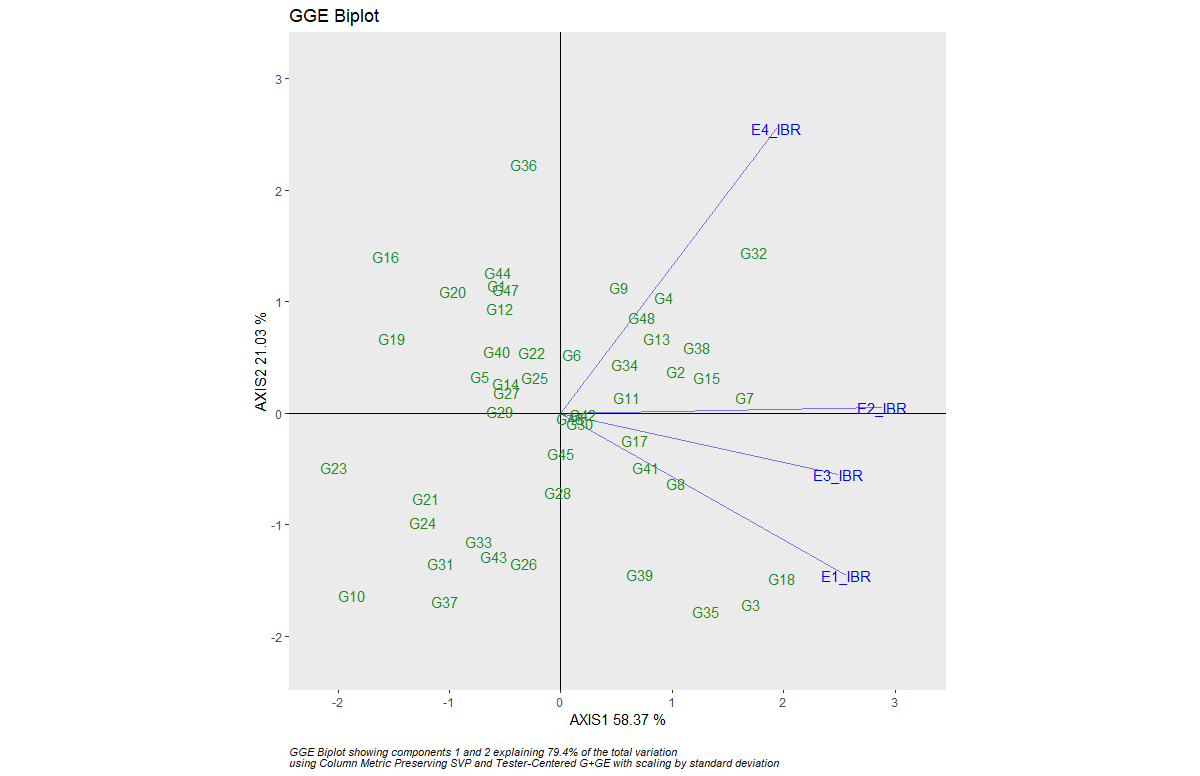^ |
| --- | --- |
| ^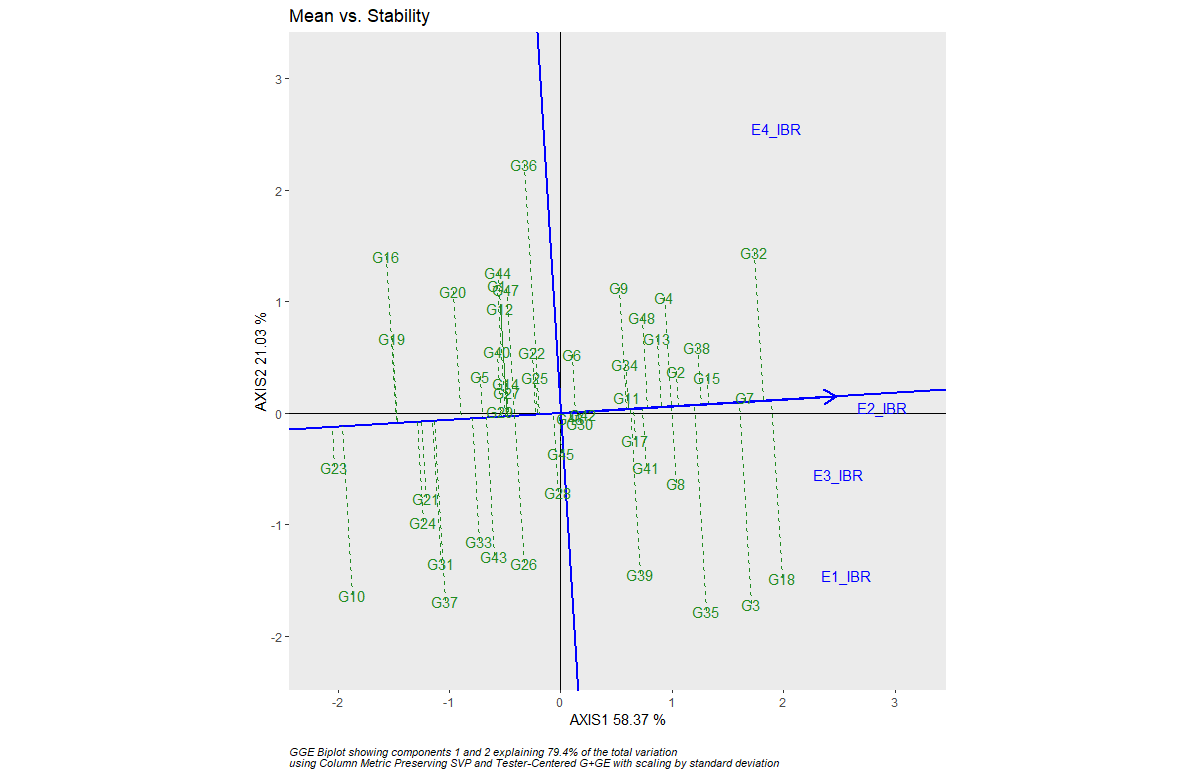^ | ^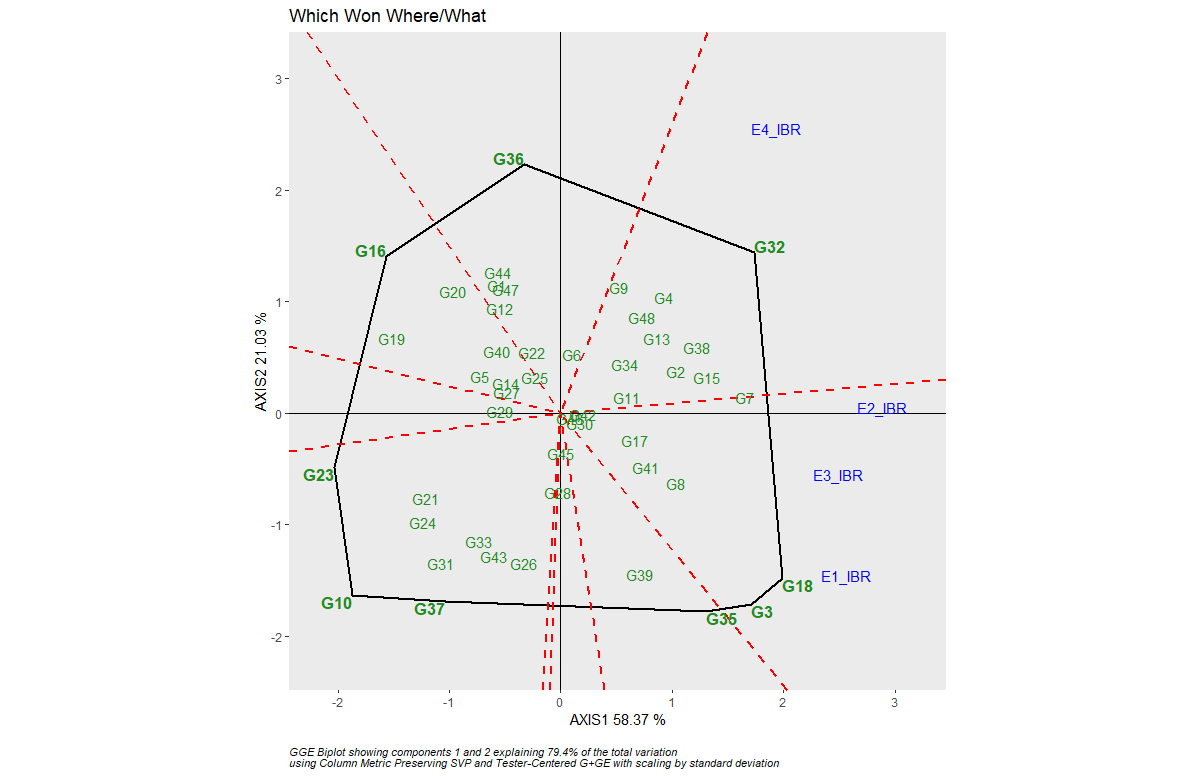^ |

Supplementary Figure 10: AMMI and GGE biplot for TW in different Environments: TW-AMMI biplot, TW-GGE biplot, TW-Mean *vs* Stability and TW-Which Won Where/What.

| ^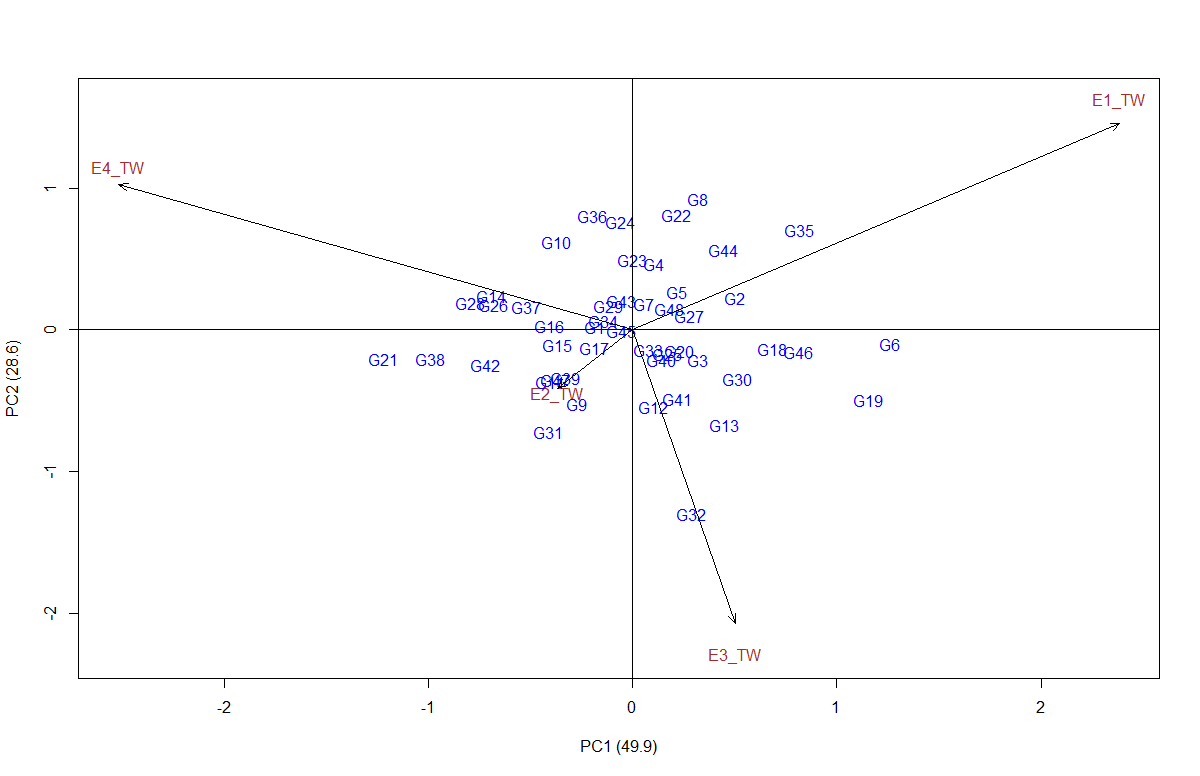^ | ^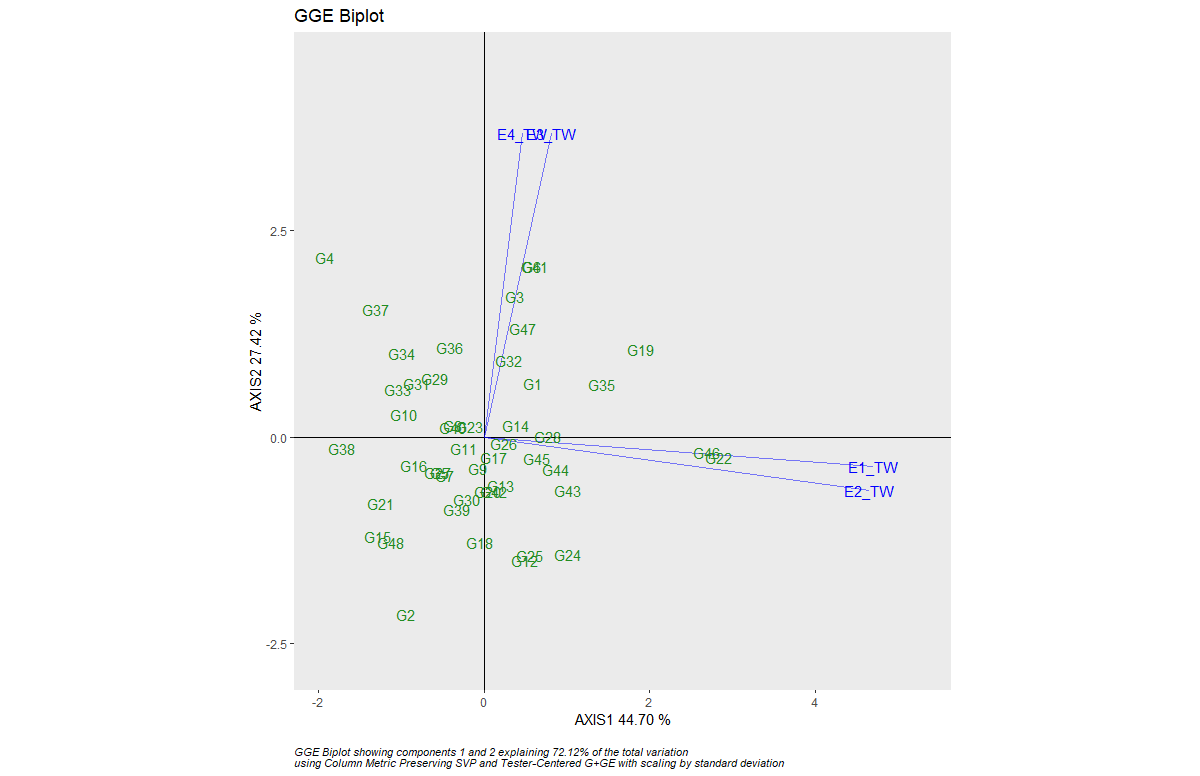^ |
| --- | --- |
| ^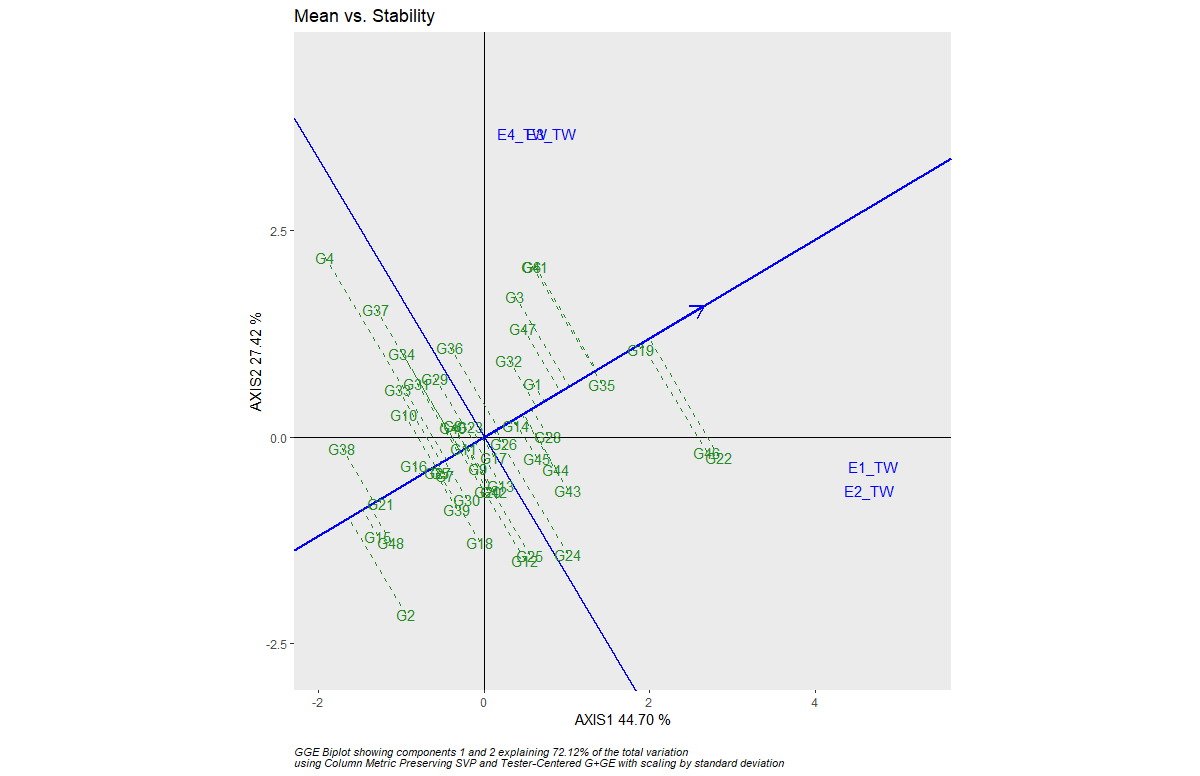^ | ^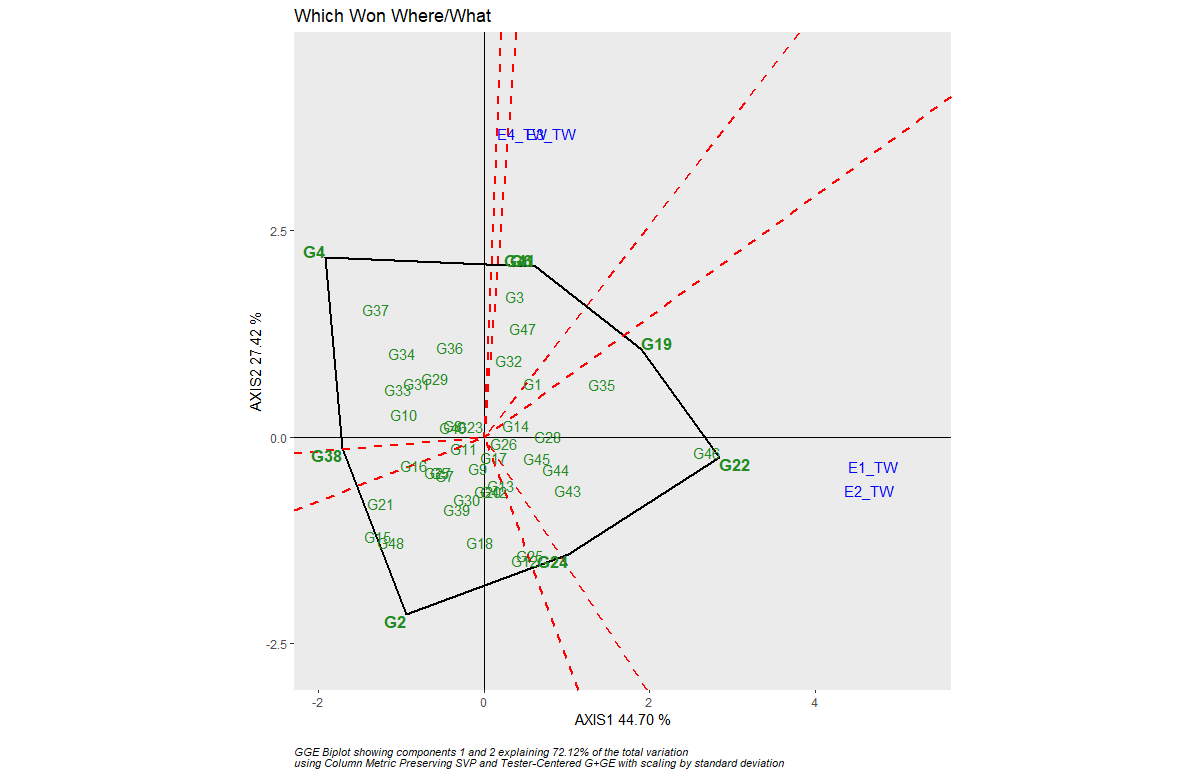^ |

Supplementary Figure 11: AMMI and GGE biplot for PH in different Environments: PH-AMMI biplot, PH-GGE biplot, PH-Mean *vs* Stability and PH-Which Won Where/What.

| ^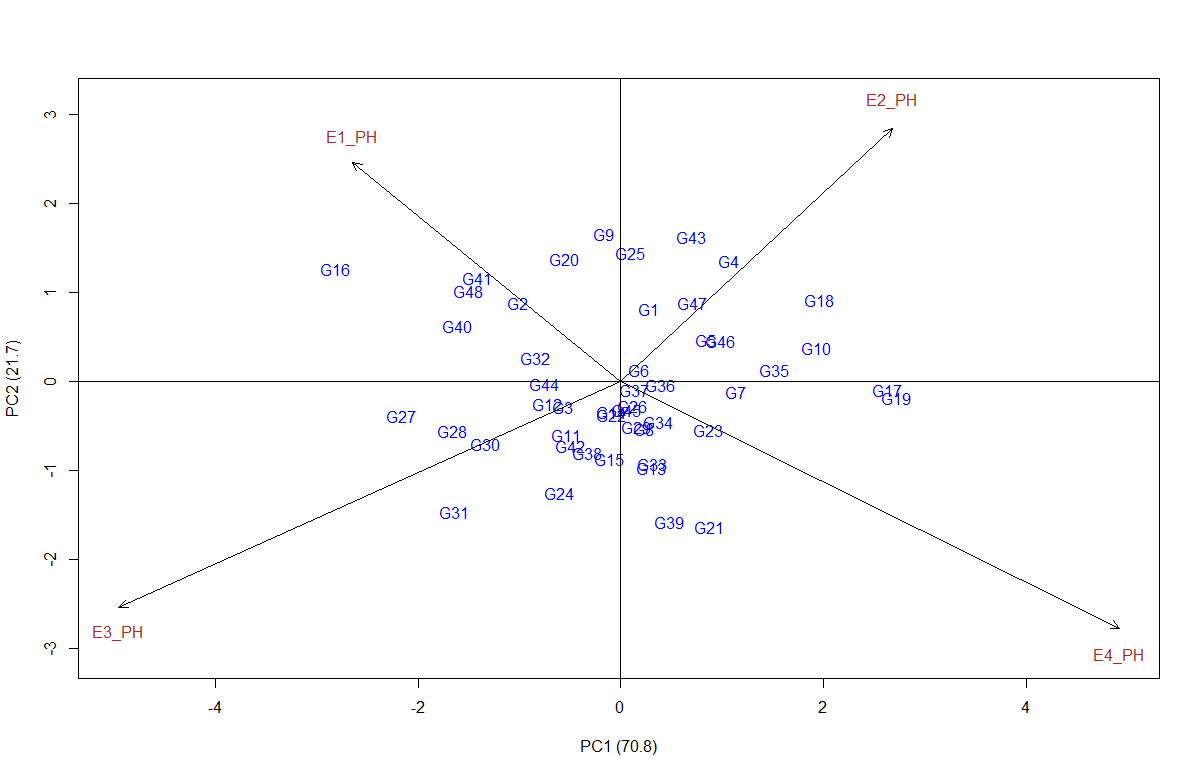^ | ^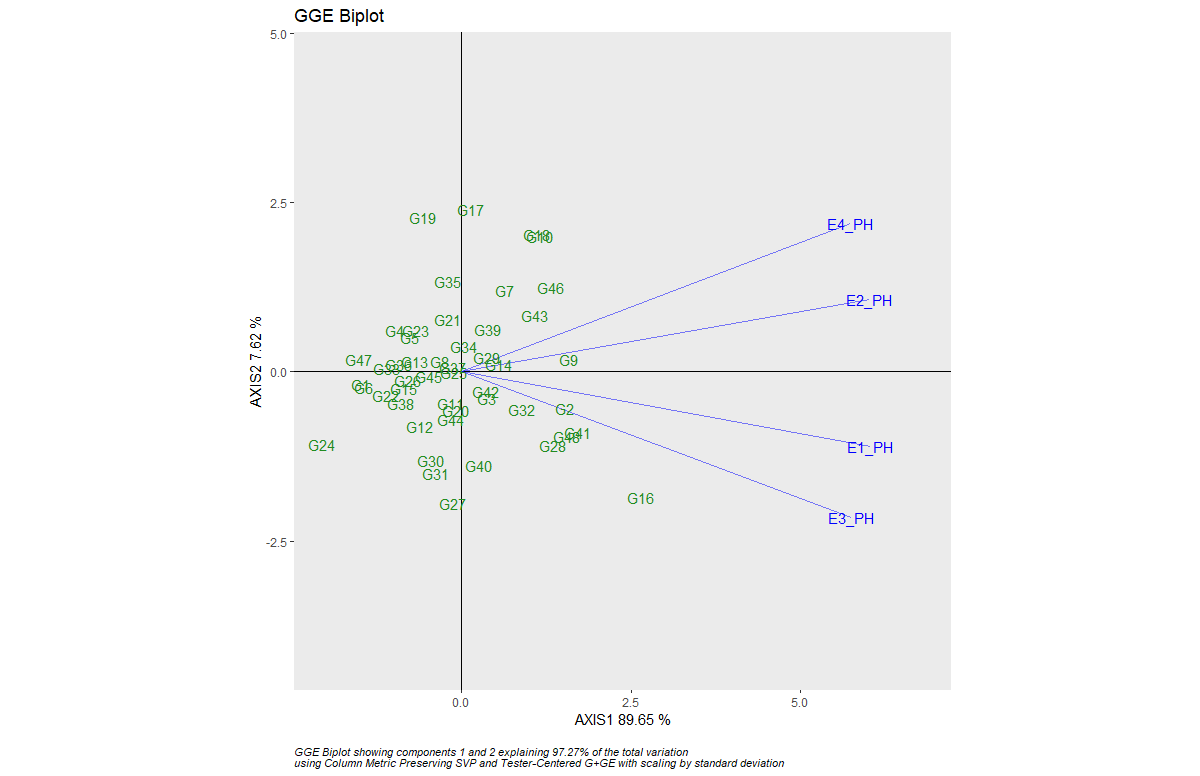^ |
| --- | --- |
| ^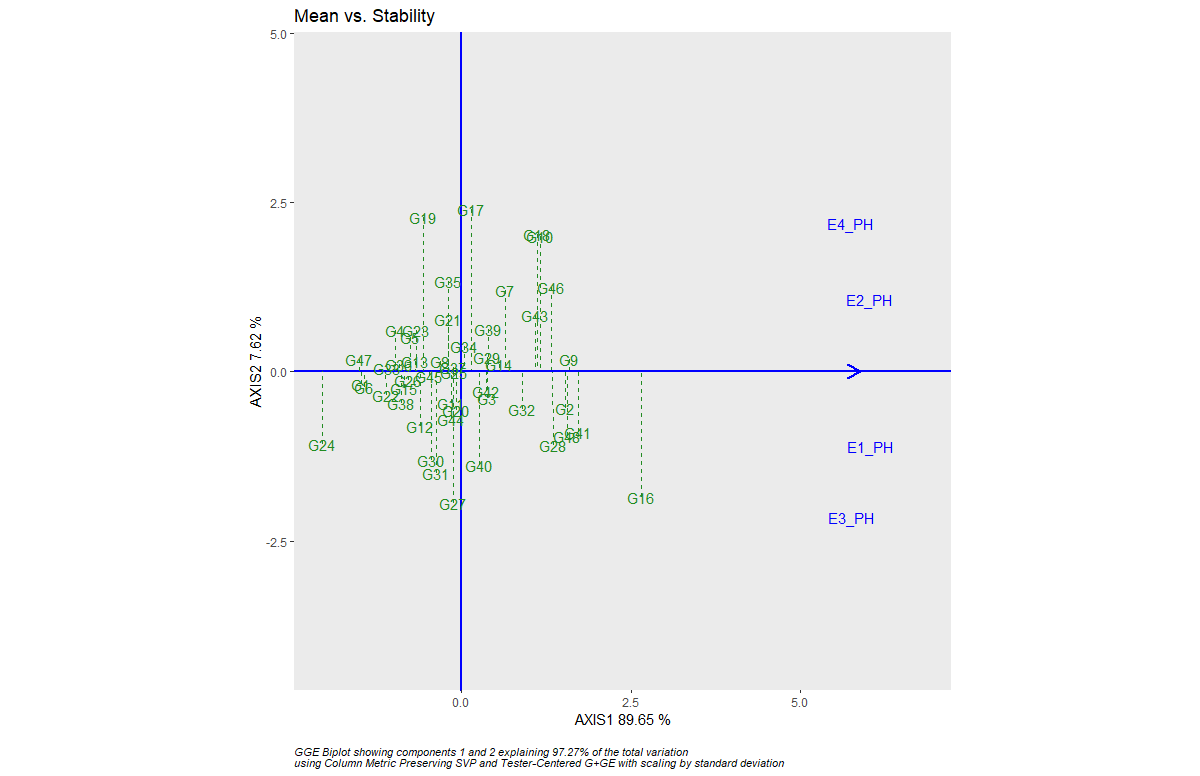^ | ^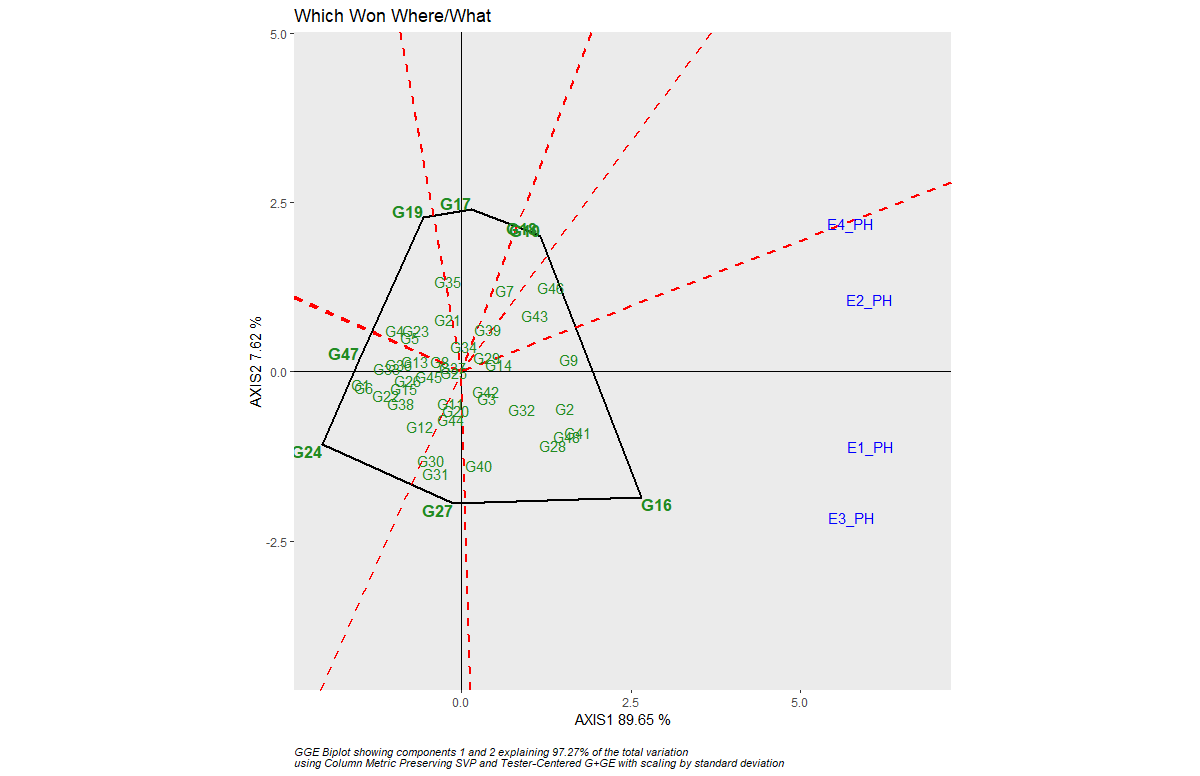^ |

Supplementary Figure 12: AMMI and GGE biplot for PL in different Environments: PL-AMMI biplot, PL-GGE biplot, PL-Mean *vs* Stability and PL-Which Won Where/What.

| ^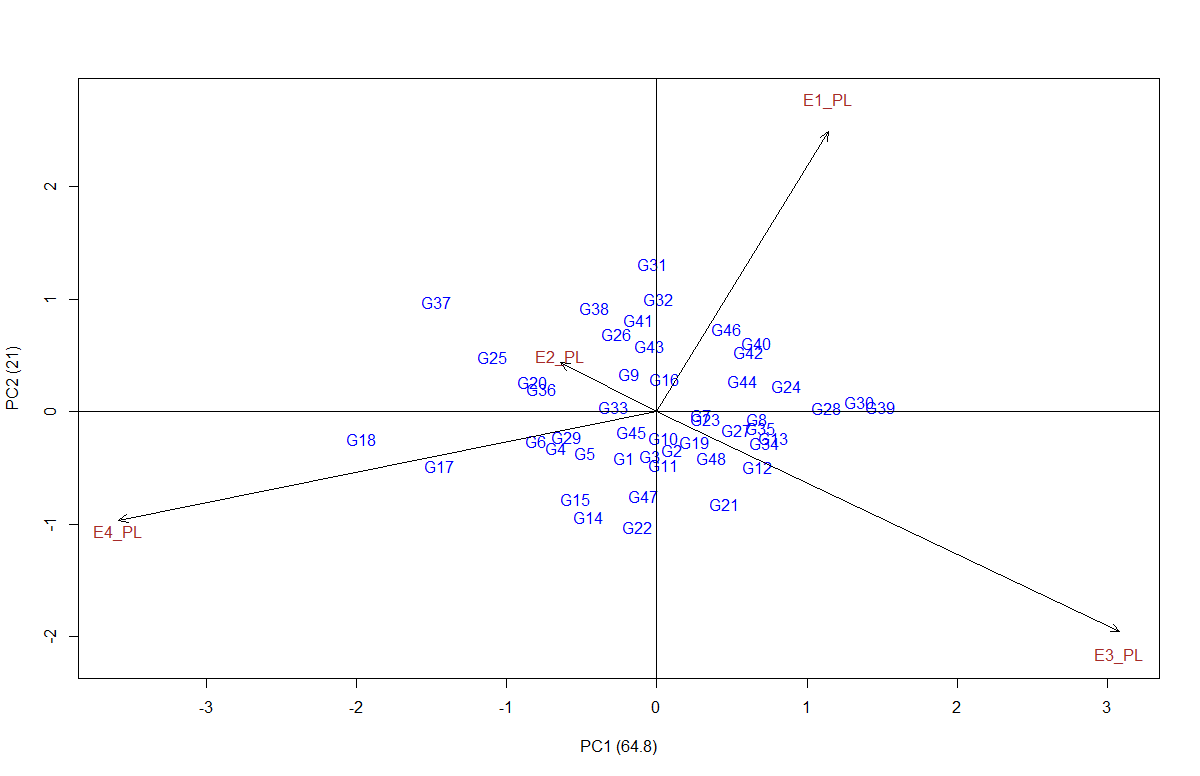^ | ^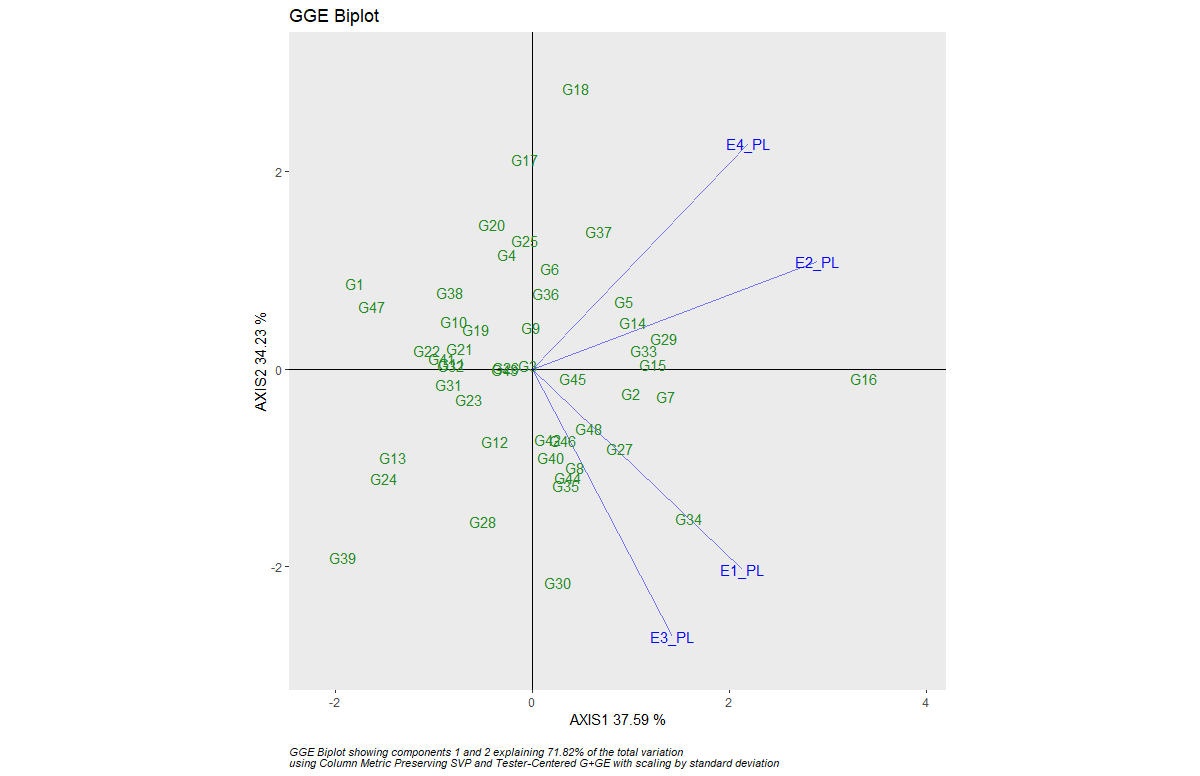^ |
| --- | --- |
| ^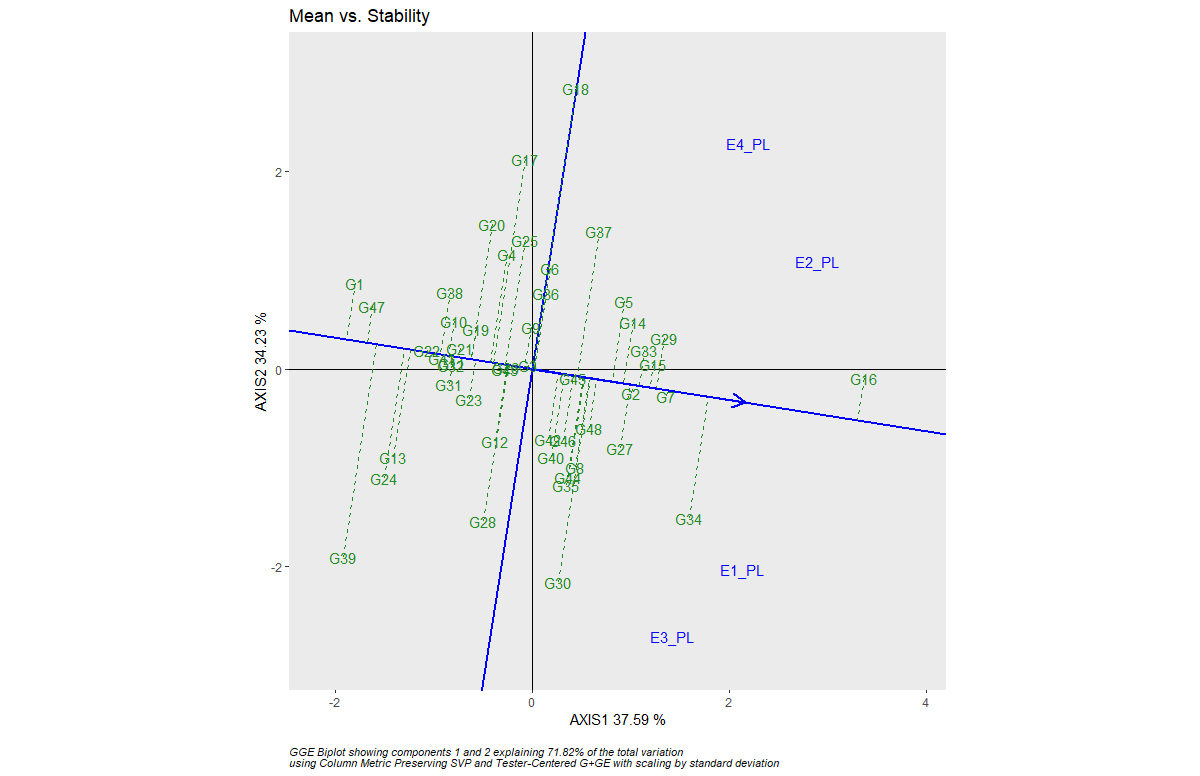^ | ^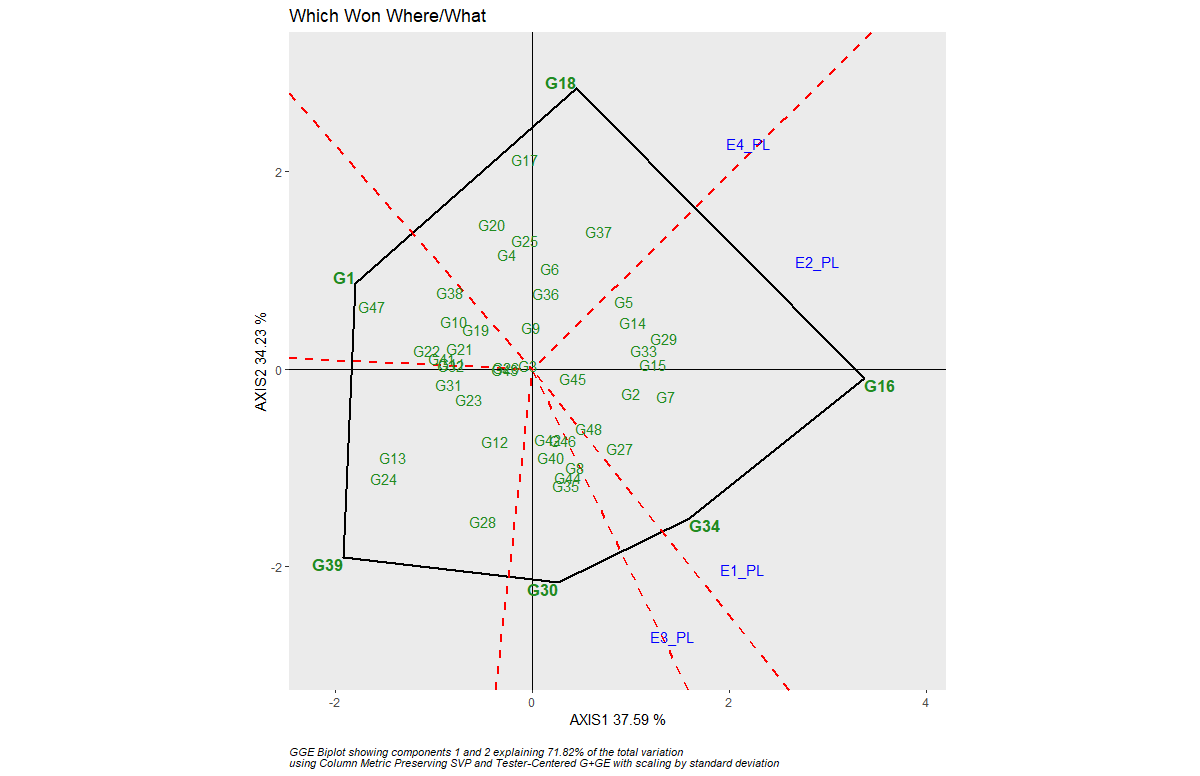^ |

Supplementary Figure 13: AMMI and GGE biplot for NT in different Environments: NT-AMMI biplot, NT-GGE biplot, NT-Mean *vs* Stability and NT-Which Won Where/What

| ^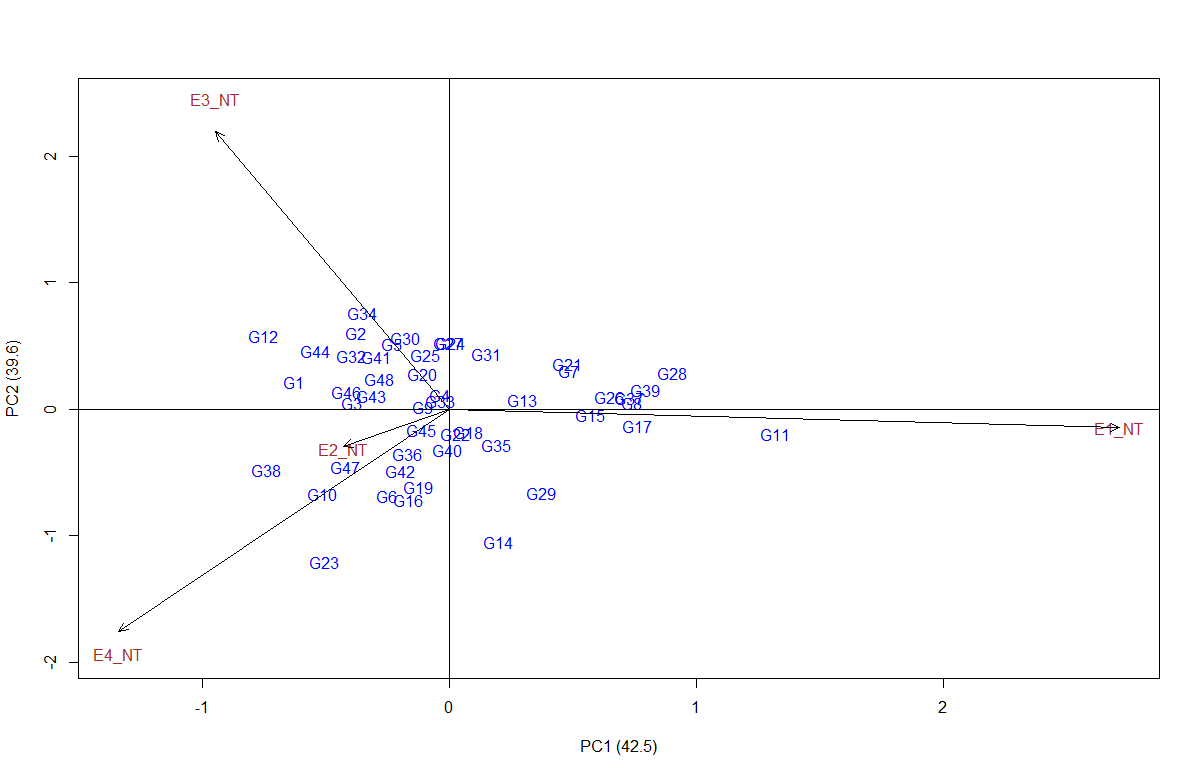^ | ^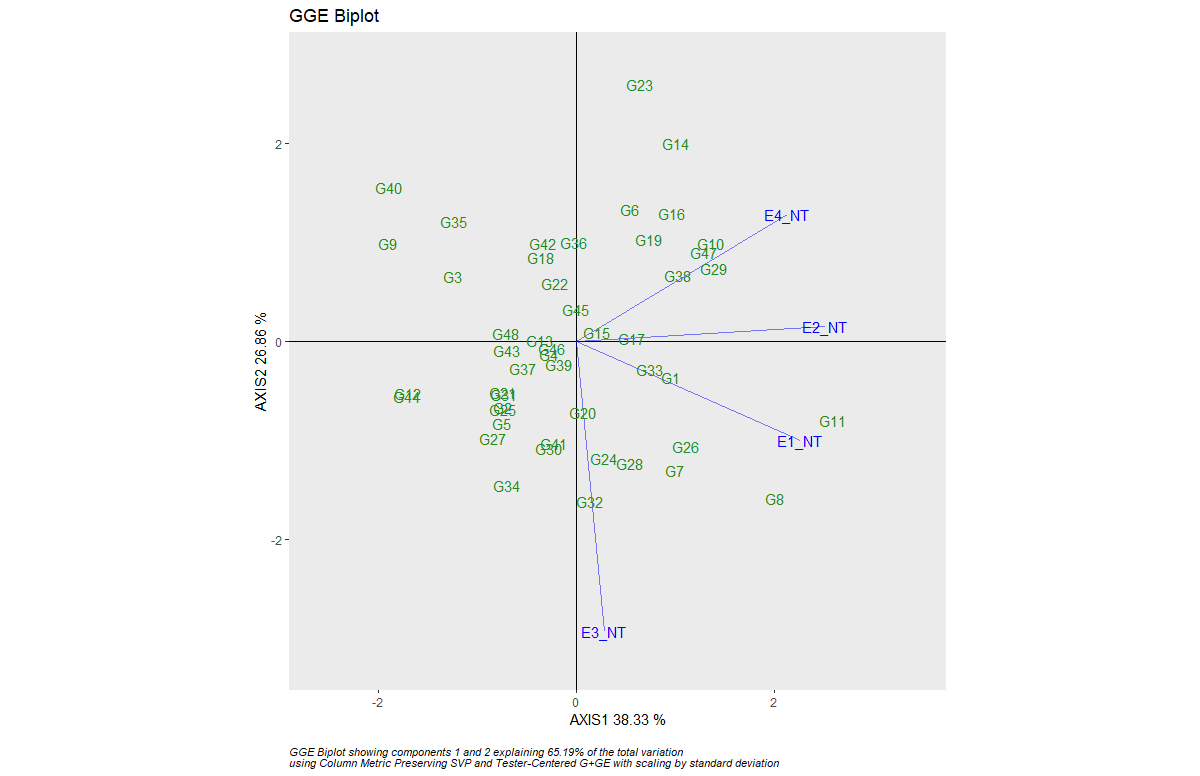^ |
| --- | --- |
| ^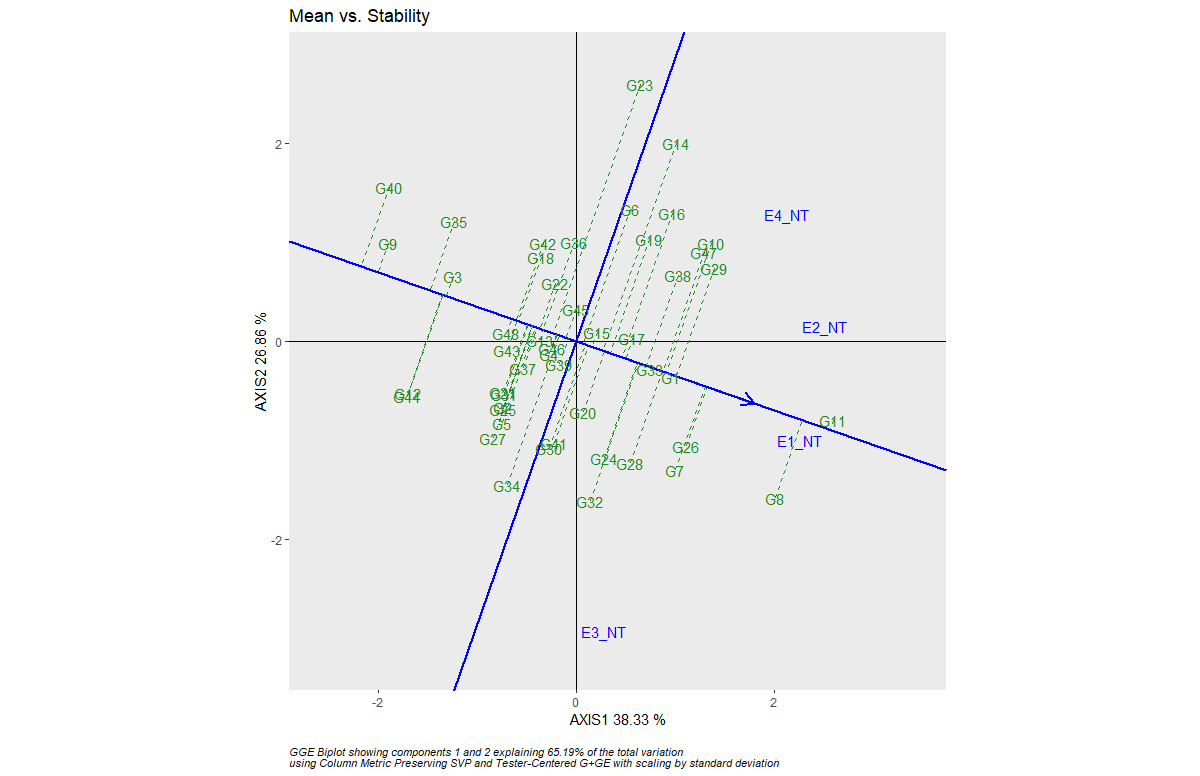^ | ^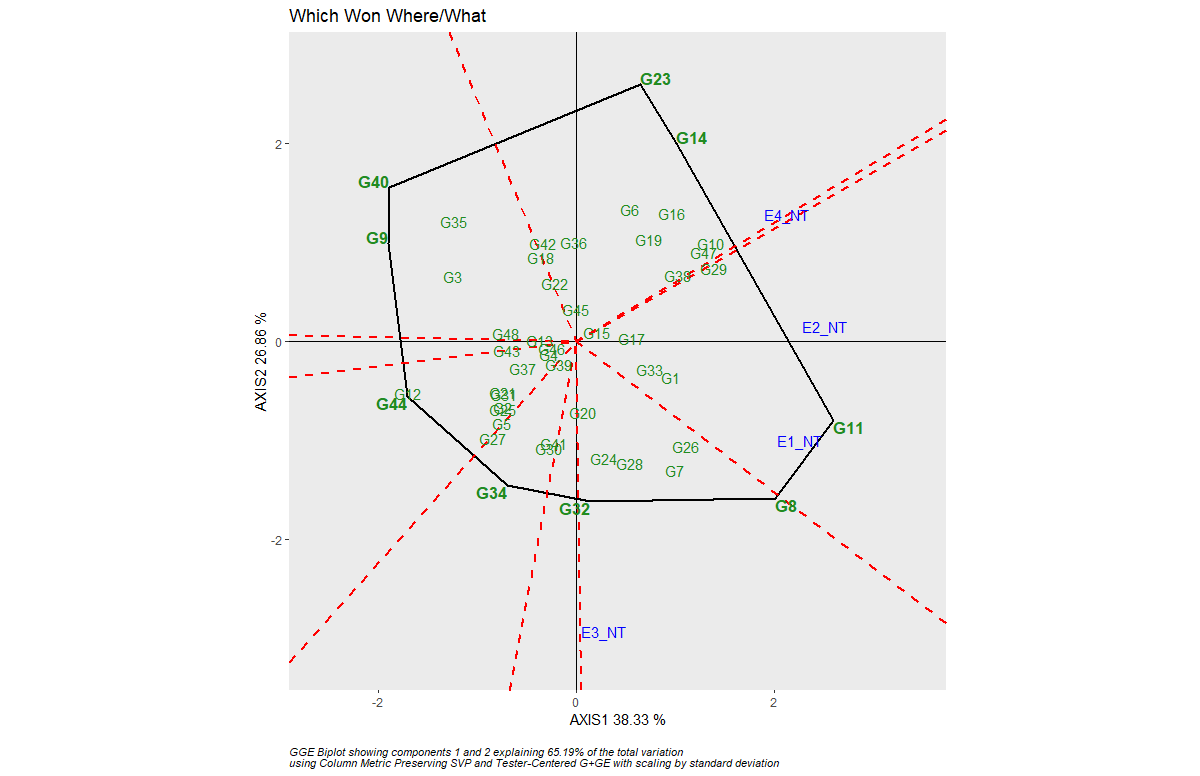^ |

Supplementary Figure 14: AMMI and GGE biplot for DFF in different Environments: DFF-AMMI biplot, DFF-GGE biplot, DFF-Mean *vs* Stability and DFF-Which Won Where/What

| ^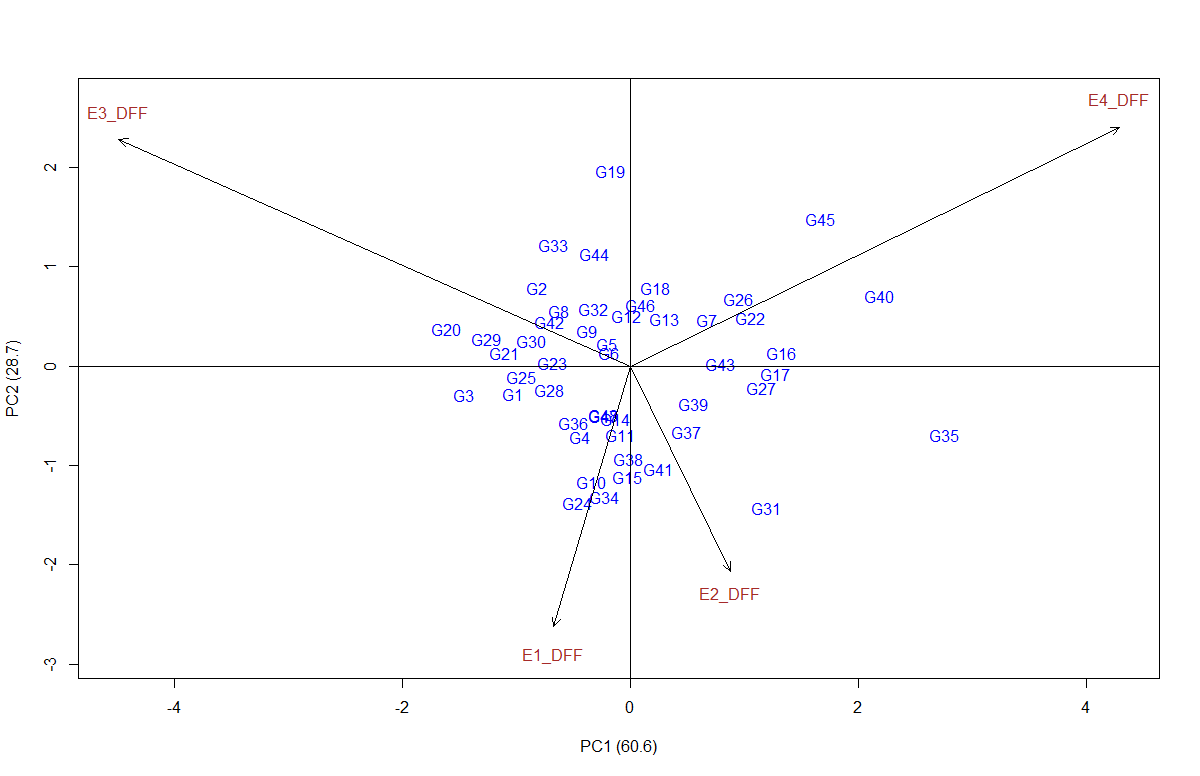^ | ^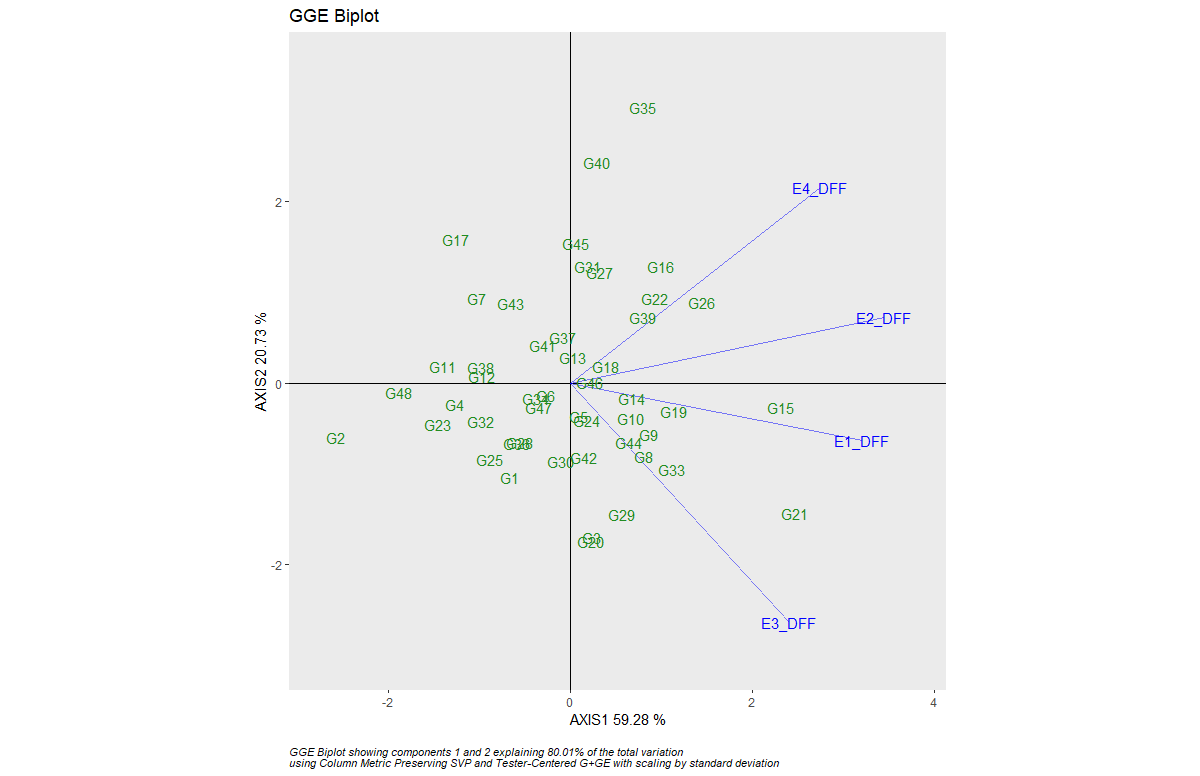^ |
| --- | --- |
| ^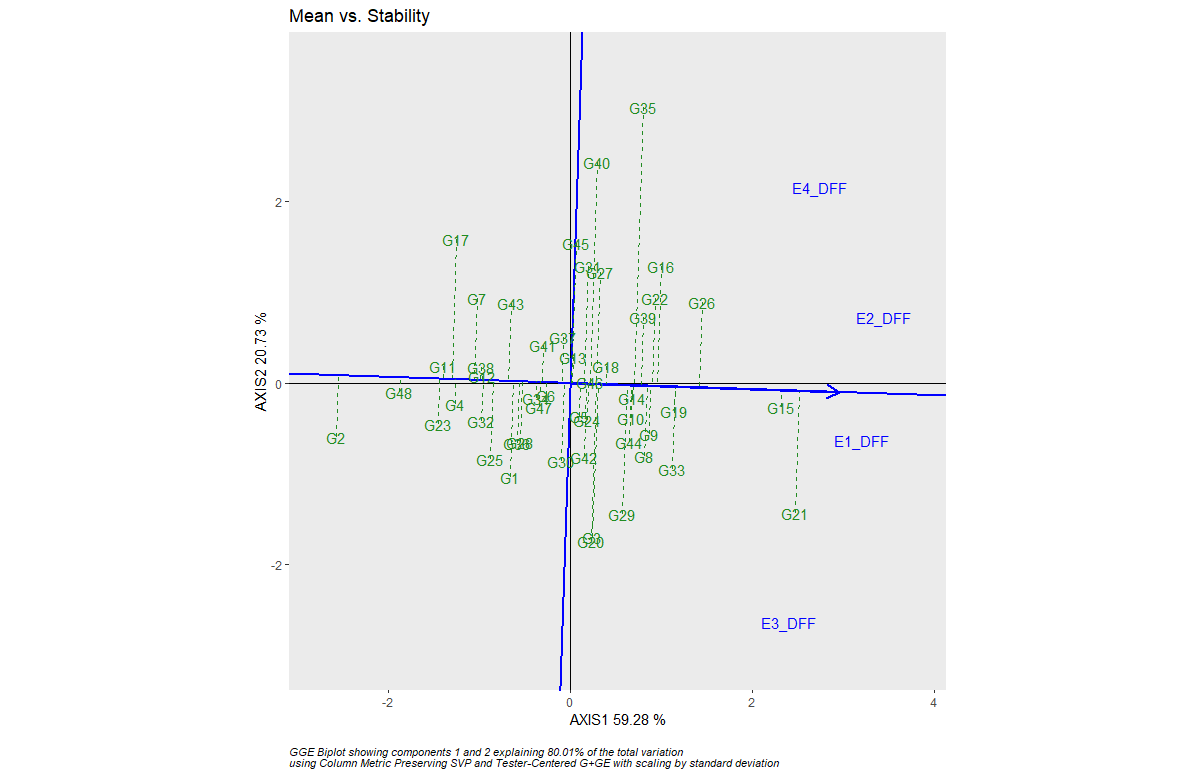^ | ^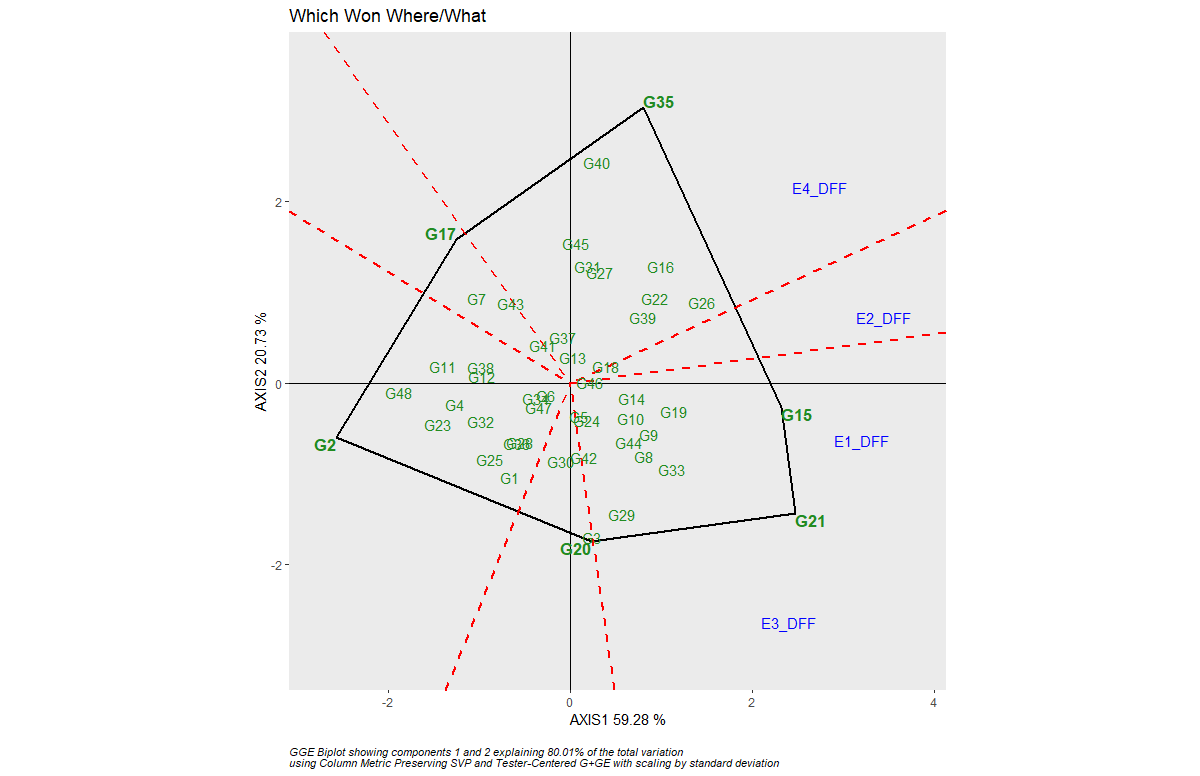^ |

Supplementary Figure 15: Identified Candidate genes in the QTL regions (*qZPR.1.1, qAOC_ZPR.1.1* and *qZBR.1.1*on chromosome 1; *qZPR.11.1* and *qAOC_ZPR.11.1* on chromosome 11) by using WEGO analysis.

| *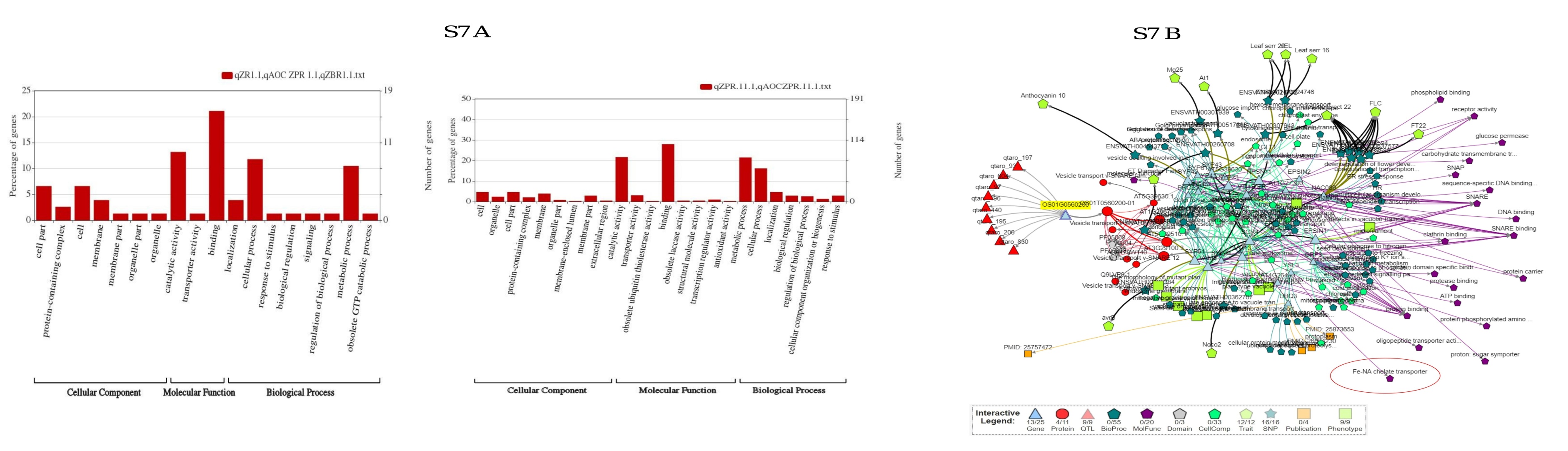* | *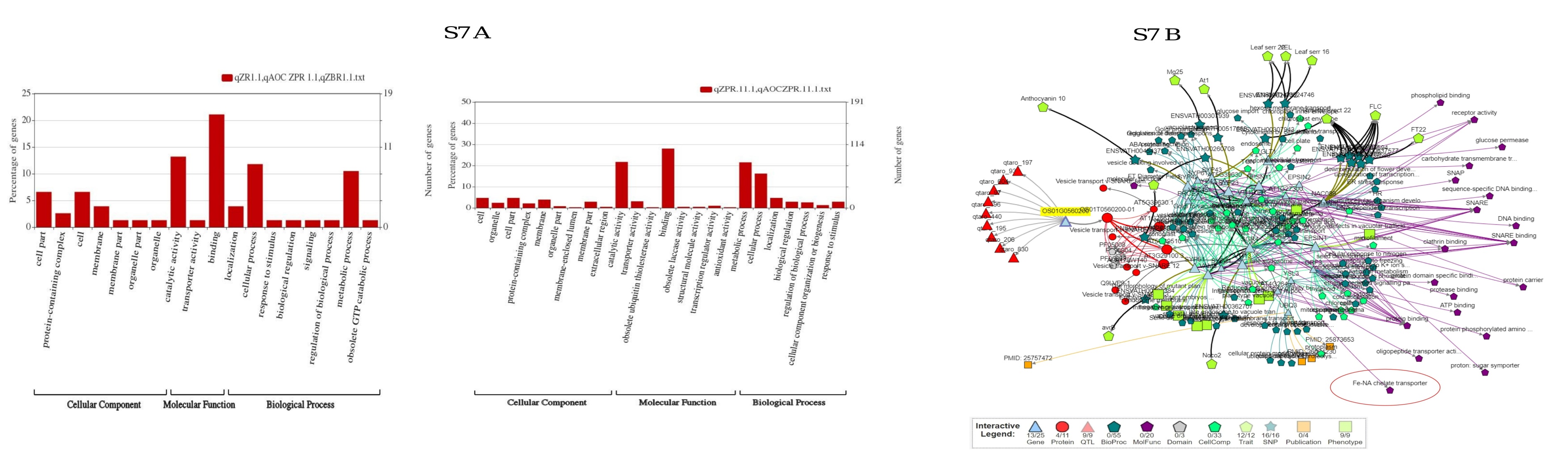* |
| --- | --- |

Supplementary Figure 16: Identified two putative candidate genes and their temporal and spatial expression

| 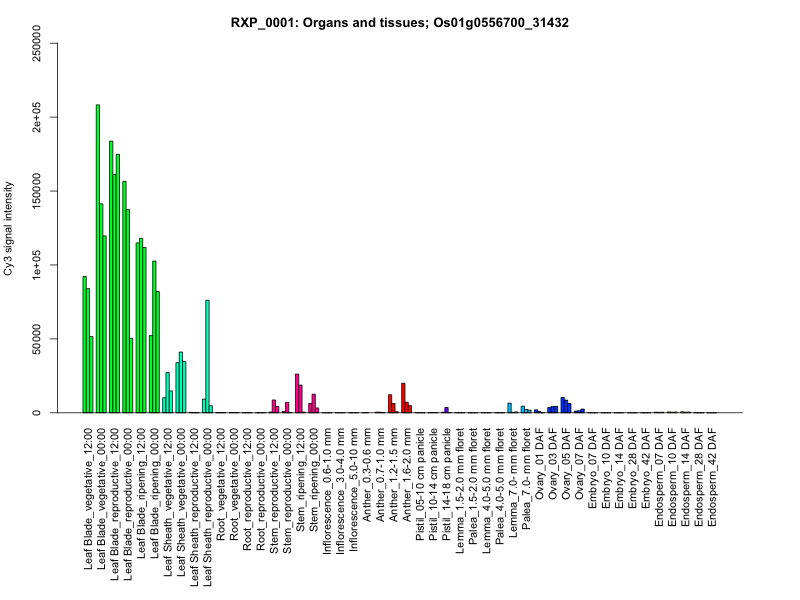 | 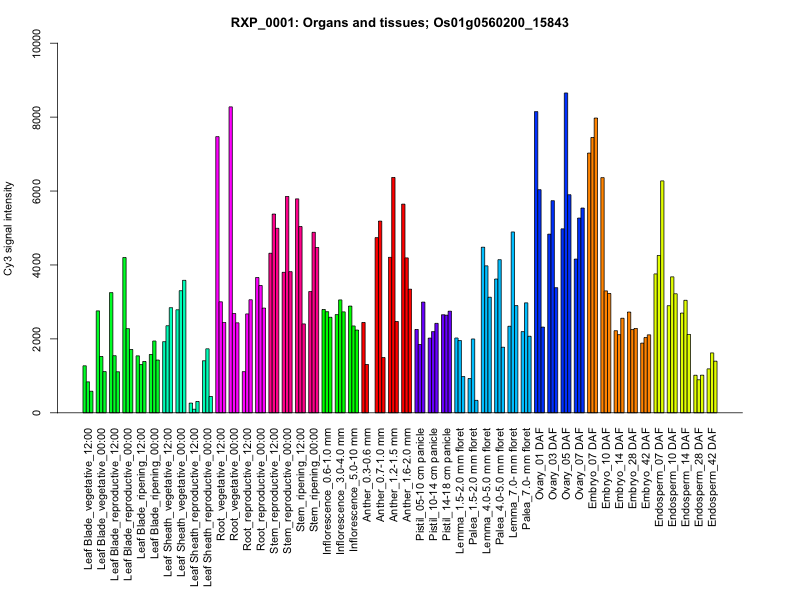 |
| --- | --- |

Supplementary Figure 17: Candidate gene (*Os01g0560200*-vesicle transport v-SNARE protein, putative, expressed positioned) network by using KNet miner.

.^
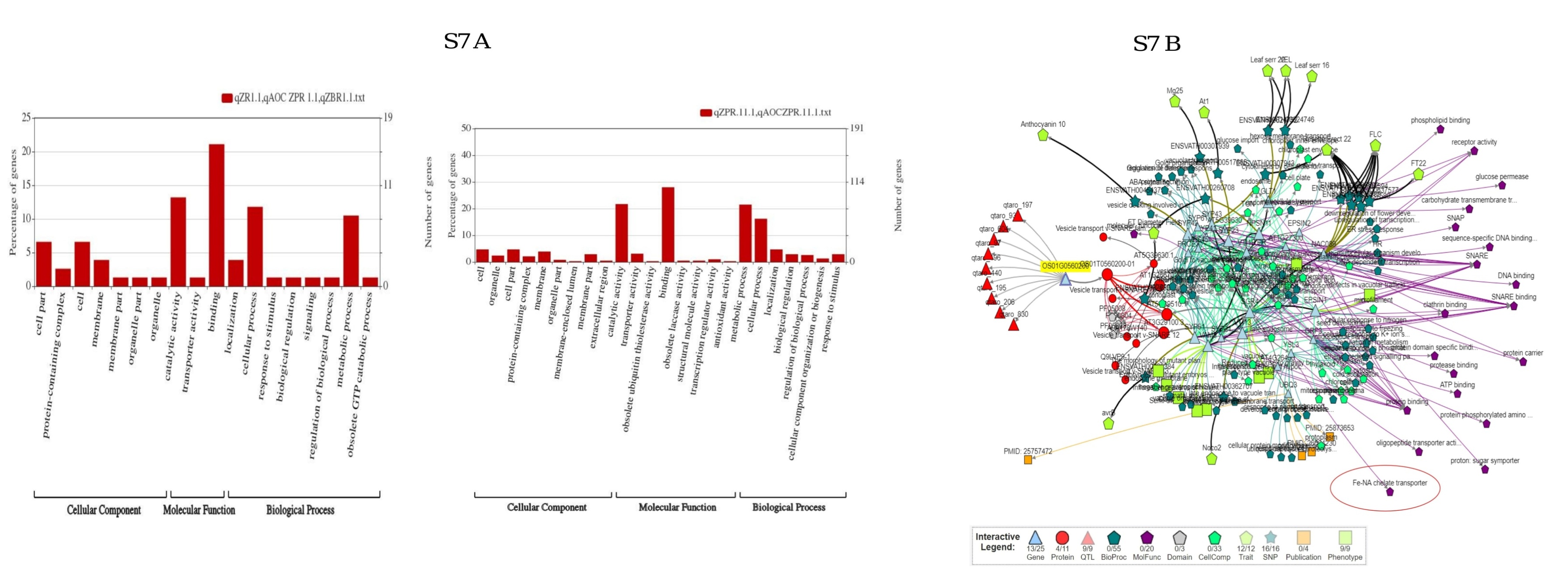
^
